# A translatable RNAi-driven gene therapy silences *PMP22/Pmp22* genes and improves neuropathy in CMT1A mice

**DOI:** 10.1172/JCI159814

**Published:** 2022-07-01

**Authors:** Marina Stavrou, Alexia Kagiava, Sarah G. Choudury, Matthew J. Jennings, Lindsay M. Wallace, Allison M. Fowler, Amanda Heslegrave, Jan Richter, Christina Tryfonos, Christina Christodoulou, Henrik Zetterberg, Rita Horvath, Scott Q. Harper, Kleopas A. Kleopa

**Affiliations:** 1 Neuroscience Department, The Cyprus Institute of Neurology and Genetics, Nicosia, Cyprus.; 2 Center for Gene Therapy, The Abigail Wexner Research Institute at Nationwide Children’s Hospital, Columbus, Ohio, USA.; 3 Department of Clinical Neurosciences, University of Cambridge, Cambridge, United Kingdom.; 4 Department of Neurodegenerative Disease, University College London (UCL) Institute of Neurology, London, United Kingdom.; 5 UK Dementia Research Institute at UCL, London, United Kingdom.; 6 Molecular Virology Laboratory, The Cyprus Institute of Neurology and Genetics, Nicosia, Cyprus.; 7 Department of Psychiatry and Neurochemistry, Institute of Neuroscience and Physiology, The Sahlgrenska Academy at the University of Gothenburg, Mölndal, Sweden.; 8 Clinical Neurochemistry Laboratory, Sahlgrenska University Hospital, Mölndal, Sweden.; 9 Hong Kong Center for Neurodegenerative Diseases, Hong Kong, China.; 10 Department of Pediatrics, The Ohio State University School of Medicine, Columbus, Ohio, USA.; 11 Center for Neuromuscular Disorders, The Cyprus Institute of Neurology and Genetics, Nicosia, Cyprus.

**Keywords:** Neuroscience, Therapeutics, Gene therapy, Mouse models, Neurodegeneration

## Abstract

Charcot-Marie-Tooth disease type 1A (CMT1A), the most common inherited demyelinating peripheral neuropathy, is caused by *PMP22* gene duplication. Overexpression of WT PMP22 in Schwann cells destabilizes the myelin sheath, leading to demyelination and ultimately to secondary axonal loss and disability. No treatments currently exist that modify the disease course. The most direct route to CMT1A therapy will involve reducing PMP22 to normal levels. To accomplish this, we developed a gene therapy strategy to reduce *PMP22* using artificial miRNAs targeting human *PMP22* and mouse *Pmp22* mRNAs. Our lead therapeutic miRNA, miR871, was packaged into an adeno-associated virus 9 (AAV9) vector and delivered by lumbar intrathecal injection into C61-het mice, a model of CMT1A. AAV9-miR871 efficiently transduced Schwann cells in C61-het peripheral nerves and reduced human and mouse PMP22 mRNA and protein levels. Treatment at early and late stages of the disease significantly improved multiple functional outcome measures and nerve conduction velocities. Furthermore, myelin pathology in lumbar roots and femoral motor nerves was ameliorated. The treated mice also showed reductions in circulating biomarkers of CMT1A. Taken together, our data demonstrate that AAV9-miR871–driven silencing of PMP22 rescues a CMT1A model and provides proof of principle for treating CMT1A using a translatable gene therapy approach.

## Introduction

Charcot-Marie-Tooth (CMT) disease includes a genetically heterogeneous group of inherited peripheral neuropathies with a prevalence of up to 1 in 2500 persons (1, 2). The autosomal-dominant demyelinating CMT neuropathy type 1A (CMT1A [Mendelian inheritance in man (MIM) 118220]) is the most common type, accounting for more than 50% of all CMT cases and resulting from an intrachromosomal duplication spanning 1.4 Mb on human chromosome 17p12 (3). The responsible disease gene (*PMP22*) encodes the peripheral myelin protein of 22 kDa (PMP22), which is located within this duplicated region (4–7). Patients with CMT1A develop distal muscle weakness and atrophy as well as sensory loss and have absent reflexes, with typical onset in adolescence. CMT1A is slowly progressive, with marked variability in disease severity (8, 9). Sensory responses are usually absent, whereas motor nerve conduction velocities (MNCVs) are slowed, ranging from 5 to 35 m/s in the forearm, but most average around 20 m/s, with uniform and symmetric findings in different nerves. Although NCVs do not change significantly over decades, motor amplitudes and the number of motor units decrease slowly, reflecting axonal loss and correlating with progressive clinical disability.

PMP22 is mainly expressed by myelinating Schwann cells (SCs) and localized in compact myelin (10) but is also present in non-neural cell types such as fibroblasts, endothelia, and epithelia (11). Mouse studies support the idea that PMP22 is normally involved in early steps of myelin formation and in the maintenance of myelin and axons in the PNS (12–15). In humans, *PMP22* mRNA and PMP22 protein overexpression in nerve biopsies form patients with CMT1A indicates that increased PMP22 dosage is the most likely disease mechanism underlying CMT1A (16–19). This hypothesis is further supported by a recapitulation of numerous CMT1A-associated phenotypes in PMP22-overexpressing rodent models (20–30), including C61-het mice (22), which contain 4 copies of WT human *PMP22* on a normal mouse background. The exact mechanisms by which PMP22 overexpression causes CMT1A remain unclear but may involve proteasome dysfunction related to excessive amounts of PMP22 protein. Specifically, in normal myelinating and nonmyelinating SCs, approximately 20% of newly synthesized PMP22 is glycosylated, while the remaining approximately 80% is targeted for proteasomal endoplasmic reticulum–associated degradation (ERAD) (31). Thus, in CMT1A, overexpressed PMP22 is thought to accumulate in perinuclear aggresomes (32, 33) and impair overall proteasome activity (34), resulting in myelin sheath destabilization in SCs and ultimately nerve dysfunction.

Based on this model, the most direct approach to CMT1A therapy will likely involve reducing overexpressed PMP22 to normal levels in SCs. Prior attempts to accomplish this using drug-based approaches were unsuccessful in human clinical trials (NCT00484510, NCT02600286, NCT05092841, NCT04762758, NCT03023540), and to date, CMT1A remains intractable. Nevertheless, progress continues in the field, with prospective adjunct therapies approaching clinical trials (35–37) and several preclinical strategies to silence PMP22 reported (38–61). For example, CRISPR/Cas9 was used to directly target the *PMP22* gene by deleting its regulatory regions with encouraging results in vitro (62) and in vivo (63), and oligonucleotides have been tested to inhibit *PMP22* via promoter disruption or through mRNA degradation using RNAase H or RNAi-based mechanisms (using DNA gapmers or siRNAs, respectively) in different CMT1A rodent models (64–67). Among these various silencing approaches, RNAi has so far been used most often as a prospective mechanism to develop a CMT1A therapy (68).

RNAi is a conserved process of gene silencing triggered by endogenous miRNAs, which are encoded in the genomes of eukaryotic organisms. Mature forms of natural miRNAs are small (approximately 22 nucleotides long), noncoding RNA molecules that negatively regulate the expression of a vast fraction of the transcriptome at the posttranscriptional level (69, 70). Importantly, natural miRNAs can be modified in the form of siRNAs, shRNAs, or artificial miRNAs and retargeted to specifically base pair with disease genes, triggering target mRNA degradation through the RNA-induced silencing complex (RISC). siRNAs are chemically synthesized, produce transient effects, and require repeated, lifelong administration to achieve long-term gene silencing. In contrast, shRNAs or artificial miRNAs can be cloned as DNA expression cassettes, delivered to target cells within viral vectors, and transcribed in vivo to produce long-term target gene silencing after 1 administration. As mentioned, siRNAs have been used to trigger RNAi against *PMP22* in at least 3 published studies (65, 67), but RNAi treatment of CMT1A in in vivo and in vitro models was also achieved using gene therapy vectors expressing natural miRNAs (mir-29a; ref. 71 or mir-318; ref. 72) or by an intraneurally injected AAV2/9 vector expressing rodent *Pmp22*-targeting artificial shRNAs (73). These shRNAs contained mismatches with the human *PMP22* sequence and were not tested in models expressing human *PMP22*, so it is unclear if these sequences, as well as the invasive and laborious intraneural injection method, can be translated to humans (73).

Here, we designed and tested a translatable AAV9-based gene therapy approach for CMT1A using an artificial miRNA targeting conserved regions on the human *PMP22* and mouse *Pmp22* transcripts. We demonstrate long-lasting therapeutic effects following a single, clinically relevant lumbar intrathecal injection in a mouse model of CMT1A that expressed both human *PMP22* and mouse *Pmp22* gene products. Thus, our study provides proof of principle for treating CMT1A with a gene therapy approach that uses artificial miRNA sequences and a route of administration that can be translated to human trials.

## Results

### Design and in vitro validation of artificial miRNAs to downregulate human and murine PMP22.

**Figure 1:**
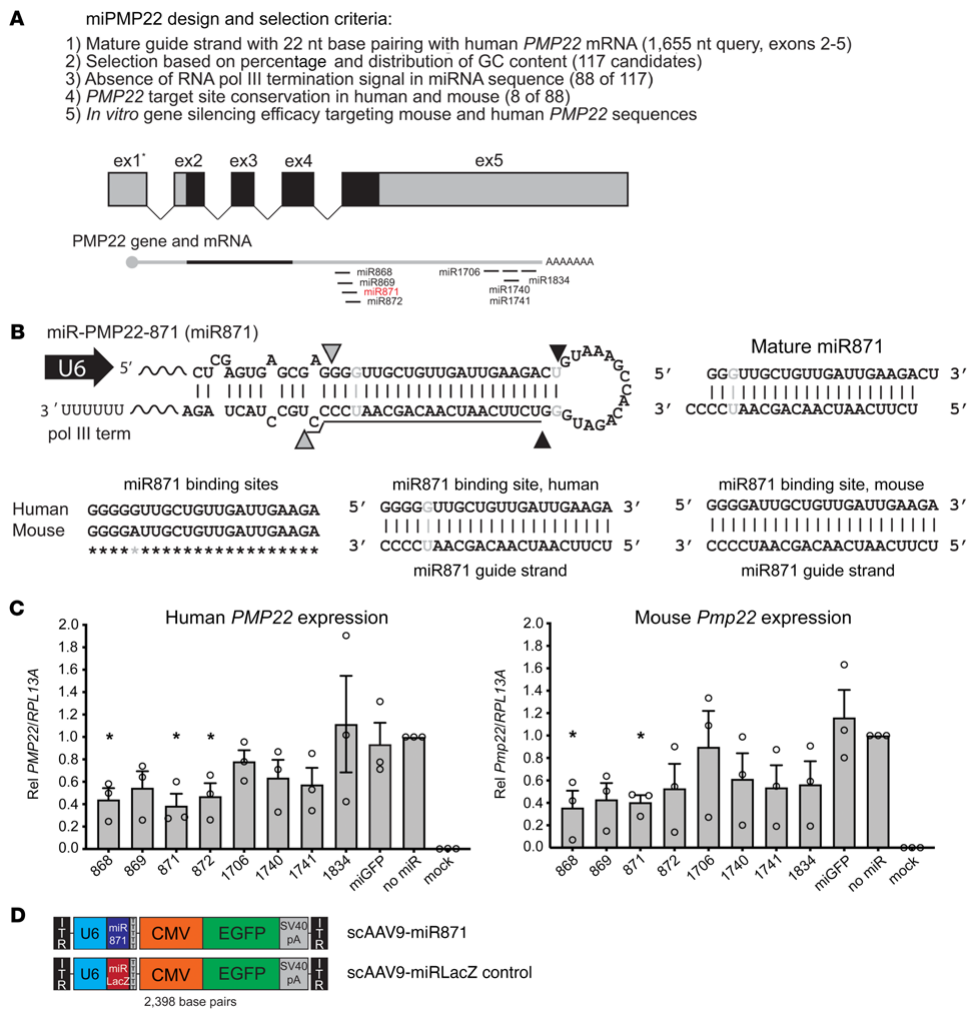
Design and in vitro screen of artificial miRNAs targeting PMP22. (**A**) Full-length *PMP22* mRNA was transcribed from 5 exons (ex), producing 2 major transcripts with identical ORFs (black shading). We designed 8 candidate miRNAs to equally target both human *PMP22* and murine *Pmp22* mRNAs. (**B**) Gray and black arrowheads show miR871 cut sites by Drosha and Dicer, respectively. dsRNAs form G:U wobble base pairs (indicated by gray shading). Underlined sequence represents the mature miR871 antisense guide strand. Bottom panel shows alignment of the miR871 binding site on human *PMP22* and murine *Pmp22* mRNAs. Gray asterisk indicates a G:A mismatch at the miR871 binding site, but each nucleotide at this location can form 2 hydrogen bonds with the miR871 guide strand as a G:U wobble (human) or A:U wobble (mouse). (**C**) RT-qPCR was performed to measure in vitro human *PMP22* or murine *Pmp22* silencing by the indicated miRPMP22s (*n =* 3/group). Gene expression was normalized to human *RPL13A*. *P < 0.05, by unpaired, 1-tailed Student’s *t* test. Values represent the mean ± SEM. Rel, relative. (**D**) Schematic of scAAV9, which was used to deliver miR871 or miRLacZ expression cassettes in vivo. The U6 promoter drives the transcription of miR871 or miRLacZ, and the CMV promoter drives the *EGFP* gene with the SV40 polyadenylation sequence.

Full-length human *PMP22* and mouse *Pmp22* are encoded by 5 exons, with 2 alternatively spliced first exons containing 5′-UTR sequences (ex1* 1a and ex1b). Both variants encode identical 483 bp ORFs and share the same 3′-UTR, which is located in exon 5 (ex5) (ORFs) (Figure 1A). To ensure that we targeted all *PMP22* transcripts, we excluded exon 1 from the query sequence and designed artificial miRNAs targeting human *PMP22* exons 2 to 5 (1655 nucleotides) using a previously described algorithm (74). This screen identified 117 candidates. Because we intended to use the RNA polymerase III–dependent (pol III–dependent) U6 promoter to drive miRNA expression, we excluded 29 of the 117 candidates because of the presence of RNA pol III termination sequences (5–6 T’s) within the miRNA expression cassettes. The remaining 88 sequences were additionally filtered to ensure that the antisense guide strand of the miRPMP22 miRNAs would equally target human *PMP22* and mouse *Pmp22* sequences. Only 8 sequences (9%) showed this conservation, and all were located in exon 5, which encodes the 3′-UTR (Figure 1A and Supplemental Figure 1; supplemental material available online with this article; https://doi.org/10.1172/JCI159814DS1). Following cloning into a U6T6 expression plasmid, we empirically tested all 8 miRPMP22 miRNAs (miR868, miR869, miR871, miR872, miR1706, miR1740, miR1741, and miR1834) for silencing efficacy (Figure 1, B and C). Specifically, we cotransfected HEK293 cells with each individual U6-miRPMP22 plasmid and CMV-driven *PMP22* or *Pmp22* full-length cDNAs, and then harvested RNA 24 hours later, generated cDNA, and performed real-time quantitative PCR (RT-qPCR) using for *PMP22* or *Pmp22*, normalized to *RPL13A*. Negative controls included cells transfected with *PMP22* or *Pmp22* and U6.miRGFP (miRNA targeting EGFP) or an empty U6T6 plasmid (no miR). Data were collected and averaged from 3 independent experiments, with each RT-qPCR assay performed in triplicate. Although 7 of 8 miRPMP22s (87.5%) showed some level of silencing compared with the “no miR” control, only miR868 and miR871 showed statistically significant (*P* < 0.05) silencing of *PMP22* and *Pmp22* sequences. Because miR871 consistently silenced both genes by approximately 60%, we chose the miR871 sequence as our lead. The U6-miR871 sequence was then subcloned into a self-complementary adeno-associated virus (AAV) (scAAV.CMV.EGFP) backbone containing a separate CMV.EGFP reporter gene (Figure 1D), and we generated AAV9 particles using triple transfection in HEK293 cells (hereafter referred to as AAV9-miR871). Lysates were purified by iodixanol-gradient ultracentrifugation and fast protein liquid chromatography (FPLC), as previously described (75). Similarly, we generated a control scAAV9.CMV.EGFP vector expressing a U6 promoter–driven miRNA targeting the *E*. *coli* *LacZ* gene (hereafter referred to as AAV9-miRLacZ).

### Biodistribution and expression following lumbar intrathecal injection of AAV9-miR871.

**Figure 2:**
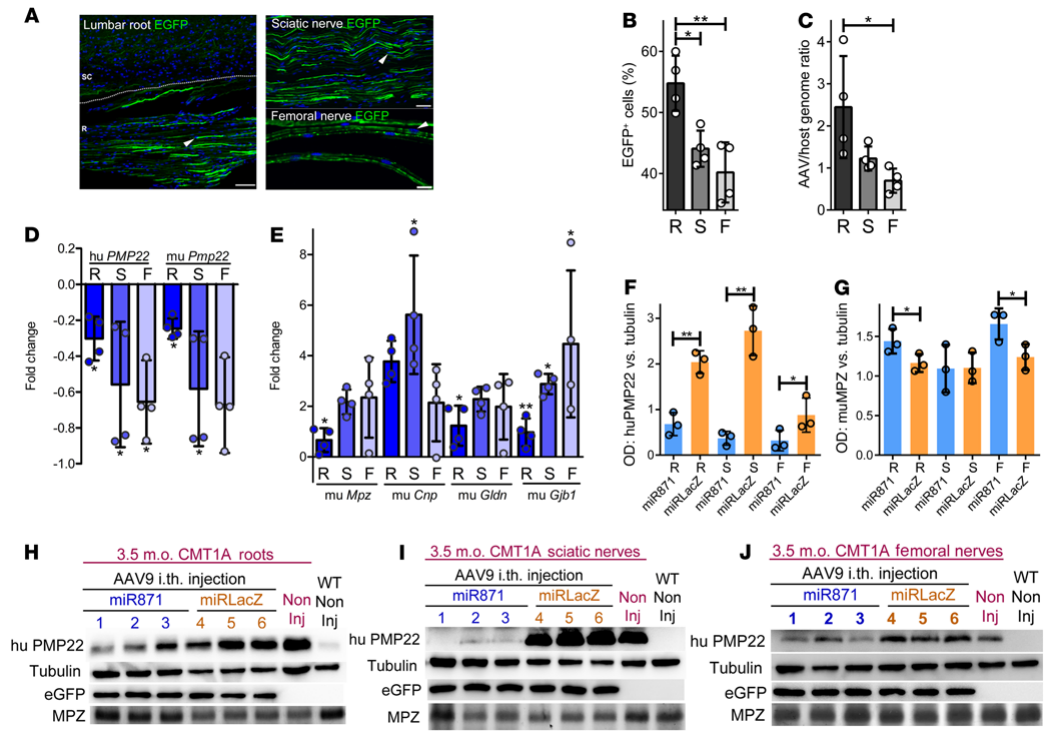
In vivo assessment of AAV9-miR transduction in PNS tissues and validation of AAV9-miR871 silencing efficiency in a CMT1A mouse model 6 weeks after injection. (**A**) Lumbar spinal roots and sciatic nerve sections as well as teased femoral nerve fibers showing EGFP autofluorescence in SCs and axons. Arrowheads indicate examples of EGFP^+^ nuclei. Scale bars: 60 μm (lumbar root and sciatic nerve) and 20 μm (femoral nerve). (**B**) Quantification of EGFP-expressing PNS cells (*n =* 4/group). (**C**) VGCNs (*n =* 4/group) confirmed peripheral nerve transduction. RT-qPCR analysis of (**D**) Human (hu) *PMP22* and murine (mu) *Pmp22* and of murine (**E**) *Mpz*, *Cnp*, *Gldn*, and *Gjb1* gene expression (*n =* 3/group). Fold changes in relative mRNA expression of CMT1A-AAV9-miR871 were calculated in comparison with expression levels in CMT1A-AAV9-miRLacZ mice. All samples were normalized to endogenous *Gapdh*. Quantification of (**F**) human PMP22 and (**G**) murine MPZ Western blot protein ODs, normalized to tubulin (Tub), in CMT1A-AAV9-miR871 and CMT1A-AAV9-miRLacZ mice in lumbar roots, sciatic nerves, and femoral nerves. Western blots showing human PMP22, murine tubulin, EGFP, and murine MPZ protein levels in (**H**) roots, (**I**) sciatic nerves, and (**J**) femoral nerves. Values represent the mean ± SD. *P < 0.05 and **P < 0.01, by 1-way ANOVA with Tukey’s multiple-comparison test. R, lumbar roots; S, sciatic nerves; F, femoral nerves; i.th., intrathecal; m.o., months old; Non Inj, noninjected.

We delivered the AAV9-miR871 vector expressing the EGFP gene under the CMV promoter into 2-month-old C61 heterozygous mice (22) (hereafter referred to as the CMT1A mouse) using lumbar intrathecal injection (20 μL containing a total of 5e11vg/mouse). Six weeks after injection, we examined AAV9-miR871 biodistribution and transduction in PNS cells. For this purpose, we used vector genome copy numbers (VGCNs) and EGFP expression analysis in anterior lumbar roots and sciatic and femoral nerves. EGFP was detected as autofluorescence in the perinuclear cytoplasm of a subset of PNS cells as well as in the axons of lumbar roots and sciatic and femoral nerves (Figure 2A). The percentage of EGFP-expressing SCs in immunostained tissue sections reached an average of 54.78% ± 4.53% in anterior lumbar roots, 44.07% ± 2.96% in sciatic nerves, and 40.18% ± 4.93% in femoral nerves (*n =* 4 mice; Figure 2B). VGCNs in DNA extracted from PNS tissues reached 2.44 in anterior lumbar roots, 1.23 in sciatic nerves, and 0.69 in femoral nerves (*n =* 4 mice; Figure 2C).

### In vivo validation of AAV9-miR871–mediated silencing of the PMP22 gene in CMT1A mice.

Prior to any treatment studies, we performed a detailed characterization of baseline functional and morphological deficits of the C61-het CMT1A mouse line, which contains 4 copies of the human *PMP22* gene and 2 normal copies of murine *Pmp22*, compared with WT mice at 2, 4, 6, 8, and 10 months of age. We confirmed progressive functional impairment associated with early-onset demyelination (Supplemental Figures 2–7). We also assessed the potential toxicity of the AAV9-miRLacZ vector after injection into 2-months-old CMT1A mice that were examined 6 weeks (3.5 months of age) or 4 months (6 months of age) later. AAV9-miRLacZ caused no significant increase in the numbers of inflammatory cells in spinal roots, sciatic nerves, or dorsal root ganglia (DRGs) beyond the baseline (Supplemental Figures 8, 9, and 11). However, injection of AAV9-miRLacZ increased the number of CD20^+^ and CD3^+^ cells in CMT1A mouse livers 6 weeks after injection (3.5 months of age), but this reaction subsided by the 4-month post-injection time point (6 months of age; Supplemental Figure 10). Interestingly, inflammatory infiltrates increased with age in the PNS of noninjected CMT1A mice (Supplemental Figures 8 and 9).

After we confirmed sufficient biodistribution, transduction of PNS tissues, and safety, we evaluated the efficacy of AAV9-miR871 in silencing *PMP22/Pmp22* gene expression and reducing overall human and mouse PMP22 protein levels, compared with the expression of other myelin-related genes and proteins. We injected AAV9-miR871, which targets both the *PMP22* and *Pmp22* transcripts, or the AAV9-miRLacZ negative control, which expresses a functional but nontargeting miRNA, into adult CMT1A mice and then analyzed gene expression by RT-qPCR and Western blotting 6 weeks after injection. At the mRNA level, AAV9-miR871 downregulated *PMP22* and *Pmp22* in spinal roots and sciatic and femoral nerves, whereas other myelin-related genes were mostly elevated (Figure 2, D and E, and Supplemental Table 1). *Gjb1* transcript levels were increased in all tissues examined. *Mpz* and *Gldn* transcript levels were elevated only in roots, whereas *Cnp* transcript levels were elevated only in sciatic nerves. At the protein level (Figure 2, F–J), AAV9-miR871 selectively reduced human PMP22 levels in all PNS tissues examined (in roots: –66%, sciatic nerve: –86%, femoral nerve: –64%), whereas MPZ protein levels were increased in roots (by 23%) and femoral nerves (by 34%).

### Early treatment of CMT1A mice.

**Figure 3:**
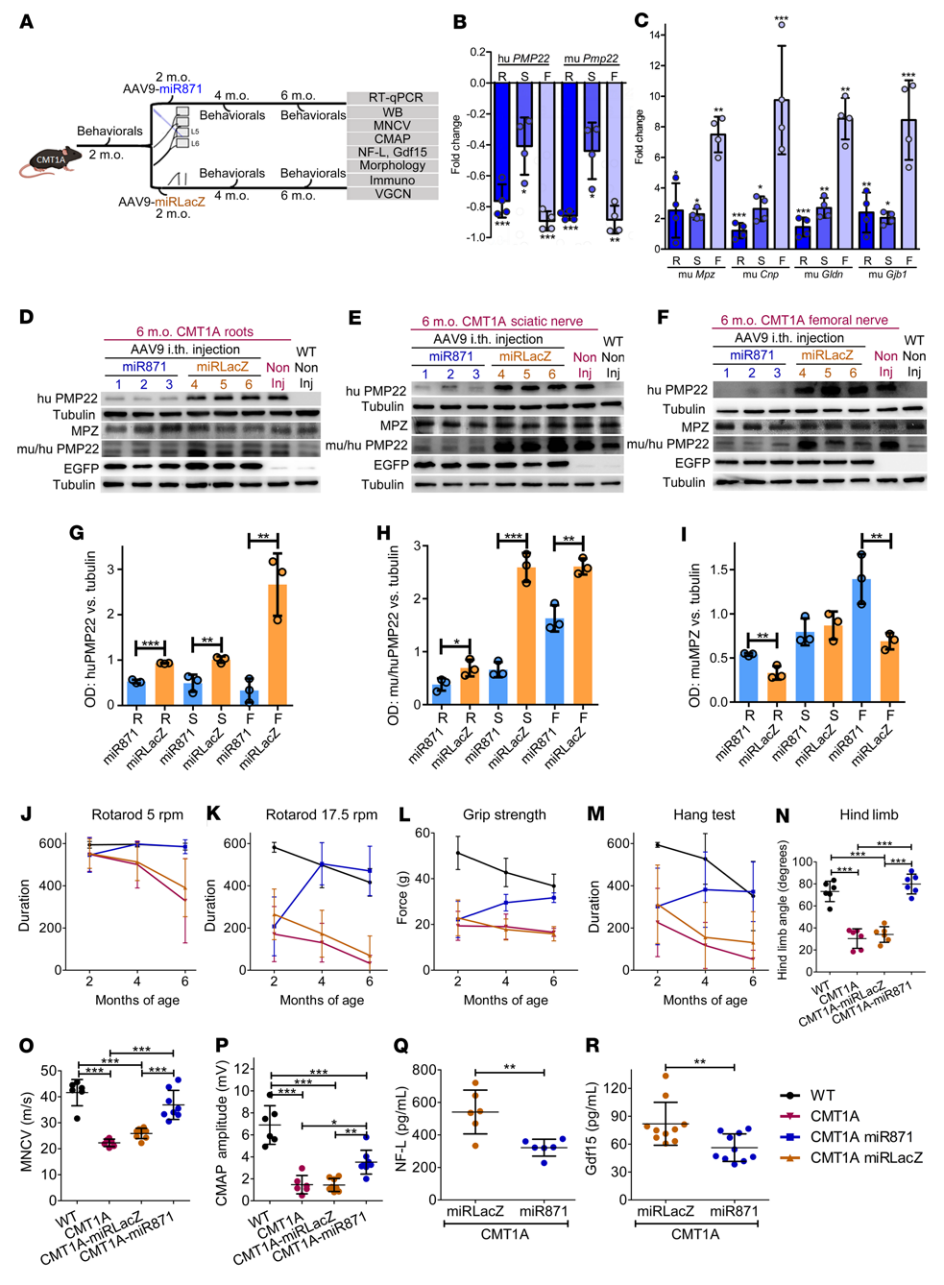
Efficient *PMP22/Pmp22* silencing and improvement of motor behavioral, electrophysiological, and blood biomarker phenotypes following early treatment of CMT1A mice. (**A**) Design of the early treatment trial. RT-qPCR analysis of (**B**) human *PMP22* and murine *Pmp22* and murine (**C**) *Mpz*, *Cnp*, *Gldn*, and *Gjb1* (**C)** gene expression levels in lumbar roots, sciatic nerves, and femoral nerves (*n =* 4/group). (**D**–**I**) Western blot images and analysis of human PMP22, murine PMP22, murine tubulin, EGFP, and murine MPZ proteins levels. (**J**–**M**) Behavioral analysis comparing noninjected WT and CMT1A mice (*n =* 10/group) and CMT1A-AAV9-miR871 and CMT1A-AAV9-miRLacZ mice (*n =* 16/group). (**N**) Hind limb opening angle estimation for 6-month-old noninjected WT and CMT1A mice (*n =* 6/group) as well as for CMT1A-AAV9-miR871 and CMT1A-AAV9-miRLacZ mice (*n =* 6/group). (**O**) MNCV and (**P**) CMAP analysis of 6-month-old WT and noninjected CMT1A mice (*n =* 6/group) and CMT1A-AAV9-miR871 and CMT1A-AAV9-miRLacZ mice (*n =* 8/group). (**Q**) NF-L (*n =* 6/group) and (**R**) Gdf15 (*n =* 10/group) circulating biomarker analysis of 6-month-old CMT1A-AAV9-miR871 and CMT1A-AAV9-miRLacZ mice. Values represent the mean ± SD. *P < 0.05, **P < 0.01, and ***P < 0.001, by unpaired, 1-tailed Student’s *t* test (**B**, **C**, **Q**, and **R**) and 1-way ANOVA with Tukey’s multiple-comparison test (**G**–**P**). For **J**–**M**, statistical significance is shown in Supplemental Figure 13.

**Figure 4:**
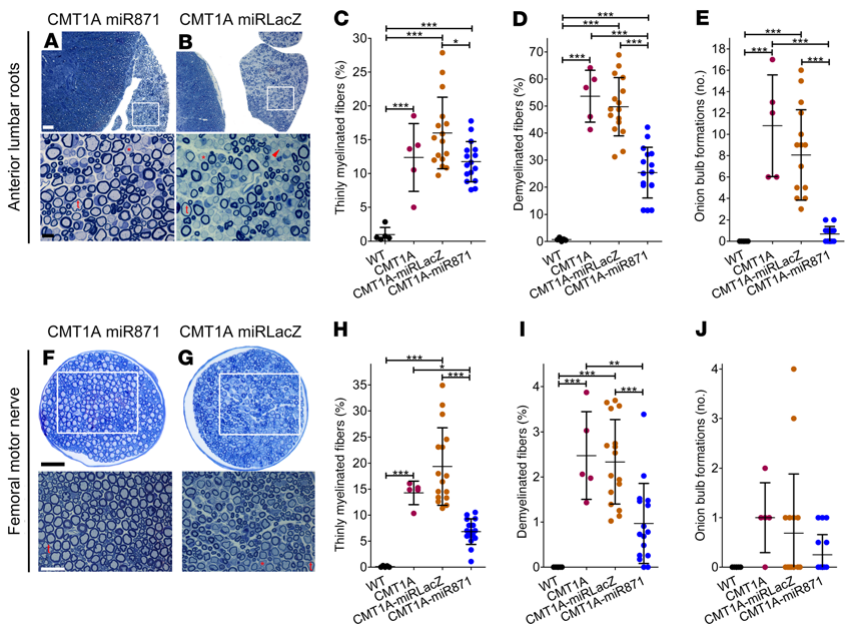
Early treatment of CMT1A mice improved PNS tissue morphology. Toluidine blue–stained semithin sections of (**A** and **B**) anterior lumbar spinal roots attached to the spinal cord and (**F** and **G**) femoral motor nerve at low (upper panels) and higher (lower panels) magnification from CMT1A-AAV9-miR871 and CMT1A-AAV9-miRLacZ mice. Thinly myelinated (t) or demyelinated (red asterisk) fibers as well as onion bulb formations (red arrowhead) are indicated. Quantification of abnormally myelinated fibers in (**C**–**E**) lumbar motor roots and (**H**–**J**) femoral motor nerves from 6-month-old noninjected WT and CMT1A mice (*n =* 5/group), as well as from CMT1A-AAV9-miR871 and CMT1A-AAV9-miRLacZ (*n =* 16/group). Values represent the mean ± SD. *P < 0.05, **P < 0.01, and ***P < 0.001, by 1-way ANOVA with Tukey’s multiple-comparison test. Scale bars: (**A** and **B**) 50 μm and 10 μm (enlarged insets); (**F** and **G**) 40 μm and 25 μm (enlarged insets).

**Figure 5:**
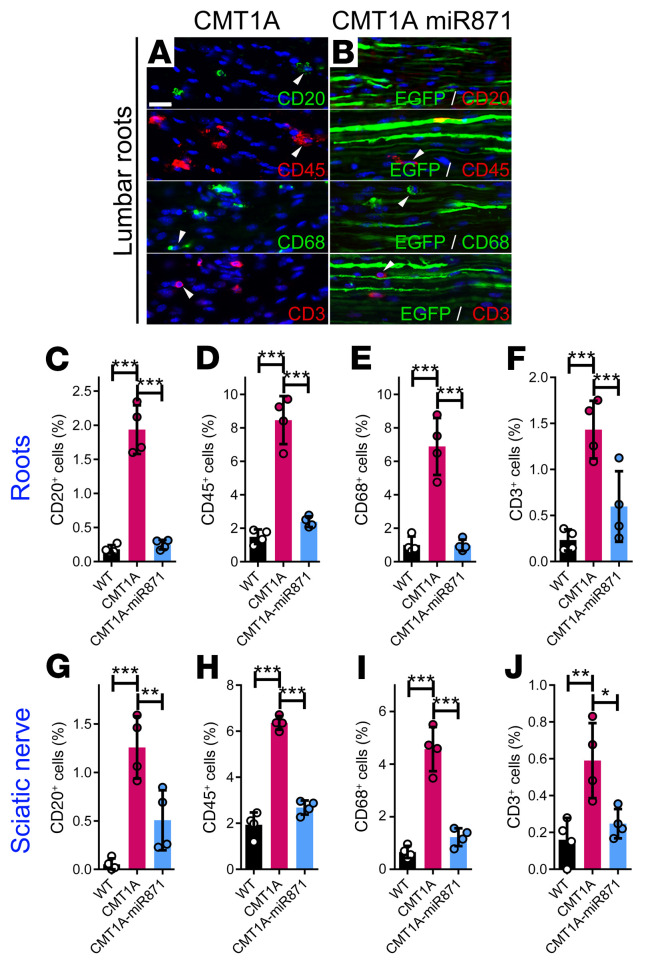
Early treatment of CMT1A mice improved inflammation in PNS tissues. Images of longitudinal lumbar spinal root sections from noninjected CMT1A mice and mice that received early treatment with CMT1A-AAV9-miR871. Root sections were immunostained for CD20, CD45, CD68, and CD3 markers (**A** and **B**). The injected tissues were counterstained with the nuclear marker DAPI (blue) and EGFP (green) autofluorescence. Arrowheads indicate representative CD^+^ cells. Quantification of the percentage of inflammatory cells in lumbar roots (**C**–**F**) and sciatic nerve (**G**–**J**). Values represent the mean ± SD (*n =* 4/group). *P < 0.05, **P < 0.01, and ***P < 0.001, by 1-way ANOVA with Tukey’s multiple-comparison test followed by Bonferroni’s correction. Scale bar: 20 μm. (WT and CMT1A immunostained images and quantification data are also shown in Supplemental Figures 8 and 9.)

After validating AAV9-miR871 in vivo PNS biodistribution and *PMP22* and *Pmp22* gene silencing efficacy, we proceeded with a proof-of-concept treatment trial at early stages of neuropathy in the CMT1A mouse model. Two-month-old CMT1A mice were injected with either AAV9-miR871 or AAV9-miRLacZ and evaluated 4 months after injection. For outcome analysis, we included *PMP22* and *Pmp22* expression levels using real-time PCR and Western blotting, behavioral testing, circulating neurofilament light (NF-L) and growth differentiation factor 15 (Gdf15) quantification, electrophysiological examination, as well as morphometric analysis of myelination in semithin sections and evaluation of inflammatory infiltrates in the PNS by IHC (Figures 3–5). We confirmed adequate biodistribution by VGCN measurement in PNS and non-PNS tissues as well as by immunofluorescence analysis in lumbar roots and sciatic nerves (Supplemental Figure 12).

At the mRNA level, early treatment with AAV9-miR871 in CMT1A mice (Figure 3A) downregulated *PMP22* and *Pmp22* in roots and sciatic and femoral nerves, while also elevating *Mpz, Cnp, Gldn*, and *Gjb1* transcripts levels (Figure 3, B and C, and Supplemental Table 2). At the protein level, early treatment with AAV9-miR871 in CMT1A mice reduced human and murine PMP22 levels in spinal roots (–43% human PMP22; –45% murine PMP22), sciatic nerves (–51% human PMP22; –74% murine PMP22), and femoral nerves (–87% human PMP22; –38% murine PMP22) (Figure 3, D–I). In contrast, we found that murine MPZ protein levels were increased in roots (63%) and femoral nerves (102%), reflecting improved myelination, whereas they remained unchanged in sciatic nerves (Figure 3, D–I).

We assessed motor performance in all groups before injection and until the end of the observation period by rotarod (5 and 17.5 rpm), grip, and hang tests (Figure 3, J–M, and Supplemental Figure 13). Time-course analysis of the above tests showed that AAV9-miR871 treatment improved the motor performance of CMT1A mice, reaching WT levels, whereas AAV9-miRLacZ–treated CMT1A mice performed similarly to noninjected CMT1A mice and significantly worse than WT mice (Figure 3, J–M, and Supplemental Figure 13). Moreover, early treatment with AAV9-miR871 completely rescued the hind limb clasping phenotype of CMT1A mice (Figure 3N and Supplemental Figure 14).

Electrophysiological examination in 6-month-old mice (4 months after vector injection) (Figure 3, O and P) showed a significantly improved MNCV score for AAV9-miR871–treated mice (36.87 ± 5.60 m/s) compared with that of the AAV9-miRLacZ–treated group (25.89 ± 1.99 m/s), approaching WT values at the same age (41.61 ± 5.06 m/s). Although the amplitude of the compound muscle action potential (CMAP) was also significantly improved in the treated mice (3.52 ± 1.08 mV) compared with AAV9-miRLacZ–treated controls (1.44 ± 0.59 mV), it did not reach WT levels (6.89 ± 1.76 mV).

As with other CMT blood biomarker studies, we found that circulating NF-L (76–79) and Gdf15 (80, 81) levels, associated with axonal degeneration, were significantly ameliorated after early treatment of CMT1A mice with AAV9-miR871 (NF-L: 321.37 ± 51.68 pg/mL; Gdf15: 56.25 ± 14.84 pg/mL) compared with their AAV9-miRLacZ vector–treated littermates (540.65 ± 134.49 pg/mL; Gdf15: 81.93 ± 23.12 pg/mL) (Figure 3, Q and R). This reduction in NF-L and Gdf15 levels in the AAV9-miR871 treatment group is consistent with improved motor function following gene-silencing treatment. Thus, NF-L and Gdf15 blood levels may be useful as treatment-responsive and clinically relevant biomarkers for future gene therapy in patients with CMT1A.

We performed morphometric analysis of myelination in transverse semithin sections of anterior lumbar roots and femoral motor nerves of 6-month-old CMT1A mice injected at the age of 2 months with either the AAV9-miR871 or the AAV9-miRLacZ vector. We examined multiple roots and bilateral femoral motor nerves from each mouse and calculated the percentage of thinly myelinated and demyelinated fibers, as well as the number of onion bulb formations. In both roots (Figure 4, A–E) and femoral nerves (Figure 4, F–J), the percentage of thinly myelinated and demyelinated fibers was significantly reduced in the treated mice. Spinal roots also showed reduced numbers of onion bulb formations, whereas femoral onion bulb formations were already low at baseline and not altered after treatment. The degree of myelin pathology was too mild in the sciatic nerves of CMT1A mice to be considered as a treatment readout (Supplemental Figure 15).

Finally, for this early treatment group, we used immunofluorescence analysis to evaluate the inflammatory status of lumbar roots and sciatic nerves (Figure 5 and Supplemental Figures 16–18). AAV9-miR871 treatment decreased the percentage of CD20^+^, CD45^+^,, CD68^+^,, and CD3^+^ cells. Moreover, injection with the therapeutic vector did not cause any inflammatory responses in the liver at the 4-month post-injection point (Supplemental Figure 18).

### Late treatment compared with extended early treatment.

**Figure 6:**
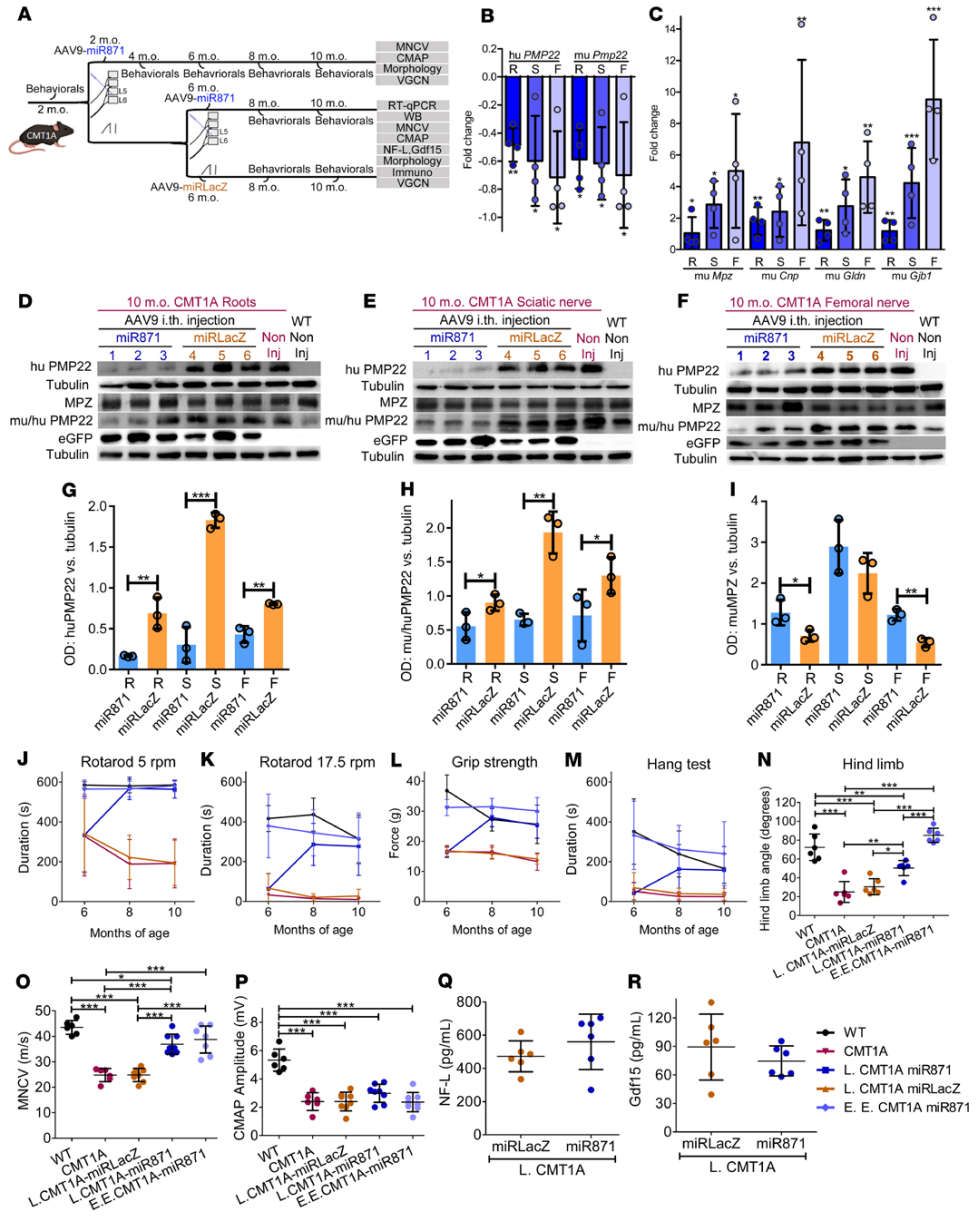
Efficient *PMP22/Pmp22* silencing and improvement of motor function and sciatic MNCVs but not CMAPs or blood biomarker phenotypes following late treatment of CMT1A mice. (**A**) Design of the late (L.) and extended early (E.E.) treatment trial. RT-qPCR analysis of (**B**) *PMP22* and *Pmp22* and (**C**) *Mpz*, *Cnp*, *Gldn*, and *Gjb1* gene expression in lumbar roots and sciatic and femoral nerves of late-treated CMT1A mice (*n =* 4/group). (**D**–**I**) Western blots and analysis of human PMP22, murine PMP22, murine tubulin, EGFP, and murine MPZ protein levels. (**J**–**M**) Behavioral analysis comparing noninjected WT and CMT1A mice (*n =* 10/group) and CMT1A-AAV9-miR871 and CMT1A-AAV9-miRLacZ mice (*n =* 16/group). (**N**) Hind limb opening angle estimation for 10-month-old noninjected WT and CMT1A mice (*n =* 6/group) and L.CMT1A-AAV9-miR871, E.E.CMT1A-AAV9-miR871, and CMT1A-AAV9-miRLacZ mice (*n =* 6/group). (**O**) MNCV and (**P**) CMAP analysis of 10-month-old WT and noninjected CMT1A mice (*n =* 6/group) and L.CMT1A-AAV9-miR871, E.E.CMT1A-AAV9-miR871, and CMT1A-AAV9-miRLacZ mice (*n =* 8/group). (**Q**) NF-L (*n =* 6/group) and (**R**) Gdf15 (*n =* 10/group) circulating biomarker analysis in 10-month-old L.CMT1A-AAV9-miR871 and L.CMT1A-AAV9-miRLacZ mice. Values represent the mean ± SD. *P < 0.05, **P < 0.01, and ***P < 0.001, by unpaired, 1-tailed Student’s *t* test (**B**, **C**, **Q**, and **R**) and 1-way ANOVA with Tukey’s multiple-comparison test (**G**–**P**). For **J**–**M**, statistical significance is shown in Supplemental Figures 21 and 22.

After assessing the effectiveness of early treatment with AAV9-miR871 in CMT1A mice, we further examined its effectiveness when injected later in the disease course. We injected mice either at 6 months (late treatment) or 2 months (extended early treatment) of age and analyzed various outcomes at 10 months of age. We evaluated both mice that received late treatment (4 months after injection) and mice that were had extended early treatment (8 months after injection) using a VGCN calculation, behavioral testing, blood NF-L and Gdf15 testing, electrophysiological examination, as well as by morphometric analysis of myelination and IHC, while real-time PCR and Western blot analysis were performed only in the late treatment groups (Figure 6A). Vector biodistribution in older animals was confirmed by VGCN in PNS and non-PNS tissues (Supplemental Figures 19A and 20). In the late treatment group, we confirmed vector biodistribution with EGFP expression levels in lumbar roots and sciatic nerves (Supplemental Figure 19B).

At the mRNA level, as with early treatment, late treatment with AAV9-miR871 in CMT1A mice downregulated *PMP22* and *Pmp22*, while also elevating *Mpz, Cnp, Gldn*, and *Gjb1* transcripts levels in roots and sciatic and femoral nerves (Figure 6, B and C, and Supplemental Table 3). At the protein level, late treatment with AAV9-miR871 reduced human and murine PMP22 levels in all PNS tissue samples examined (Figure 6, D–I). In contrast, murine MPZ protein levels were increased in roots and femoral nerves, reflecting improved myelination, but remained unchanged in sciatic nerves (Figure 6, D–I).

We compared motor performance of mice in the late treatment group with that of mice in the extended early treatment group and of age-matched, noninjected WT and CMT1A mice (Figure 6, J–N, and Supplemental Figures 21 and 22). We evaluated all groups before injection and until the end of the observation period by rotarod (5 rpm and 17.5 rpm), grip strength, and hang test analyses (Figure 6, J–M). Time-course analysis of the above tests showed that CMT1A mice that received late or extended early AAV9-miR871 treatment performed similarly, reaching WT levels, whereas CMT1A mice that received AAV9-miRLacZ late treatment performed similarly to noninjected CMT1A mice and significantly worse than WT mice (Figure 6, J–M, and Supplemental Figures 21 and 22). AAV9-miR871 late treatment improved the hind limb clasping phenotype of CMT1A mice, but without reaching WT levels, in contrast to mice given the extended early treatment, in which the phenotype was completely rescued, reaching WT levels (Figure 6N and Supplemental Figure 23).

Electrophysiological examination of 10-month-old mice showed that the sciatic MNCV was significantly improved in CMT1A mice in both the late (36.92 ± 3.94 m/s) and extended early (38.74 ± 5.30 m/s) AAV9-miR871 treatment groups compared with the AAV9-miRLacZ–treated group (24.82 ± 2.58 m/s) (Figure 6O). Interestingly, only the CMT1A mice that received extended early treatment reached age-matched WT values (43.50 ± 2.72 m/s). CMAP amplitudes were not improved in any of the AAV9-miR871 treatment groups (Figure 6P). Similarly, NF-L and Gdf15 levels remained elevated in the animals that were treated late compared with age-matched WT mice (Figure 6, Q and R).

**Figure 7:**
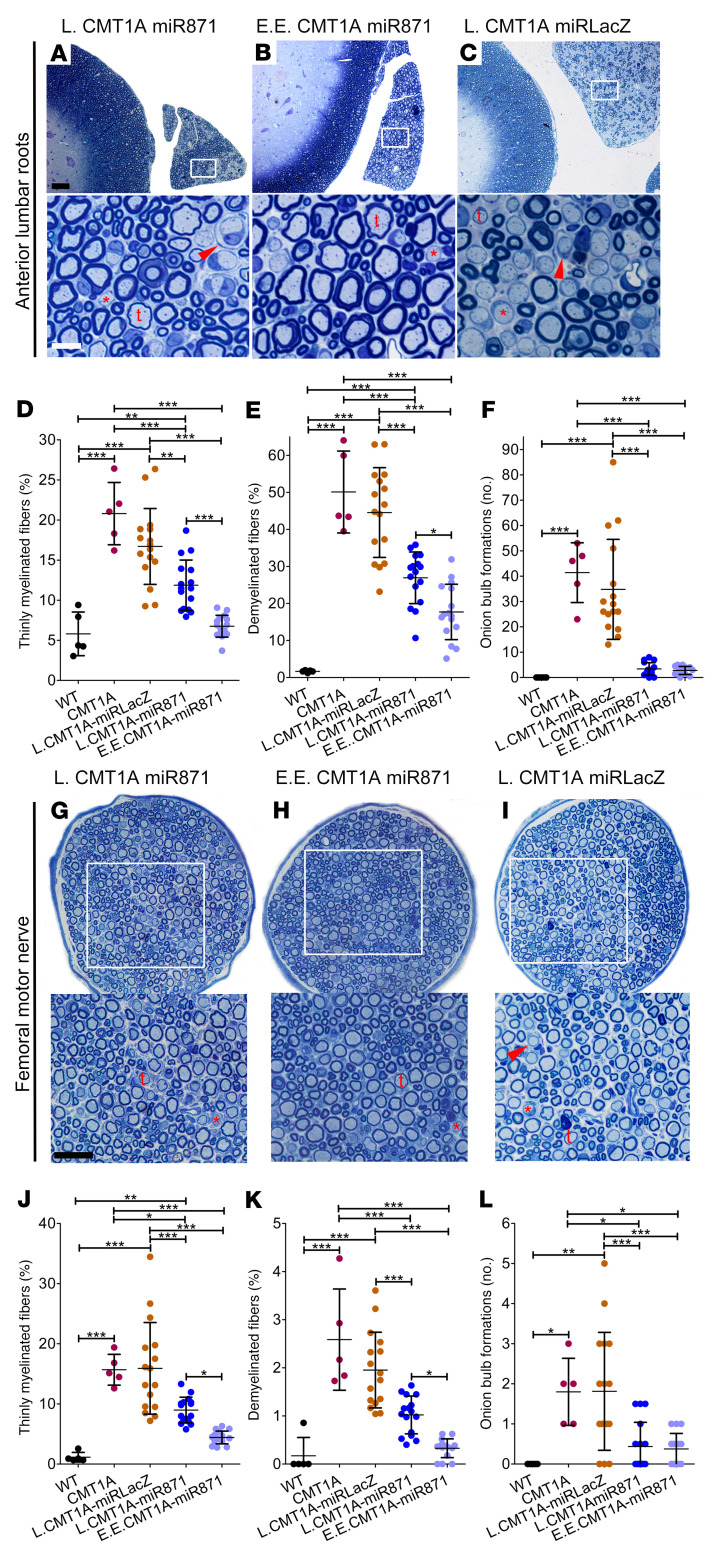
Late and extended early treatment of CMT1A mice improved PNS tissue morphology. Toluidine blue–stained semithin sections of (**A**–**C**) anterior lumbar spinal roots attached to the spinal cord and (**G**–**I**) femoral motor nerves (at low and higher magnification) from 10-month-old L.CMT1A-AAV9-miR871, E.E.CMT1A-AAV9-miR871, and L.CMT1A-AAV9-miRLacZ mice. Thinly myelinated (t) and demyelinated (red asterisk) fibers as well as onion bulb formations (red arrowhead) are indicated. **(D**–**F**) Quantification of abnormally myelinated fibers and onion bulb formations in multiple roots, and (**J**–**L**) femoral motor nerves from 10-month-old WT and noninjected CMT1A (*n =* 5/group) mice and L.CMT1A-AAV9-miR871, E.E.CMT1A-AAV9-miR871, and L.CMT1A-AAV9-miRLacZ mice (*n =* 16/group). Values represent the mean ± SD. *P < 0.05, **P < 0.01, and ***P < 0.001, by 1-way ANOVA with Tukey’s multiple-comparison test. Scale bars: (**A**–**C**) 50 μm and 10 μm (enlarged insets); (**G**–**I**) 40 μm and 25 μm (enlarged insets).

We performed morphometric analysis of myelination in transverse semithin sections of PNS tissues from 10-month-old CMT1A mice that received late or extended early treatment. With this analysis, we showed that the anterior lumbar roots (Figure 7, A–F) and femoral motor nerves (Figure 7, G–L) had significantly reduced percentages of thinly myelinated and demyelinated fibers in the mice that received late treatment, but without reaching WT levels. In contrast, these morphological abnormalities were fully rescued in the mice that underwent extended early treatment, reaching WT levels (Figure 7, A–L). The degree of myelin pathology remained too mild in the sciatic nerves of 10-month-old CMT1A mice to be considered as a treatment readout (Supplemental Figure 24).

**Figure 8:**
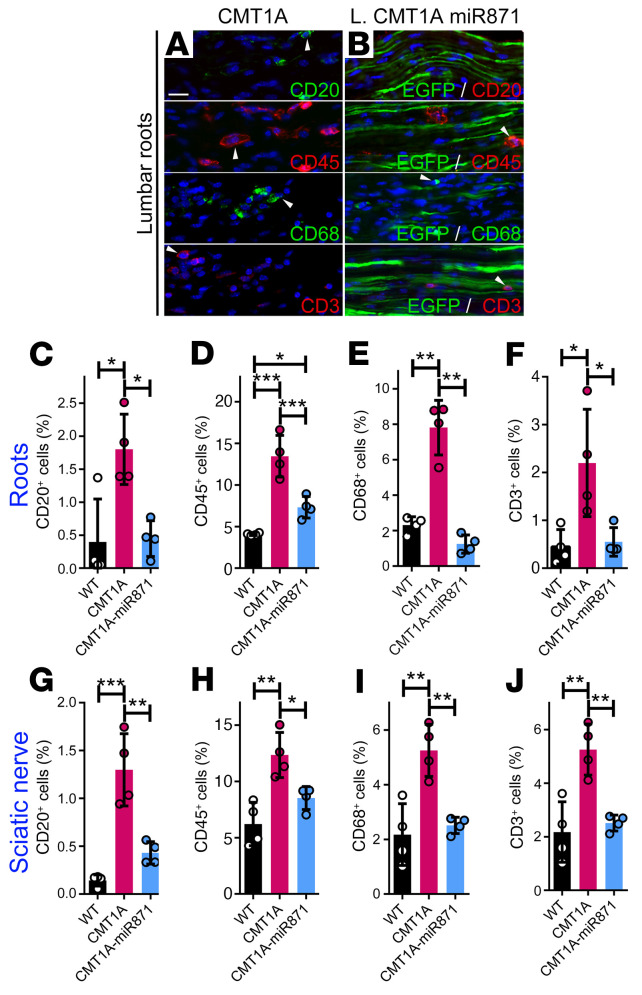
Late treatment of CMT1A mice improved inflammation in PNS tissues. Images of longitudinal lumbar spinal root sections from noninjected and late-treated CMT1A-AAV9-miR871 mice immunostained for CD20, CD45, CD68, and CD3 markers (**A** and **B**). The injected tissues were counterstained with the nuclear marker DAPI (blue) and EGFP (green) autofluorescence. Arrowheads indicate representative CD^+^ cells. Quantification of the percentage of inflammatory cells in lumbar roots (**C**–**F**) and sciatic nerves (**G**–**J**). Values represent the mean ± SD (*n =* 4/group). *P < 0.05, **P < 0.01, and ***P < 0.001, by 1-way ANOVA with Tukey’s multiple-comparison test. Scale bar: 20 μm (immunostained images are also shown in Supplemental Figure 25).

As in the early treatment group, analysis of inflammation by immunofluorescence revealed that late treatment with AAV9-miR871 decreased the numbers of CD20^+^, CD45^+^, CD68^+^, and CD3^+^ cells in PNS tissues (Figure 8 and Supplemental Figures 25 and 26). Injection with the therapeutic vector did not cause any inflammatory responses in the liver 4 months after injection (Supplemental Figure 27).

## Discussion

CMT1A is the most common inherited demyelinating neuropathy, resulting from a *PMP22* gene dosage effect in SCs. Ideally, CMT1A therapies should reduce overexpressed *PMP22*, while avoiding excessive knockdown that could lead to the milder phenotype of hereditary neuropathy with pressure palsies (HNPP). We accomplished that here with our study, which, to our knowledge, presents the first translatable AAV9-mediated *PMP22* gene–silencing approach leading to phenotypic improvement in a CMT1A mouse model. Although this is a preclinical study, we designed our approach from the outset with an eye toward translation to prospective human clinical trials, in 2 ways. First, the therapeutic construct is applicable to animal models and patients with CMT1A alike. The most relevant animal models, such as the C61-het mouse we used here, express transgenic copies of human PMP22 on a normal mouse background. Thus, both murine *PmP22* and human *PMP22* genes contribute to excessive gene dosage, leading to CMT1A-like phenotypes, and testing a translatable approach in mice requires targeting both transcripts. Importantly, the artificial miRNA we designed, miR871, targets both human *PMP22* and murine *Pmp22* transcripts. Second, we delivered AAV9-miR871 through a clinically applicable method of lumbar intrathecal injection into C61-het CMT1A mice, which reproduces the clinical course, severity, and symptoms of patients with CMT1A.

The translatability and effectiveness of the intrathecal administration route have been demonstrated in larger animals (82, 83) and in human trials (NCT03381729, NCT02362438), which showed effects in nerves distal to the injection site, including transduction of SCs in the tibial nerve of dogs after intrathecal injection of AAV9 (83). Another route of administration that could be easily translated to the clinic is intravenous injections. However, our studies in mice showed that intrathecal injection provides adequate biodistribution throughout the PNS, with much lower vector amounts injected compared with intravenous delivery (84). Intraneural injections of AAV.shRNA were also proposed to treat a CMT1A model (73), however, the translatability of this delivery method is considered challenging in the clinic.

In addition to incorporating species conservation and a feasible route of administration into our study design, we also demonstrated efficacy at multiple levels. First, we confirmed in vitro and in vivo the *PMP22/Pmp22* silencing efficiency of miR871 and its effects on other myelin-related genes and proteins, while also assessing the transduction efficiency of AAV9 in PNS tissues after lumbar intrathecal injection (Figures 1 and 2). We then demonstrated by multiple outcome measures the therapeutic effects of AAV9-miR871 after treatment at both early and later stages of the neuropathy, supporting the relevance of this approach for direct clinical translation to treat CMT1A. As demonstrated through our detailed baseline longitudinal, functional, and morphological analyses, the C61 het model of CMT1A used in this study develops an early-onset, progressive demyelinating pathology that reproduces human disease features (Supplemental Figures 2–7). Thus, already at the early intervention time point, the model presented significant pathological features and slowing of nerve conduction velocities that progressed with aging. Therefore, both early and late treatments represent post-onset interventions, reproducing the clinical scenario of treating younger or older patients with CMT1A, in whom demyelination is already present in childhood (85, 86), followed by slowly progressive axonal loss (8, 9, 87, 88). Our mouse data suggest that earlier treatment is effective, as several outcome measures were corrected to WT levels (Figures 3–5). A direct comparison of CMT1A mice that received extended early or late treatment, injected at 2 or 6 months of age, respectively, and analyzed at 10 months of age, confirmed that treatment more efficiently reversed disease manifestations if given earlier (Figures 6 and 7). This could be explained by the fact that later stages of the neuropathy were characterized by already advanced axonal degeneration. While it appears feasible to stimulate remyelination by transduced demyelinating SCs, increasing axonal loss found at later stages is irreversible. Nevertheless, our data suggest that the ability to improve CMT1A-like symptoms in mice with preexisting pathology is promising for the translation of this strategy to patients who may already be experiencing the effects of CMT1A. Indeed, our work is consistent with the findings of a tetracycline-inducible *Pmp22*-transgenic mouse study (89). *Pmp22* overexpression occurred in the absence of tetracycline, causing demyelination and numerous neuropathic phenotypes. Importantly, when mice were given tetracycline, thereby shutting off the *Pmp22* transgene, myelin normalization began to occur within 1 week, with nearly normal myelin observed by 4 months. Together, these data and ours suggest that some CMT1A phenotypes may be reversible. It is also possible that we will see an even greater reversal of phenotypes in these mice as they age 4 months after treatment.

Another question we considered, regarding translation, was the necessity to restrict miR871 expression to SCs alone. In our previous studies (84), we demonstrated that an AAV9 vector expressing a transgene through the SC-specific *Mpz* promoter efficiently transduced myelinating SCs throughout the PNS following a single lumbar intrathecal injection. In the current study, we used AAV9 to deliver a U6.miR.CMV.EGFP construct, in which both sequences (EGFP and miR871) were driven by ubiquitous promoters (CMV and U6, respectively) (Figure 1D). We calculated transduction rates via immunofluorescence using the CMV.EGFP reporter gene and VGCN analysis (Figure 2). Not surprisingly, expression was more widespread, with both SCs and other cell types transduced, including motor and sensory neurons, leading to axonal expression. We also detected VGCNs in many non-PNS tissues typically transduced by AAV9, with the highest numbers detected in the liver (Supplemental Figures 12 and 19), but without any apparent toxic effects in lumbar roots, sciatic nerves, liver, or DRGs (Figures 5 and 8, and Supplemental Figures 8–11, 16–18, and 25–27). Given that PMP22 expression levels are normally very low and do not have any known effects in other cell types besides myelinating SCs (11, 90–92), we do not expect any adverse effects from ubiquitous silencing of PMP22 expression. It is also important to mention that transduction evaluation through a reporter gene may not directly correlate with miR expression, when using 2 different ubiquitous promoters. An emerging area of study involves the ability of miRs to travel outside transduced cells through exosomes and potentially act at distant sites and neighboring cells. We did not directly measure exosome packaging of miR871, but future work should determine the potential for incomplete transduction leading to broader correction in adjacent, nontransduced cells.

The 5e11 vg/mouse vector dose used for intrathecal injection in this study corresponds to approximately 2.3e13 vg/kg. With this dose, we achieved sufficient SC transduction and PMP22 silencing to improve molecular, histopathological, and functional deficits. As such, this dose is comparable to those used in prior clinical AAV9 gene therapy studies that targeted motor neurons in the spinal cord, including the Avexis SMA trial (NCT03381729) for intrathecal delivery of Zolgensma 1.2e13vg/kg (93) and the giant axonal neuropathy (GAN) clinical trial (NCT02362438), in which intrathecal doses ranged from 3.5 × 10^13^ to 3.5 × 10^15^ total vg/patient (~1.75 × 10^12^ vg/kg to 1.75 × 10^13^ vg/kg) (94). Given that our dose was slightly higher than those currently used in clinical trials and the fact that our potential treatment population would be mostly adults with CMT1A, a dose escalation study would be useful for identifying the optimal vector concentration that would provide robust therapeutic benefit and minimal risk of *PMP22* haploinsufficiency.

To examine the potential side effects of *PMP22/Pmp22* oversilencing, we tested AAV9-miR871 in WT mice (Supplemental Results and Supplemental Figures 28–34). Despite muPmp22 levels being significantly reduced in WT-injected mice, we found only mild functional and electrophysiological abnormalities without the typical HNPP-like phenotype (95). Since our treatment is not intended to be applied to individuals with normal levels of PMP22 expression, the partial phenotype observed in WT mice based on the dual human-murine targeting capacity of miR871 does not raise safety concerns regarding the potential treatment of patients with CMT1A. This set of experiments in WT mice also underscores the importance of targeting all sources of *PMP22/Pmp22* in an animal model, especially when performing dose-finding studies to determine potential clinical doses.

Several therapeutic approaches for CMT1A have been suggested so far (61, 68), with the most clinically advanced being oral PXT3003. Although PXT3003 was shown to improve the symptoms of CMT1A in rats (56) and humans (60), its *Pmp22*-silencing efficiency was shown only at the mRNA level in the rat overexpressing murine *Pmp22*. It is still unclear how PXT3003 affects human PMP22. Although other pharmacological treatments have been suggested through the years, most of them are symptomatic, require repeated treatment sessions, or have potential long-term side effects. For example, intravenously delivered squalenoyl siRNA PMP22 nanoparticles (67) have been shown to provide therapeutic benefit in JP18/JY13 mice overexpressing the human *PMP22* gene. However, potential toxicity with repeated dosing and long-term stability, as well as the effects of this treatment on PMP22 mRNA or protein levels, remain to be shown. On the contrary, a gene therapy approach like ours would provide a one-off treatment option. In a previously reported preclinical gene therapy approach, AAV2/9 vectors expressed shRNAs specifically designed to target murine *Pmp22* (73). Because the shRNAs contain potentially disruptive mismatches with the human transcript, their direct translatability in humans was untested and remains unclear. Moreover, the shRNA vectors were delivered through direct intraneural injection, a method that is more difficult to translate to clinical practice for treating CMT1A and carries more risks because of the toxic nature of concentrated anesthesia and the risk of direct fiber damage (96). In contrast, the lumbar intrathecal injection used in our study is considered a routine procedure that can be easily applied in the clinic, providing widespread biodistribution in the PNS. Compared with intravenous delivery, intrathecal delivery also requires a much lower viral volume to provide beneficial effects and hence results in lower toxicity (84, 97). It remains to be shown that adequate biodistribution can also be achieved in larger animals before clinical translation.

Regarding the safety of AAV9-based vectors in humans, follow-up studies in AAV9-treated patients with spinal muscular atrophy (SMA) suggested stable beneficial effects of Zolgensma with no major adverse reactions or long-term toxicity (93, 98, 99). However, more recent studies suggest that long-term overexpression of proteins (100) or miRNAs (101) via AAV9 viral vectors may dysregulate endogenous mechanisms, causing toxic side effects. Our data suggested that AAV9-miR871 treatment did not cause inflammation in PNS tissues, as had been previously suggested in another study using the AAV9 serotype carrying a different payload (102), but in fact acted to reduce inflammation native to the CMT1A animal model. Moreover, injection with the therapeutic vector did not cause any chronic inflammatory responses in the liver 4 months after injection (Supplemental Figures 18 and 27). Although our approach was shown to improve the baseline inflammatory status of the CMT1A model without causing any systemic or liver toxicity, it will be important to consistently demonstrate its safety with more detailed toxicity studies across different species. Potential cellular and humoral immune responses can be stimulated against the AAV capsid or protein-coding gene product. Since our one-off therapeutic payload is a noncoding RNA, our vector should be inherently less immunogenic than those used in gene replacement strategies.

To design successful clinical trials involving patients with CMT1A, it is important to establish relevant and sensitive outcome measures. The gene-silencing approach described here provides functional improvements that can be easily evaluated in patients through electrophysiological testing. Since previous clinical trials suggested a lack of sensitivity of standard CMT clinical scores to detect treatment response (45, 103), more detailed clinical functional and patient-reported outcome measures will also be necessary (104–107), along with MRI-based quantification of muscle atrophy (108). Here, we also demonstrate, for the first time to our knowledge, the responsiveness of NF-L (109, 110) and Gdf15 (80, 81) plasma biomarkers in a CMT1A model. Responsiveness of these translatable biomarkers is highly encouraging for their utility in parallel clinical trials of miRNA therapies. Although, a recent study (79) showed a lack of correlation of NF-L plasma levels with disease progression over time in patients with CMT1A, this might be due to the older age of the patients tested (mean age, 46 years). In this regard, validation of additional clinically relevant plasma and skin biomarkers as indicators for future gene therapy efficacy would be essential (19, 111, 112, 113).

In conclusion, we developed and characterized an artificial miRNA designed to specifically target human *PMP22* and murine *Pmp22* transcripts and evaluated the therapeutic benefit in a CMT1A mouse model that reproduces CMT1A-associated phenotypes. Our results indicate that a single lumbar intrathecal injection of AAV9-miR871 at early and late stages of the neuropathy, and always after onset, can correct the functional, morphological, and inflammatory abnormalities of CMT1A without causing any apparent side effects. Taken together, these results constitute an important step toward the development of a clinically relevant and translatable gene therapy to treat CMT1A.

## Methods

All materials and methods are presented in the Supplemental Methods.

### Statistics.

Each set of data is presented as the mean ± SD or SEM, with *n* equal to the number of biological repeats for in vitro experiments or independent samples from individual animals for in vivo experiments. For comparison of means between 2 independent groups, an unpaired, 1-tailed Student’s *t* test was performed. For comparison of means between 3 or more independent groups, 1-way ANOVA was performed. Statistical significance for all experiments was defined as a *P* value of less than 0.05. When ANOVA tests suggested significant difference among groups, Tukey’s multiple-comparison post hoc test was applied. When a sample group was used for more than 1 comparison, Bonferroni’s correction of *P* values was additionally applied. All statistical analyses were performed using GraphPad Prism, version 6 (GraphPad Software).

### Study approval.

All animal procedures were approved by the Cyprus Government’s chief veterinary officer (project license CY/EXP/PR.L3/2017) according to national law, which is harmonized with European Union guidelines (EC Directive 86/609/EEC).

## Author contributions

MS co-designed and conducted or directed all experiments, acquired and analyzed data, created figures and legends, and drafted and reviewed the manuscript. AK performed electrophysiology experiments. SGC performed and analyzed in vitro screening of artificial miRNAs. MJJ and RH standardized, performed and analyzed ELISAs for serum Gdf15 levels. LMW contributed to in vitro screening of artificial miRNAs. AMF assisted with viral vector production. AH and HZ performed and analyzed plasma NF-L levels. JR , CT, and CC performed and analyzed VGCNs. SQH designed artificial miRNAs and supervised in vitro screening, created figures and legends, and drafted and reviewed the manuscript. KAK co-designed and supervised all experiments and drafted and reviewed the manuscript. All authors read and approved the final manuscript.

## Supplementary Material

Supplemental data

## Version 1 05/17/2022

In-Press Preview

## Version 2 07/01/2022

Electronic publication
