# Supplementary material for: A translatable RNAi-driven gene therapy silences PMP22/Pmp22 genes and improves neuropathy in CMT1A mice
Source: J Clin Invest. 2022 Jul 1;132(13):e159814. doi: 10.1172/JCI159814 (PMC9246392; doi:10.1172/JCI159814)
Supplement: Supplemental data [file jci-132-159814-s021.pdf]

MATERIALS AND METHODS

Study design

The goal of this study was first to design new U6 promoter-driven artificial miRs and assess their efficiency to silence human *PMP22* and murine *Pmp22 in vitro*, and then package the lead sequence into AAV9 viral vector for *in vivo* efficacy studies in C61-het mice, a model of CMT1A. *In vitro* assessment was performed in HEK293 cells co-transfected with CMV-driven hu*PMP22* or mu*Pmp22* plasmids along with the candidate miRs, followed by RT-qPCR to quantify gene silencing efficacy.

For *in vivo* analysis, C61 heterozygous (C61 het) mice were intrathecally injected with AAV9-miR871 or control expressing miRLacZ. For validation of miR871 silencing efficacy injected mice were analyzed 6 weeks after injection using immunohistochemistry (n=4/group), VGCN analysis (n=4/group), RT-qPCR (n=3/group) and Western blot (n=3/group). For evaluating the therapeutic effect of targeting miR versus non-targeting miR early (n=16/group), extended early (n=16/group) and late treatment (n=16/group) trials were conducted.

Early- and late- treated animals were injected at 2 or 6 months of age, respectively, and their motor performance was monitored every two months with behavioral testing until 4 months post-injection. At the final time point of each treatment group, mice were analyzed for *PMP22/Pmp22* downregulation and for the expression of other myelin related genes/proteins with RT-qPCR and WB; with nerve electrophysiology testing measuring MNCV and CMAP; axonal degeneration biomarkers including circulating NF-L and Gdf15 levels; PNS tissues morphological evaluation using semithin sectioning analysis; inflammatory response using immunohistochemistry; and finally AAV9 vector biodistribution with VGCN analysis.

Extended early treated animals were injected at 2 months of age and their motor performance was monitored every two months with behavioral testing until 8 months post-injection. At the final time point of this treatment group mice were also analyzed for MNCV and CMAP scores with nerve electrophysiology testing, for axonal degeneration with plasma NF-L levels analysis, for PNS tissues morphological evaluation using semithin sectioning analysis and for AAV9 vector biodistribution with VGCN analysis.

miR sequence generation and cloning

Artificial miRs were designed based on natural human mir-30, maintaining important structural and sequence elements required for normal miRNA biogenesis but replacing mature mir-30 sequences with 22-nt of complementarity with the *PMP22* gene. Criteria for miRNA selection were previously described (1, 2). Briefly, a potential 22 nucleotide mature miRNA duplex must meet four criteria (relative to the mature antisense miRNA sequence): (1) the four 3’ nucleotides must be at least 75% G:C; (2) the four 5’ nucleotides must be 75% A:U; (3) Overall A:U content of at least 40%; (4) lack of RNA pol III termination signal (TTTTTT) in the pre-miRNA sequence. All sequences were cloned as DNA transcription templates in front of the mouse U6 promoter into

the U6T6 plasmid as described (1), sequenced verified and used for in vitro screening prior to AAV vector production. Control microRNAs targeting EGFP or LacZ were previously described (3, 4).

In vitro screening of miRs: Transfection and cells RNA extraction

HEK293 cells were obtained from ATCC® (CRL-1573™). HEK293 cells were grown using DMEM (Gibco, 10–569-044) medium supplemented with 20% FBS (Corning, 35–011-CV), 1% L-glutamine (Gibco, 25030081) and 1% penicillin–streptomycin (Life Technologies, 15140–122). Transfected cells were grown in the same DMEM medium but lacking penicillin–streptomycin.

HEK293 cells were co-transfected using Lipofectamine 2000 (Invitrogen, 11668027) with a plasmid expressing either mu*Pmp22* or hu*PMP22* gene and a plasmid expressing the U6.miRPMP22 miRs, or controls, at a molar ratio 1:4. Negative controls included empty U6T6 plasmid (no miR) or one expressing a miRNA targeting EGFP (miGFP). Twenty-four hours after the co-transfection, RNA was isolated from the cells using Trizol Reagent (Ambion, 15596018) following manufacturers' protocol. After DNase treatment, RNA was quantified and 1 μg of RNA was used to synthesize cDNA using High-Capacity cDNA Reverse Transcription Kit (Applied Biosystems, N8080234). Then levels of hu*PMP22* (Hs00991884_m1), muP*mp22* (Mm01333393_m1) and huRPL13A (Hs04194366_g1) were quantified using Taqman gene expression assays (Applied Biosystems). Human RPL13A was used as an endogenous control. Data were collected from 3 independent experiments. All QPCR assays were performed in triplicate.

Production of AAV vectors

U6.miRPMP22 cassettes were subcloned into self-complementary AAV backbones (5) containing separate CMV.eGFP cassettes. Viral vectors were produced by Andelyn Biosciences (Ohio, US) using triple transfection into HEK293 cells of pro-viral plasmids, pHELPER (containing adenoviral helper genes), and a plasmid containing AAV2 rep and AAV9 cap genes. The 3' inverted terminal repeats (ITR) of AAV2 contained a deletion of the terminal resolution site (trs) to enable formation of double-stranded AAV genomes (6). All vectors were purified by iodixanol gradient ultracentrifugation followed by FPLC purification, then titrated by ddPCR using primer/probes to detect the AAV2 ITR.

Experimental animals and lumbar intrathecal injection

All experimental procedures in this study were conducted in accordance with animal care protocols approved by the Cyprus Government's Chief Veterinary Officer (project license CY/EXP/PR.L3/2017) according to national law, which is harmonized with EU guidelines (EC Directive 86/609/EEC). All inoculations were performed under anesthesia and all efforts were made to minimize animal suffering. The protocols were approved by Cyprus Government's Chief Veterinary Officer. In this study, we used adult WT C57BL/6 or C61 Het mice. The C61 Het colony was established from two breeding pairs gifted by Prof R. Martini (Universitäts-Klinikum Würzburg, Germany). C61 Het mice carry 4 copies of human *PMP22* along with normal

endogenous murine *Pmp22* (7). Early, extended-early and late gene therapy trials were conducted using 2- or 6-month-old mice C61 Het mice. Mice were kept in a specific pathogen-free animal facility, housed in open-top system cages. Up to five mice were housed in cages linked with high absorbency wood bedding for laboratory mice, dried by high-temperature treatment, sieved, de-dusted, prior to use. Standard mouse diet, certificate for reproduction, weaning, growth, and tap potable water filtered and UV sterilized were administered to the mice. Mice were kept in a 12 h dark/12 h light cycle at a temperature of 22 °C. Both male and female mice were used in our experiments and showed no sex related differences in their behavioral performance or nerve pathology.

Intrathecal injection was performed as described before (8-14). Briefly, a small skin incision was made along the lower lumbar spine level of anesthetized mice to visualize the spine and the AAV vector was delivered into the L5-L6 intervertebral space. A 50-μL Hamilton syringe (Hamilton, 80530/00) connected to a 26-gauge needle (Hamilton, 7758-02/00) was used to inject 20 μL of AAV stock containing an estimated $5x10^{11}$ vector genomes (vg), at a maximum rate of 1 μL/15sec. A flick of the tail was considered indicative of successful intrathecal administration.

Immunofluorescence staining
For immunostaining, mice were anesthetized and then transcardially perfused with normal saline followed by fresh 4% paraformaldehyde (Merk, P6148) in 0.1M PB buffer. The lumbar-sacral spinal cords with spinal roots attached, as well as the bilateral sciatic and femoral motor nerves were dissected and post-fixed in 4% PFA, for 2 h (spinal cord) or 30 minutes (sciatic and femoral nerves). Spinal roots and sciatic nerves were frozen for cryosections while femoral nerves were isolated and teased into fibers under a stereoscope. Teased fibers or sections were permeabilized in cold acetone and incubated at RT with a blocking solution of 5% BSA (Sigma-Aldrich, A79061) containing 0.5% Triton-X (Sigma-Aldrich, T8787) for 1 h. Primary antibodies used were: mouse monoclonal antibody against CD3 (1:100; Abcam, ab5690), rat CD68 (1:50; Biorad, MCA1957A488), CD45 (1:100; Abcam, ab25386) and goat CD20 (1:100; Santa Cruz, sc-7735) all diluted in blocking solution and incubated overnight at 4 °C. Slides were then washed in PBS and incubated with mouse cross-affinity fluorescein-conjugated (1:1000; Invitrogen, A21202), rat cross-affinity purified rhodamine-conjugated (1:2000; Invitrogen, A21434) and goat cross-affinity fluorescein-conjugated (1:700; Abcam, ab150129) secondary antibodies for 1 h at RT. Cell nuclei were visualized with DAPI (1 μg/ml; Sigma, MBD0015). Slides were mounted with fluorescent mounting medium (Agilent-DAKO, S3023) and images photographed under a fluorescence microscope with a digital camera using a fluorescence microscope (Nikon Eclipse Ni) with a digital camera (DS-Qi2) using NIS-Elements software. EGFP was shown as green autofluorescence. Total cell numbers and number of positive cells were counted.

Vector genome copy number (VGCN) determination
VGCN was determined as already described in previous studies (13, 14). Briefly, genomic DNA was extracted from different PNS and CNS tissues (i.e., lumbar roots, proximal, middle and distal sciatic nerve, femoral nerve, brain, liver, trigeminal and spinal cord) of mice after intrathecal vector

delivery using the MagPurix Tissue DNA Extraction Kit (Zinexts Life Science, ZP02004). DNA yield and purity was quantified using a Nanodrop 1000 spectrophotometer. Approximately 20 ng of DNA was used as template for two real-time PCR assays on an Applied Biosystems 7500 Real-Time PCR System involving 45 cycles of 15 s at 95 °C and 60 s at 60 °C. TFRC-specific primers/probe targeting the mouse genome and EGFP-specific primers/probe, which is contained in the transgene, were used. Standard curves were created by serial dilution of quantified mouse genomic DNA, as well as quantified plasmid DNA containing the transgene cassette. The average VGCN per cell was calculated as the total VGCN divided by the total cell number.

Mouse RNA isolation and quantification using RT-qPCR
RNA was extracted from mouse lumbar roots, sciatic and femoral nerves using Qiagen RNeasy® Lipid Tissue Mini Kit (Qiagen, 74804) following the manufacturer's protocol from snap-frozen tissues. After DNase treatment (Qiagen, 79254), RNA was quantified by spectrophotometry and 0.3 μg of RNA was used to synthesize cDNA using TaqMan™ reverse-transcription reagents (Applied Biosystems, N8080234). Then the levels of hu*PMP22* (Hs00991884_m1), muP*mp22* (Mm01333393_m1), mu*Mpz* (Mm00485141_g1), mu*Cnp* (Mm01306641_m1), mu*Gldn* (Mm00616548_m1) and mu*Gjb1* (Mm01950058_s1) mRNA were quantified Taqman gene expression assays (Applied Biosystems) and muGapdh assay (Mm99999915_g1) as an endogenous control.

Immunoblot analysis
Fresh sciatic and femoral nerves and lumbar spinal roots were collected and lysed in ice-cold RIPA buffer (10 mM sodium phosphate, pH 7.0, 150 mM NaCl, 2 mM EDTA, 50 mM sodium fluoride, 1% Nonidet P-40, 1% sodium deoxycholate, and 0.1% SDS) containing a mixture of protease inhibitors (Roche, 11836170001). Proteins (150 μg) from the lysates were fractionated by 12% SDS/PAGE and then transferred to a PVDF membrane (GE Healthcare Life Sciences, 10600021) using a semidry transfer unit. Nonspecific sites on the membrane were blocked with 5% non-fat milk in PBS with Tween 20 (Sigma, P1379) (PBST) for 1 h at room temperature. Immunoblots were incubated with rabbit antisera hu PMP22 (1:500; Abcam, ab90782), mu/hu PMP22 (1:500; Abcam, ab211052), against EGFP (1:1,000; Abcam, ab6556) and mouse β-tubulin (1:4,000; Developmental Studies Hybridoma Bank, ε-7) at 4 °C overnight. After washing, the immunoblots were incubated with an anti-mouse (1:3000; Jackson ImmunoResearch, 115-036-068) or anti-rabbit HRP-conjugated secondary antiserum (1:3000; Jackson ImmunoResearch, 111-036-003) in 5% milk–PBST for 1 h. The bound antibody was visualized by an enhanced chemiluminescence system (Cytiva-Amersham; RPN2232). MPZ band was quantified from on Coomassie blue stained gel (Merk; 17-0518-01). For the quantification, optic density ratios were calculated using ImageJ software.

Behavioral testing
For *rotarod test*, animals were trained on an accelerating rotarod apparatus (Ugo Basile, 7650) for three consecutive days by three trials per day with 15-min rest period between trials. The mice were placed on the rod, and the speed was gradually increased from 2.5 to 25 rpm. The trial lasted

until the mouse fell from the rod or after the mouse remained on the rod for 600 s and was then removed. Testing was performed on the fourth day using two different speeds, 5 and 17.5 rpm. Latency to fall was calculated for each speed.

To measure *hind limb grip strength*, a mouse was held by the tail and lowered towards the apparatus (Ugo Basile, 57107) until it grabbed the grid only with the hindlimb paws. Mice were gently pulled back until they released the grid. Each session consisted of six consecutive trials. Measurements of the force in g were indicated on the equipment. Hind limb force was calculated by averaging the scores of each trial for each animal.

For *wire hang testing*, animals were placed atop a wire, which was then inverted; causing the mice to hang from the paws. Latency to fall was then recorded. This test was performed once a day for three days and then the average performance was calculated and reported.

For *hindlimb clasping evaluation*, mice were suspended by the base of the tail and three pictures captured every 5 seconds. The average angle of each mouse hindlimb opening was calculated using ImageJ software.

Electrophysiological analysis

For MNCV and CMAP measurements, the bilateral sciatic nerves were stimulated in anesthetized animals at the sciatic notch and distally at the knee via bipolar electrodes with supramaximal square-wave pulses (5 V) of 0.05 ms. MNCV was calculated by dividing the distance between the stimulating and recording electrodes by the result of subtracting the distal latency from the proximal latency. The latencies of CMAP were recorded by a bipolar electrode inserted between digits 2 and 3 of the hind paw and measured from the stimulus artefact to the onset of the negative M-wave deflection. A fixed distance was used between distal stimulation and recording sites for calculating distal latency to avoid errors arising from variations in knee-paw distance in each mouse.

Plasma neurofilament light (NF-L) levels

Blood samples were collected from retro-orbita as previously described and processed within one hour (14). Blood samples were collected in EDTA-containing tubes and centrifuged at 20 °C at 3500 rpm for 10 minutes. Centrifugation separated blood samples in two phases and the top plasma phase was collected and stored at −80 °C until testing. Plasma NF-L concentration was measured at University College London (UCL) using a commercially available NF-Light kit on a Single molecule array (Simoa) HD-X instrument (Quanterix) (15, 16).

Serum growth differentiation factor 15 (Gdf15) levels

Blood samples were collected from retro-orbita as previously described (17). Blood samples were collected in serum separation tubes, allowed to clot for 15-30 minutes and centrifuged at 20 °C at 3000 g for 10 minutes. Serum stored at −80 °C until testing. Serum protein levels were determined at the Department of Clinical Neurosciences, University of Cambridge, by ELISA according to manufacturer's instructions for growth differentiation factor 15 (R&D Systems, MGD150).

Morphometric analysis of myelination in lumbar roots and peripheral nerves

Morphometric analysis was performed as described before (9, 11, 14). Mice were transcardially perfused with 2.5% glutaraldehyde (Agar, R1010) in 0.1M PB buffer. The lumbar spinal cord with multiple spinal roots attached, as well as the femoral and sciatic nerves, were dissected and fixed overnight at 4 °C, then osmicated (SPI, 02601-AB), dehydrated, and embedded in resin (mixture of 17% Araldite resin (Agar,R1040), 25.5% Agar 100 (Agar,R1043), 55.5% dodecenylsuccinic anhydride (Agar, R1051), 2% 2,4,6-tri(dimethylaminomethyl)phenol (Agar,R1064)). Transverse semi-thin sections (1 μm) of the lumbar spinal cord with roots and the middle portion of the femoral motor and sciatic nerves were obtained and stained with alkaline toluidine blue (SPI,02576-AB). Sections were captured using a Nikon Eclipse Ni microscope with a digital camera (DS-Fi3) using NIS Elements software. Images of roots or transverse femoral nerve sections were obtained at 200x and 400x final magnification, respectively. Series of partially overlapping fields covering the entire cross-sectional area of the roots or the nerves were captured at 600× final magnification. Sciatic nerves detailed pictures were obtained at 1000x final magnification.

In brief, all demyelinated, thinly myelinated and normally myelinated axons were counted using the following criteria: axons larger than 1 μm without a myelin sheath were considered demyelinated; axons with myelin sheaths <10% of the axonal diameter were considered thinly myelinated; axons surrounded by circumferentially arranged SC processes and extracellular matrix were considered as “onion bulbs”; all other myelinated axons were considered normally myelinated.

SUPPLEMENTARY RESULTS

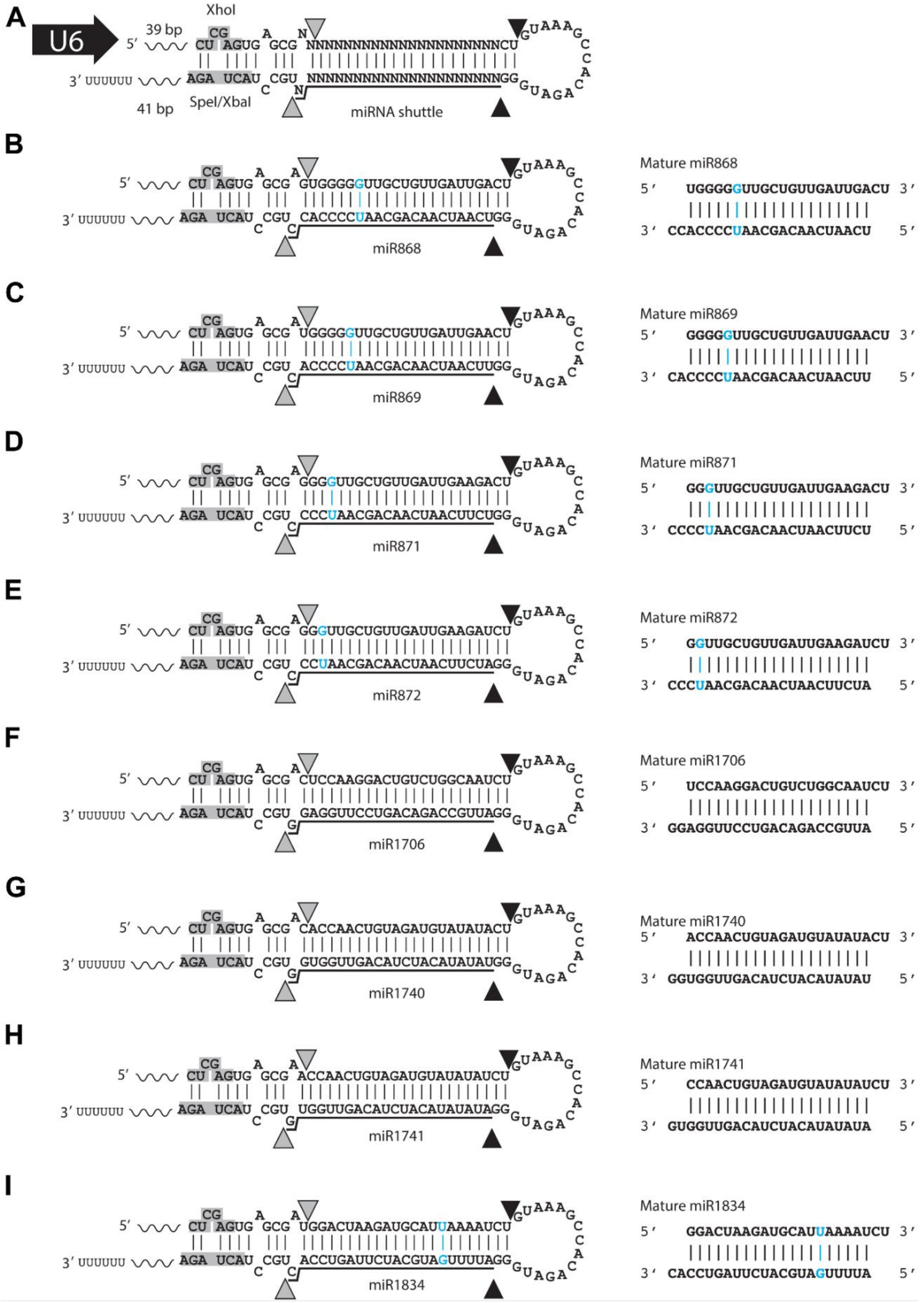

SUPPLEMENTARY DATA

Fig. S1. U6 miPMP22 loop structures and mature sequences. (A) Artificial miRNA shuttles standard design. **(B-I)** Collectively, design criteria suggested 8 miRNA sequence candidates that perfectly base-paired with human and mouse PMP22 mRNAs across the 22-nt guide RNA sequence (miR868, miR869, miR871, miR872, miR1706, miR1740, miR1741, miR1834). Gray and black arrowheads indicate Drosha and Dicer cut sites, respectively. The mature guide strand is underlined. Blue letters indicate wobble G-U bonds in RNA duplexes.

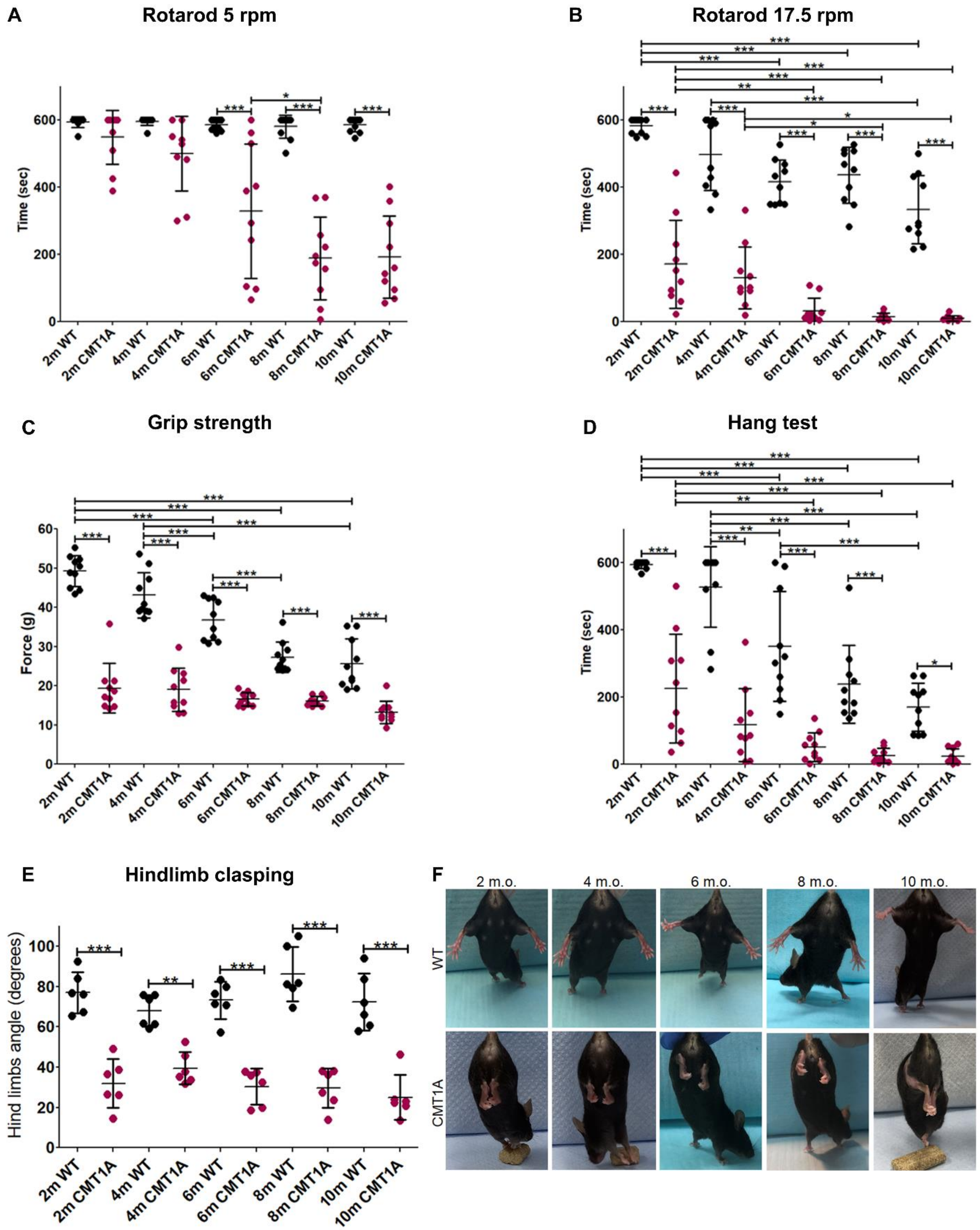

Fig. S2. CMT1A mouse model behavioral analysis demonstrates progressive functional impairment. CMT1A mice (C61 heterozygous) were compared to age-matched wild-type (WT) controls with rotarod at 5 rotations per minute (rpm) **(A),** and 17.5 rpm **(B)**, with grip strength test **(C)**,

with hang test **(D),** and with hindlimb opening testing (**E**), at 2, 4, 6, 8 and 10 months of age (n=5/group). Representative images of hindlimb clasping phenotype of WT and CMT1A mice at 2, 4, 6, 8 and 10 months of age are shown **(F)**. Values represent mean ± SD. Data were compared using One-way ANOVA with Tukey's Multiple Comparison Test. Significance level of all comparisons, P<0.05.

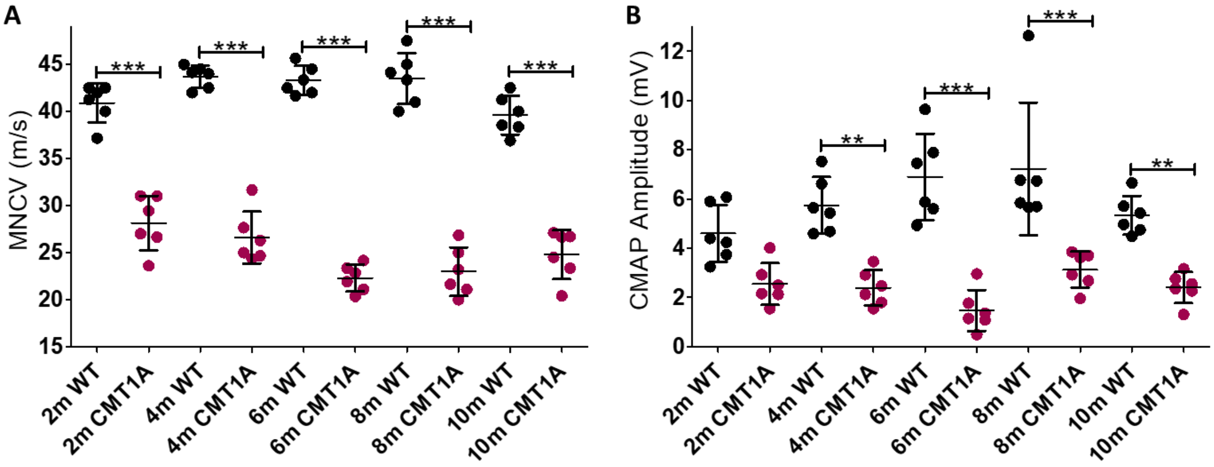

Figure S3: CMT1A mouse model electrophysiological analysis shows early onset progressive demyelination. MNCV **(A)** and CMAP **(B)** analysis of CMT1A mice were compared with age-matched WT controls at 2, 4, 6, 8 and 10 months of age. Values represent mean ± SD. Data were compared using One-way ANOVA with Tukey's Multiple Comparison Test. Significance level of all comparisons, P<0.05.

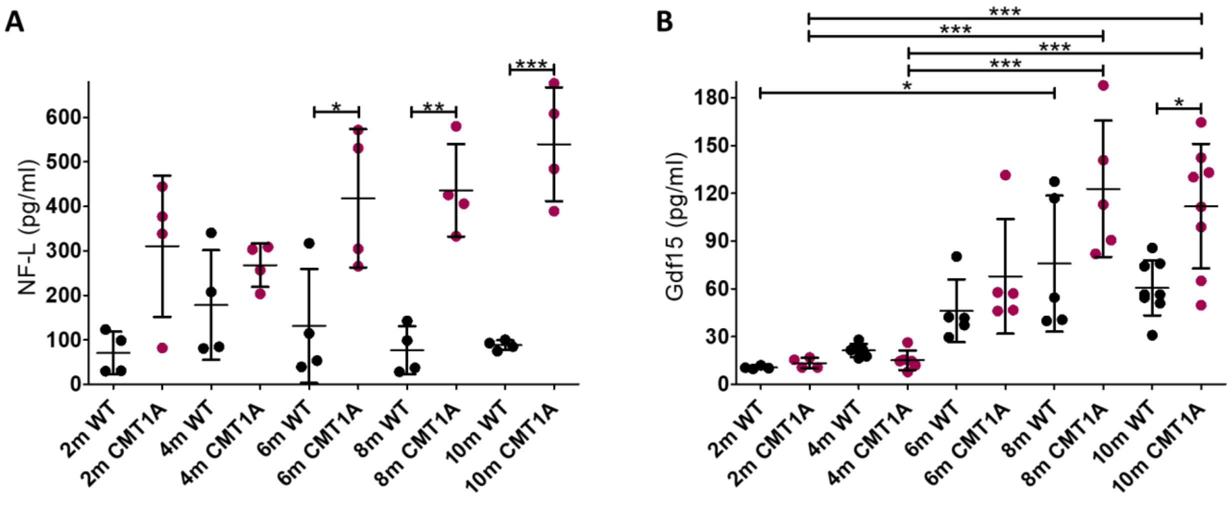

Fig. S4. Progressive elevation of circulating neurofilament light (NF-L) and growth differentiation factor 15 (Gdf15) levels in the CMT1A mouse model. NF-L **(A)** and Gdf15 **(B)** blood levels of CMT1A mice were compared with age-matched WT controls at 2, 4, 6, 8 and 10 months of age. Values represent

mean ± SD. Data were compared using One-way ANOVA with Tukey's Multiple Comparison Test. Significance level of all comparisons, P<0.05.

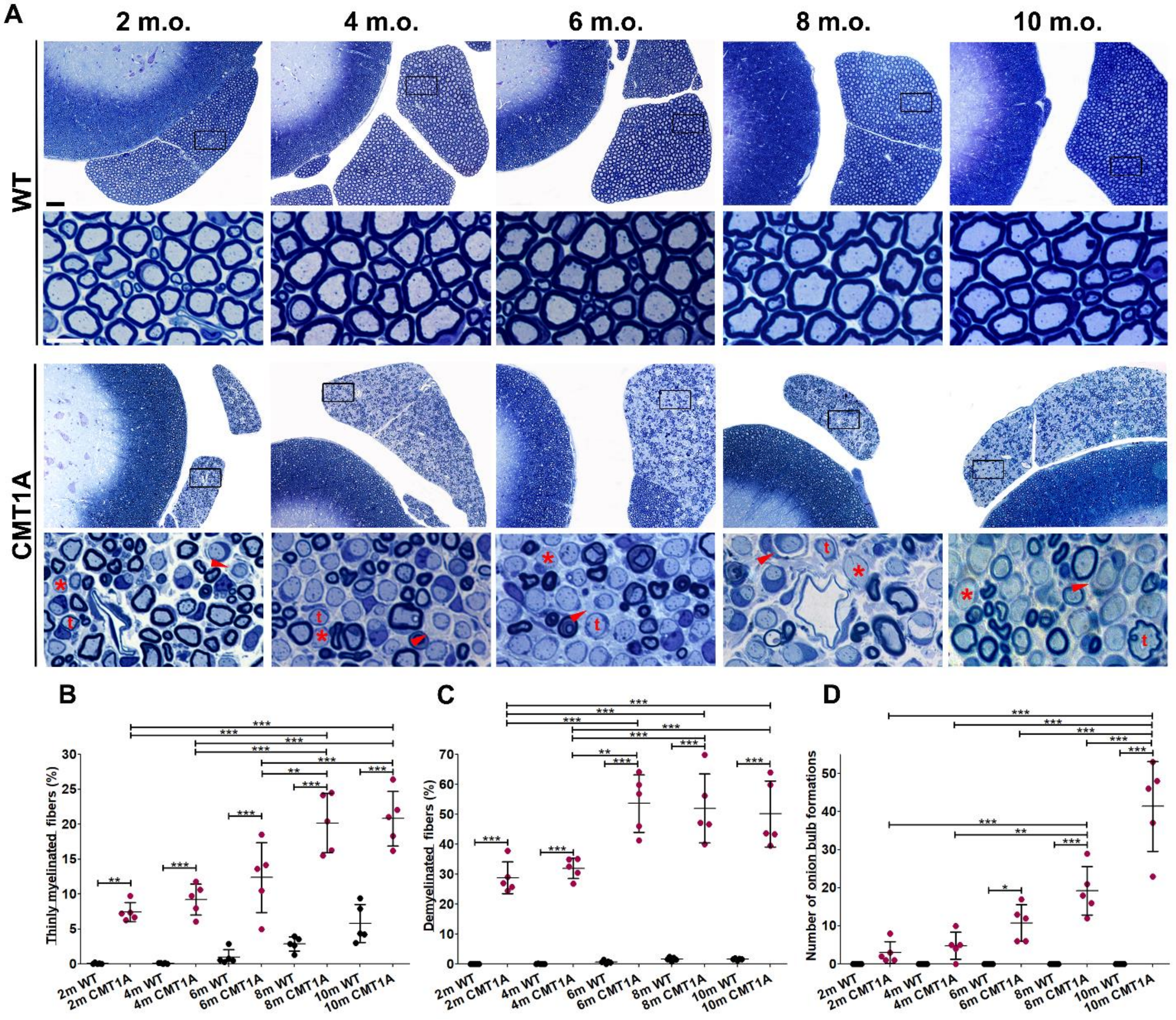

Fig. S5. Progressive demyelination in lumbar motor roots of the CMT1A mouse model. Representative images of WT and CMT1A semithin sections of spinal cord with spinal roots attached, stained with toluidine blue at 2, 4, 6, 8 and 10 months of age as indicated, at low (upper panels) and at higher magnification (lower panels) **(A)**. Thinly myelinated fibers (t), demyelinated fibers (*), as well as onion bulb formations (red arrowhead) are indicated. Quantification of these abnormalities in multiple roots of age-matched non-injected WT and CMT1A mice (n=5/group) shows progressive myelination defects **(B-D)**. Values represent mean ± SD. Data were compared using One-way ANOVA with Tukey's Multiple Comparison Test. Significance level of all comparisons, P<0.05. Scale bars: 50 μm, for magnified: 10 μm.

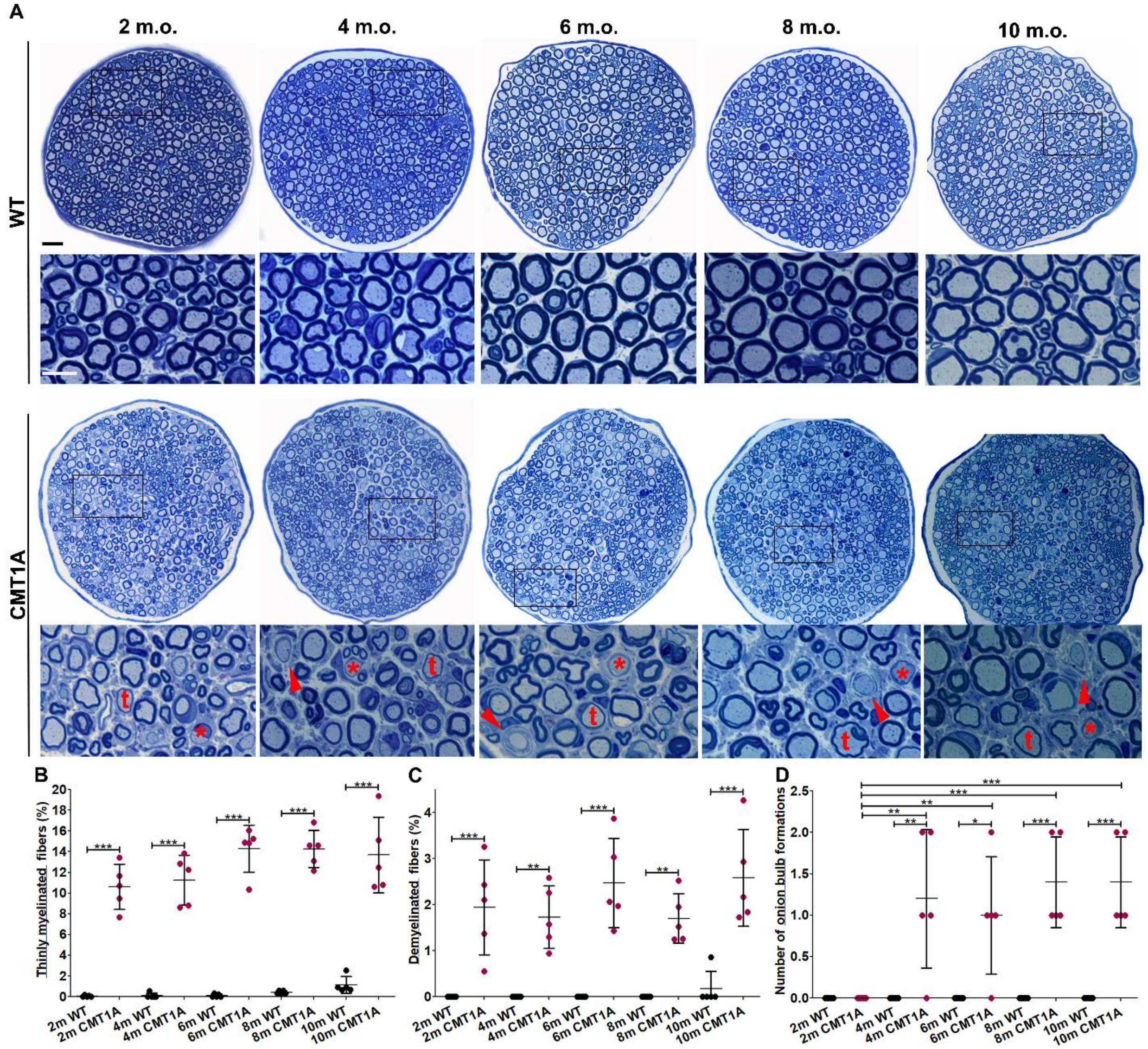

Fig. S6. Progressive demyelination in femoral motor nerves of the CMT1A mouse model. Representative images of WT and CMT1A semithin sections of femoral motor nerves stained with toluidine blue at 2, 4, 6, 8 and 10 months of age as indicated, at low (upper panels) and at higher magnification (lower panels) **(A)**. Thinly (t), demyelinated (*) fibers as well as onion bulb formation (red arrowheads) are indicated. Quantification of these abnormalities in femoral motor nerves from age-matched non-injected WT and CMT1A (n=5/group) is shown in **(B-D)**. Values represent mean ± SD. Data were compared using One-way ANOVA with Tukey's Multiple Comparison Test. Significance level of all comparisons, $P<0.05$. Scale bars: 20 μm, for magnified: 10 μm.

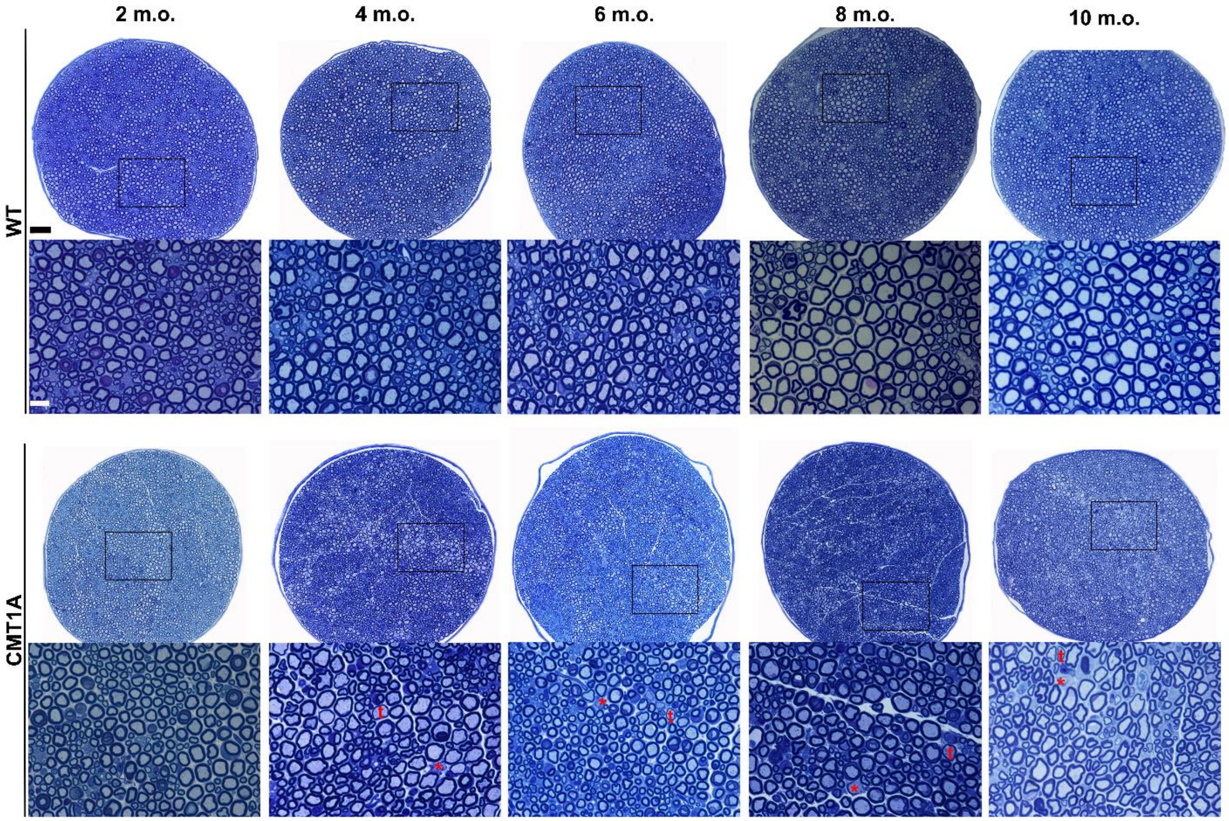

Fig. S7. CMT1A mouse model mid-sciatic nerve morphological analysis. Representative images of WT and CMT1A semithin sections of mid-sciatic nerve stained with toluidine blue at 2, 4, 6, 8 and 10 months of age at low magnification (upper) and higher magnification (lower). Thinly (t) and demyelinated (*) fibers are indicated, showing overall minimal pathological findings when compared to femoral motor nerve. Scale bars: 40 μm, for magnified: 10 μm.

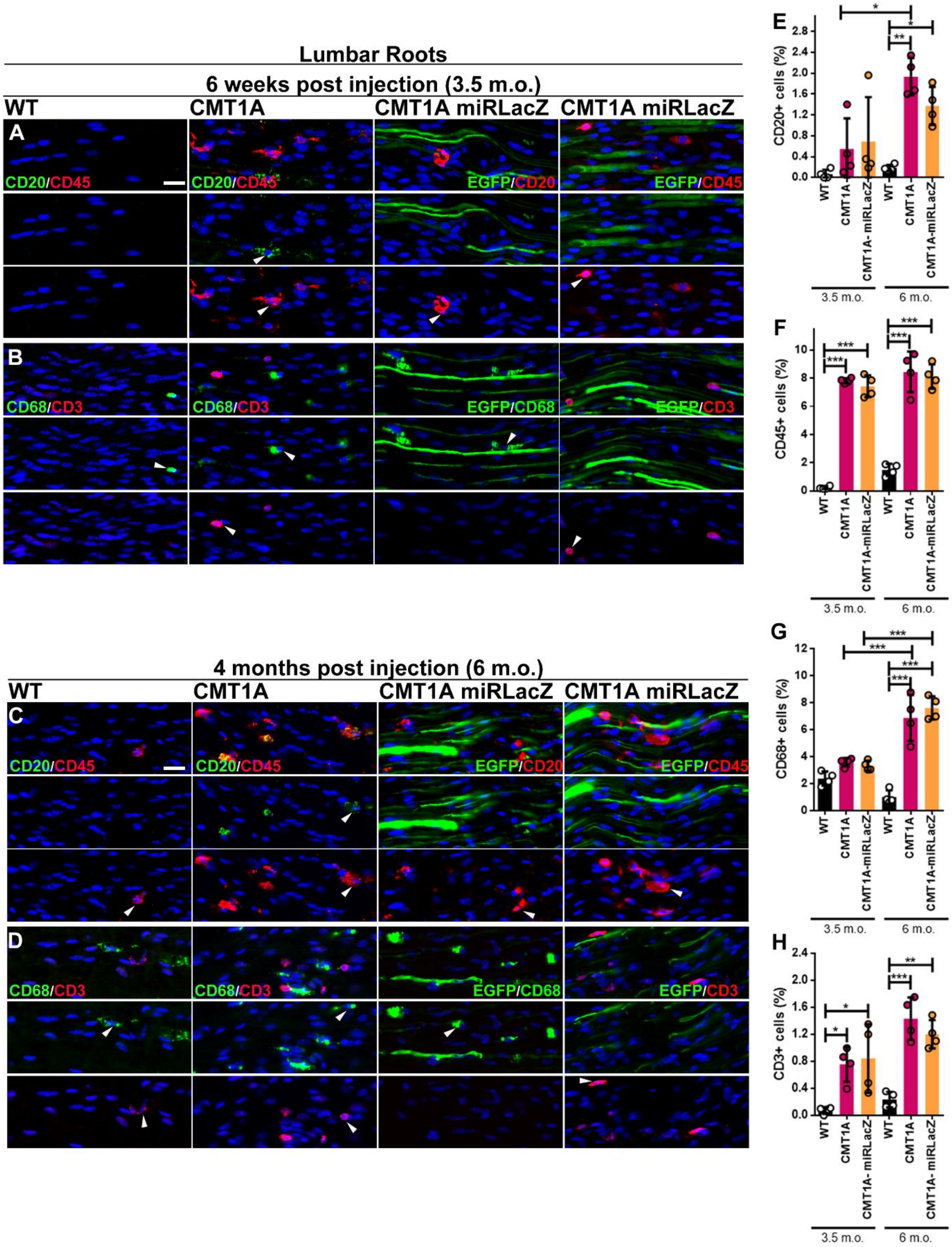

Fig. S8. Inflammatory infiltrates in the lumbar spinal roots of the CMT1A model at baseline and 6 weeks (3.5 months of age) or 4 months post injection (6 months of age) of AAV9-miRLacZ. Representative images of immunostained lumbar spinal roots of CMT1A mice injected with AAV9-

miRLacZ (n=4) at 6 weeks and 4 months post injection compared to age-matched non-injected WT (n=4) and CMT1A mice (n=4) with inflammatory cell markers as indicated **(A-D)**. Quantification of the percentage of cells positive for the B-cell marker CD20 (green) **(E)**, leukocyte marker CD45 (red) **(F)**, macrophage marker CD68 (green) **(G),** or T-cell marker CD3 (red) **(H),** was calculated in relation to the total cell number (Mean, SD). Nuclear staining with DAPI (blue). In tissues of AAV9-miRLacZ injected mice EGFP autofluorescence is also seen. Arrowheads are pointing to indicative CD+ cells. Data were compared using One-way ANOVA with Tukey's Multiple Comparison Test followed by Bonferroni correction. Significance level of all comparisons, $P<0.05$. Scale bars: 20 μm. Immunostaining images of 6-month-old non-injected CMT1A mice, as well as quantification data of 6-month-old non-injected WT and CMT1A mice are also used in Figure 5.

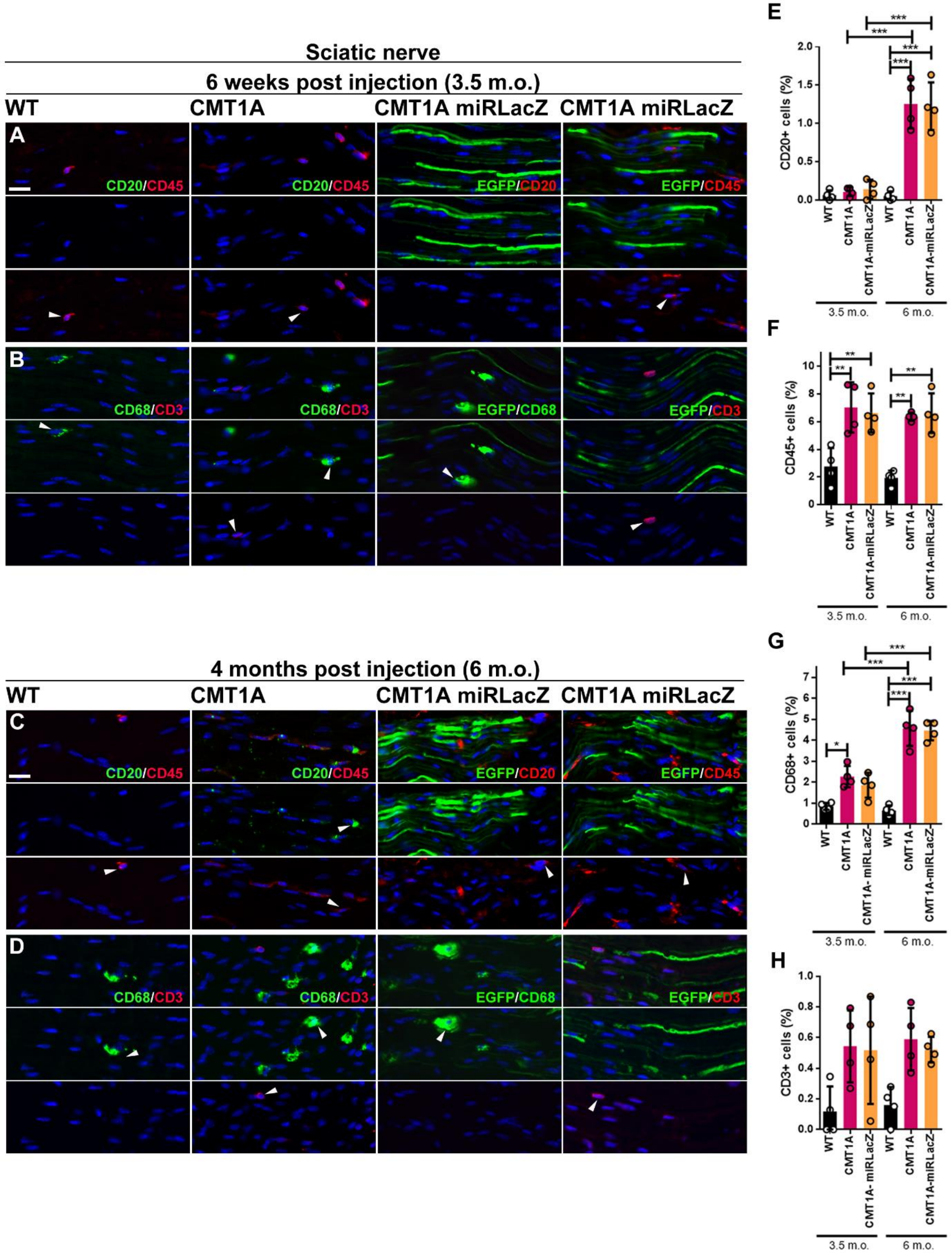

Fig. S9. Inflammatory infiltrates in the sciatic nerves of the CMT1A model at baseline and 6 weeks (3.5 months of age) or 4 months post injection (6 months of age) of AAV9-miRLacZ. Representative

images of immunostained sciatic nerve sections from CMT1A mice injected with AAV9-miRLacZ (n=4) at 6 weeks and 4 months post injection compared to age-matched non-injected WT (n=4) and CMT1A mice (n=4) **(A-D)**. Quantification of the percentage of cells positive for the B-cell marker CD20 **(E)**, leukocyte marker CD45 **(F)**, macrophage marker CD68 **(G),** or T-cell marker CD3 **(H),** was calculated in relation to the total cell number (Mean, SD). Nuclear staining with DAPI (blue). In tissues of AAV9-miRLacZ injected mice EGFP autofluorescence is also seen. Arrowheads are pointing to indicative CD+ cells. Data were compared using One-way ANOVA with Tukey's Multiple Comparison Test followed by Bonferroni correction. Significance level of all comparisons, $P<0.05$. Scale bars: 20 μm. Quantification data of 6 months old non-injected WT and CMT1A mice are also used in Figure 5.

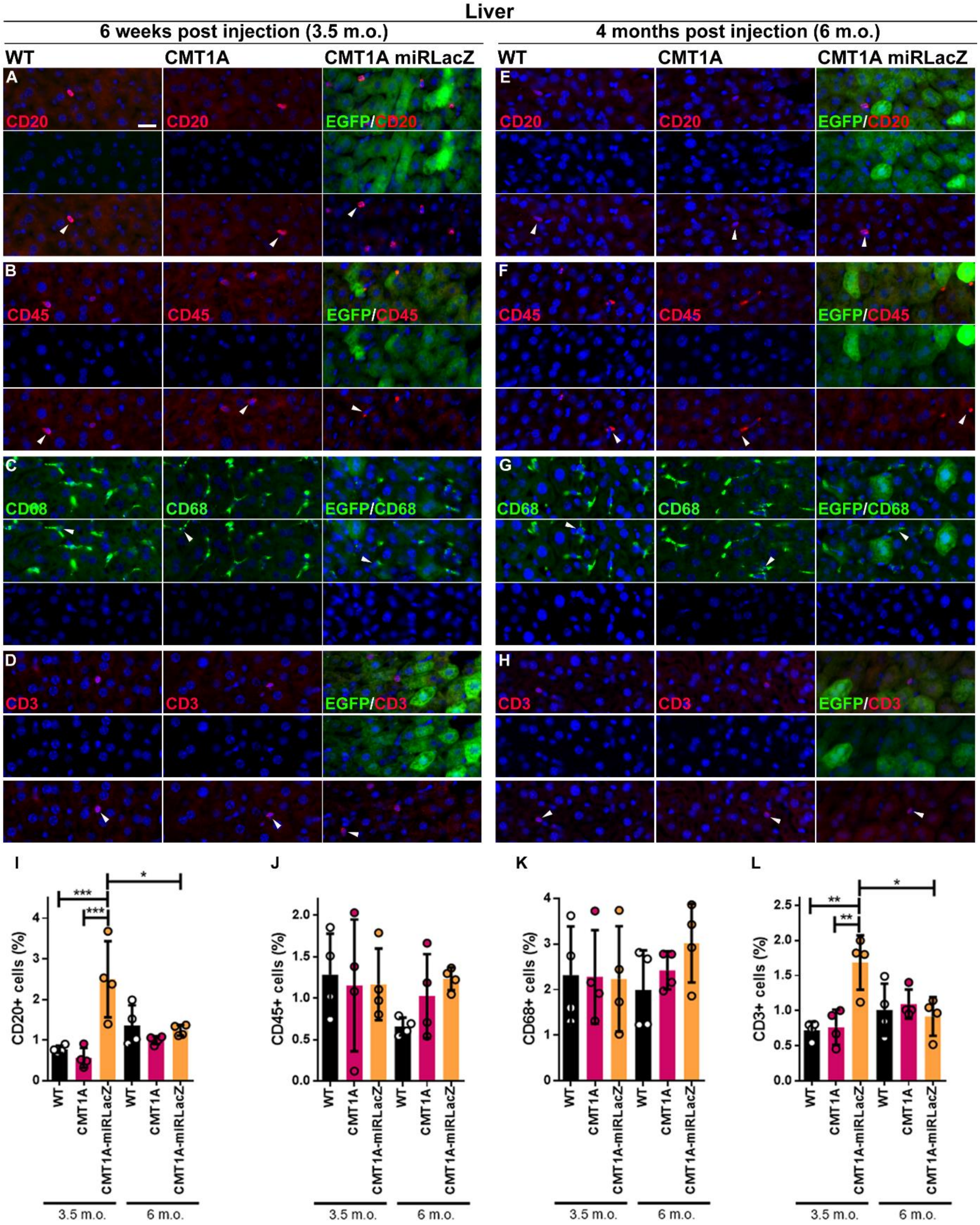

Fig S10. Inflammatory response in the liver of AAV9-miRLacZ-injected CMT1A mice at 6 weeks (3.5 months of age) and at 4 months post injection (6 months of age). Representative images of immunostained liver sections from CMT1A mice injected with AAV9-miRLacZ (n=4) at 6 weeks and 4 months post injection compared to age matched non-injected WT (n=4) and CMT1A mice (n=4), as

indicated **(A-H)**. Quantification of the percentage of cells positive for the B-cell marker CD20 (red) **(I)**, leukocyte marker CD45 (red) **(J)**, macrophage marker CD68 (green) **(K),** or T-cell marker CD3 (red) **(L)** was calculated in relation to the total cell number (Mean, SD). Nuclear staining with DAPI (blue). In tissues of AAV9-miRLacZ injected mice EGFP autofluorescence is also seen. Arrowheads are pointing to indicative CD+ cells. Data were compared using One-way ANOVA with Tukey's Multiple Comparison Test followed by Bonferroni correction. Significance level of all comparisons, $P<0.05$. Scale bars: 20 μm.

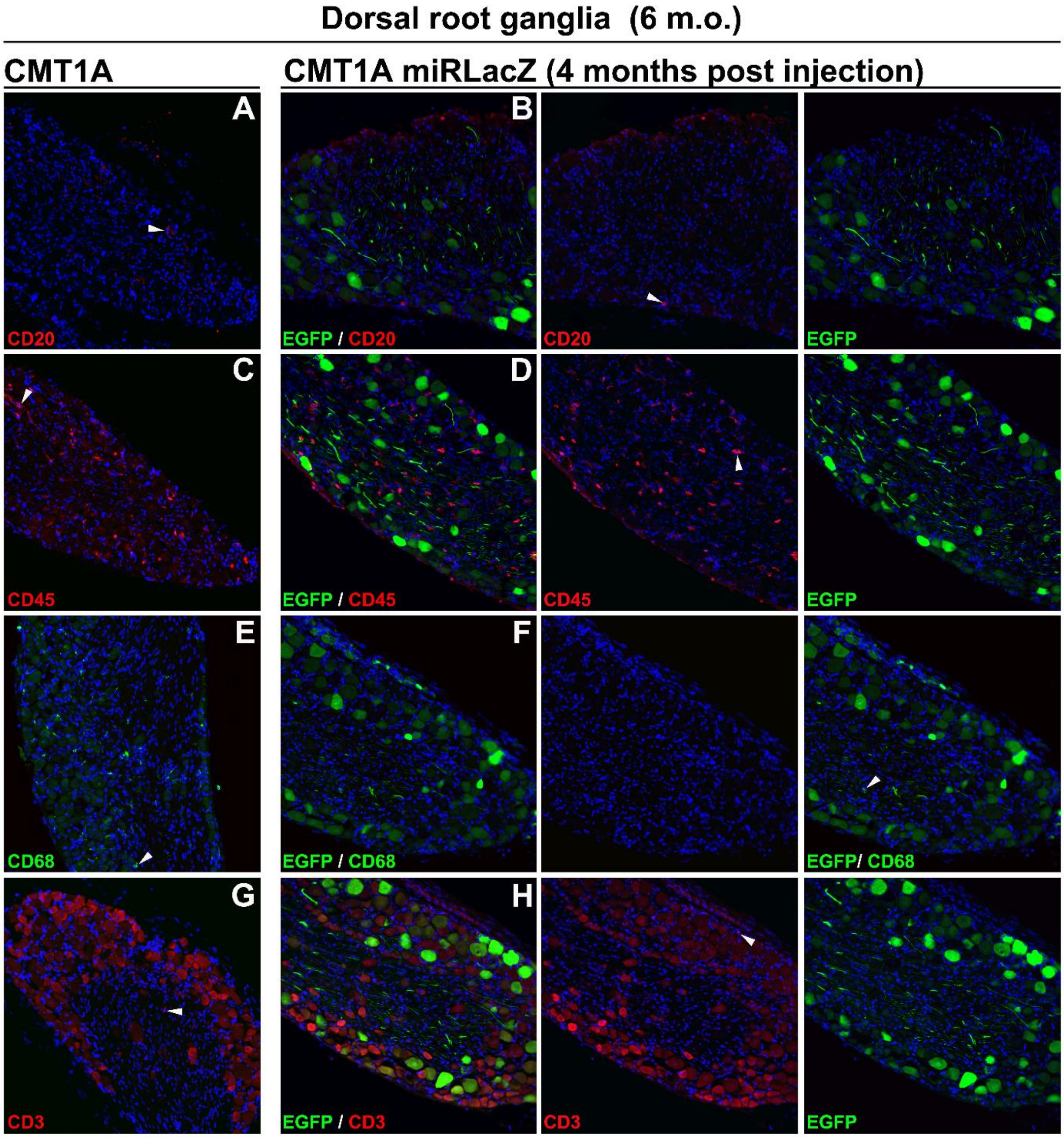

Fig. S11. Lack of inflammatory reaction in the dorsal root ganglia (DRGs) of AAV9-miRLacZ-injected CMT1A mice at 4 months post injection (6 months of age). Representative images of DRGs sections from CMT1A mice injected with AAV9-miRLacZ at 4 months post injection compared to age-matched non-injected CMT1A mice, immunostained for various inflammatory cell markers, as indicated **(A-G)**. Arrowheads are pointing on cells positive for the B-cell marker CD20 (red) **(A, B)**, leukocyte marker CD45 (red) **(C, D)**, macrophage marker CD68 (green) **(E, F),** or T-cell marker CD3 (red) **(G, H)**. Nuclear staining with DAPI (blue). In tissues of AAV9-miRLacZ injected mice EGFP green autofluorescence is also visible, including several DRG neuron cell bodies.

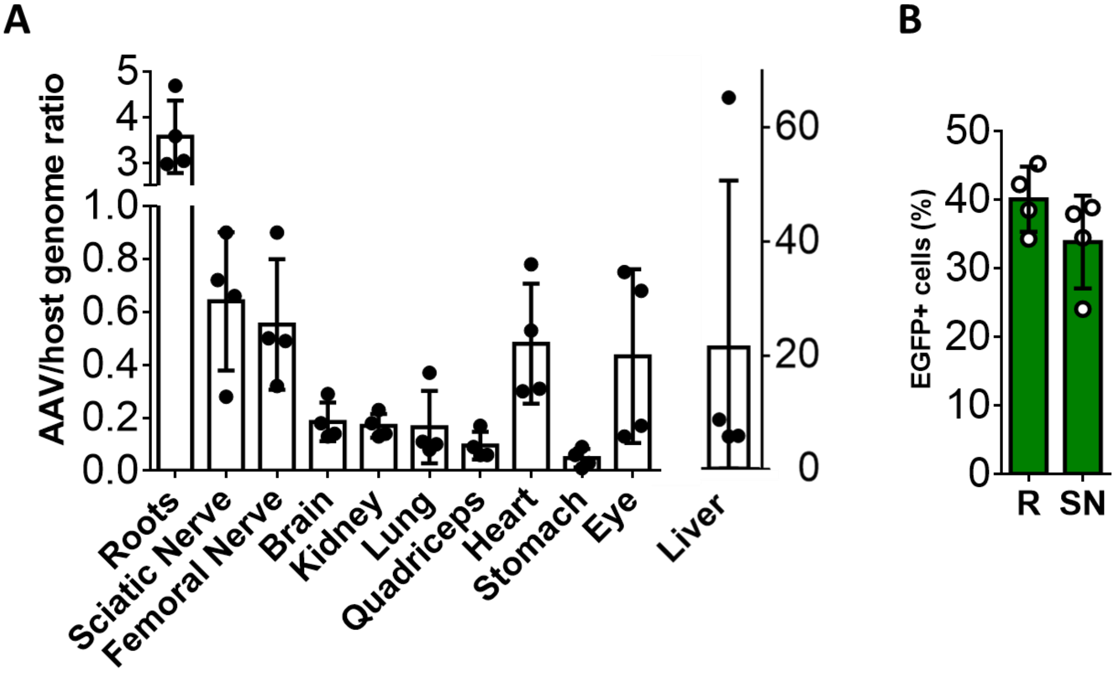

Fig. S12. Biodistribution analysis of early treated CMT1A mice. **(A)** Results of VGCN measurements in extracted DNA from PNS and non-PNS tissues of early treated CMT1A mice injected with AAV9-miR871 at 4 months post injection (mice 6 months old). Data were compared using One-way ANOVA with Tukey's Multiple Comparison Test. **(B)** Quantification of the percentage of EGFP positive cells in lumbar spinal roots and sciatic nerves of early treated CMT1A mice at 4 months post injection. Data were compared using unpaired t-test. There was no statistically significant difference among the groups. Significance level of all comparisons, $P<0.05$ (Mean, SD).

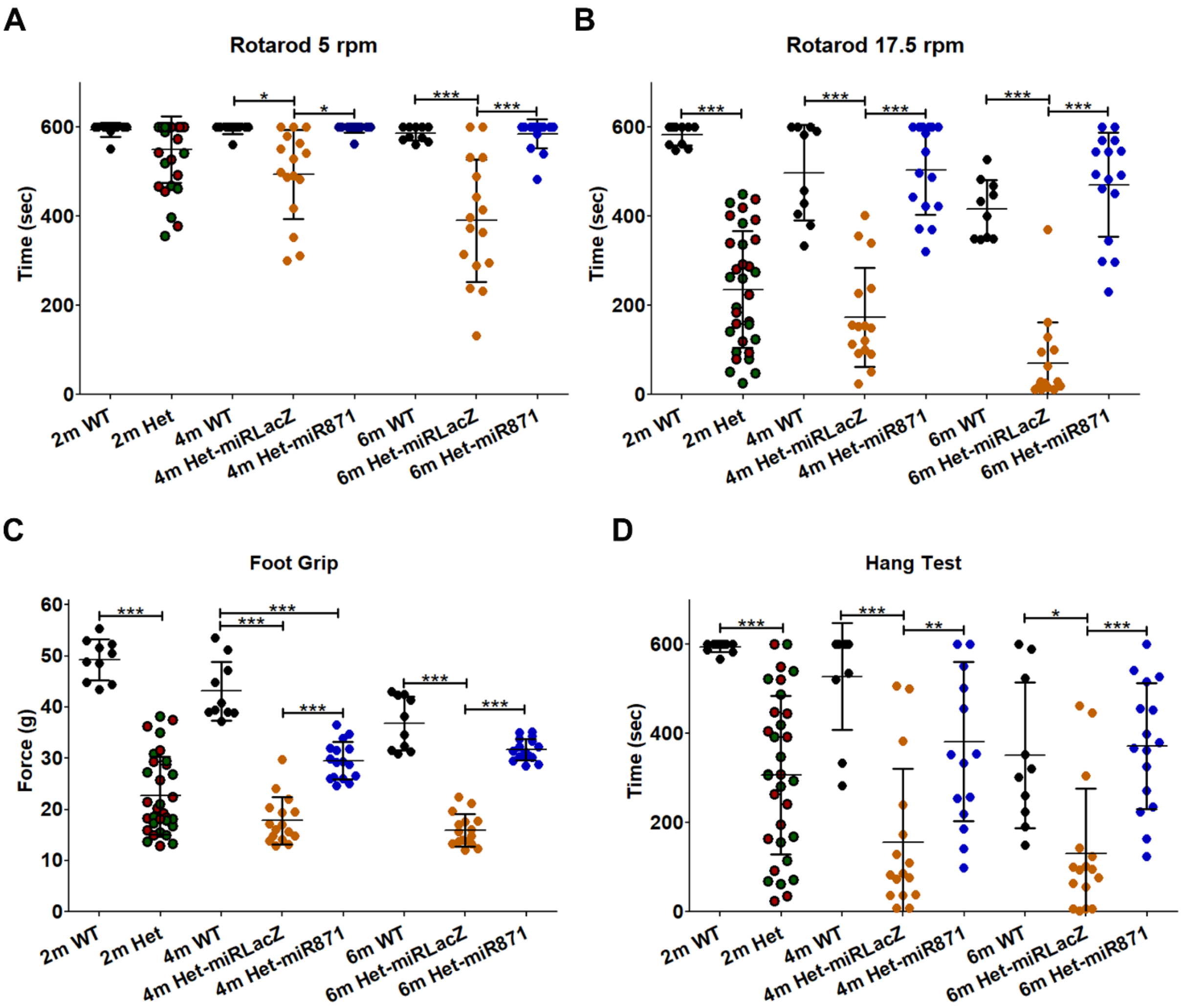

Fig. S13. Improved motor performance in early treated CMT1A mice. Results of behavioral analysis of CMT1A mice injected with AAV9-miR871 compared with age-matched WT mice and CMT1A mice injected with AAV9-miRLacZ controls with rotarod 5 rpm **(A)** and 17.5 rpm **(B)**, grip strength **(C)** and hang test **(D)** at 2 (before injections), 4, and 6 months of age (WT: n=10, CMT1A AAV9 –miR871 –miRLacZ n=16/group), as indicated. Values represent mean ± SD. Data were compared using One-way ANOVA with Tukey's Multiple Comparison Test. Significance level of all comparisons, P<0.05.

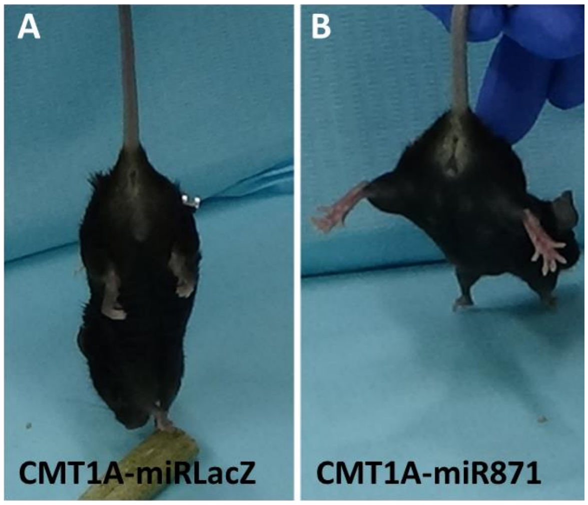

Fig S14. Improvement of hindlimb clasping phenotype in early treated CMT1A mice. Representative images of CMT1A mice injected at 2 months of age with either AAV9-miRLacZ or AAV9-miR871, examined at 4 months post-injection (6 months of age) as indicated, shows improvement of the hindlimb clasping phenotype in the treated animal.

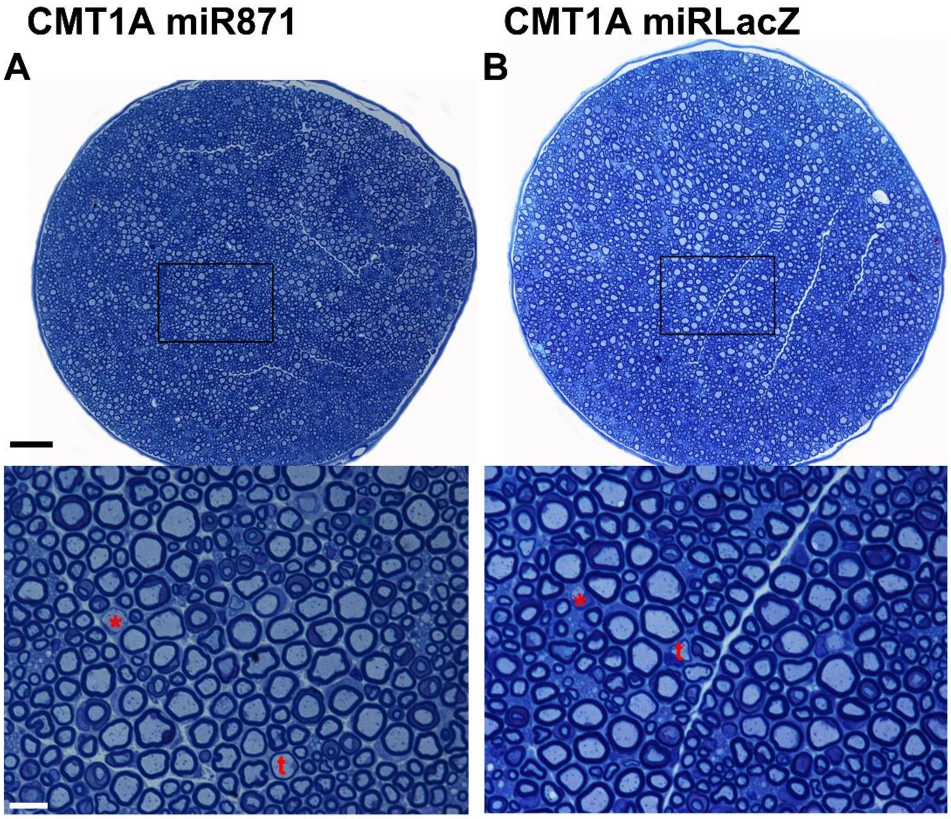

Fig. S15. Representative images of toluidine blue stained semithin section of sciatic nerves of early treated CMT1A mice. Low (upper panels) and higher (lower panels) magnification images of toluidine

blue stained semithin section of sciatic nerve of early treated CMT1A mice injected with either AAV9-miRLacZ or AAV9-miR871 examined at 4 months post-injection (6 months of age.) as indicated. Thinly (t) and demyelinated (*) fibers are indicated. Scale bars: 40 μm, for magnified: 10 μm.

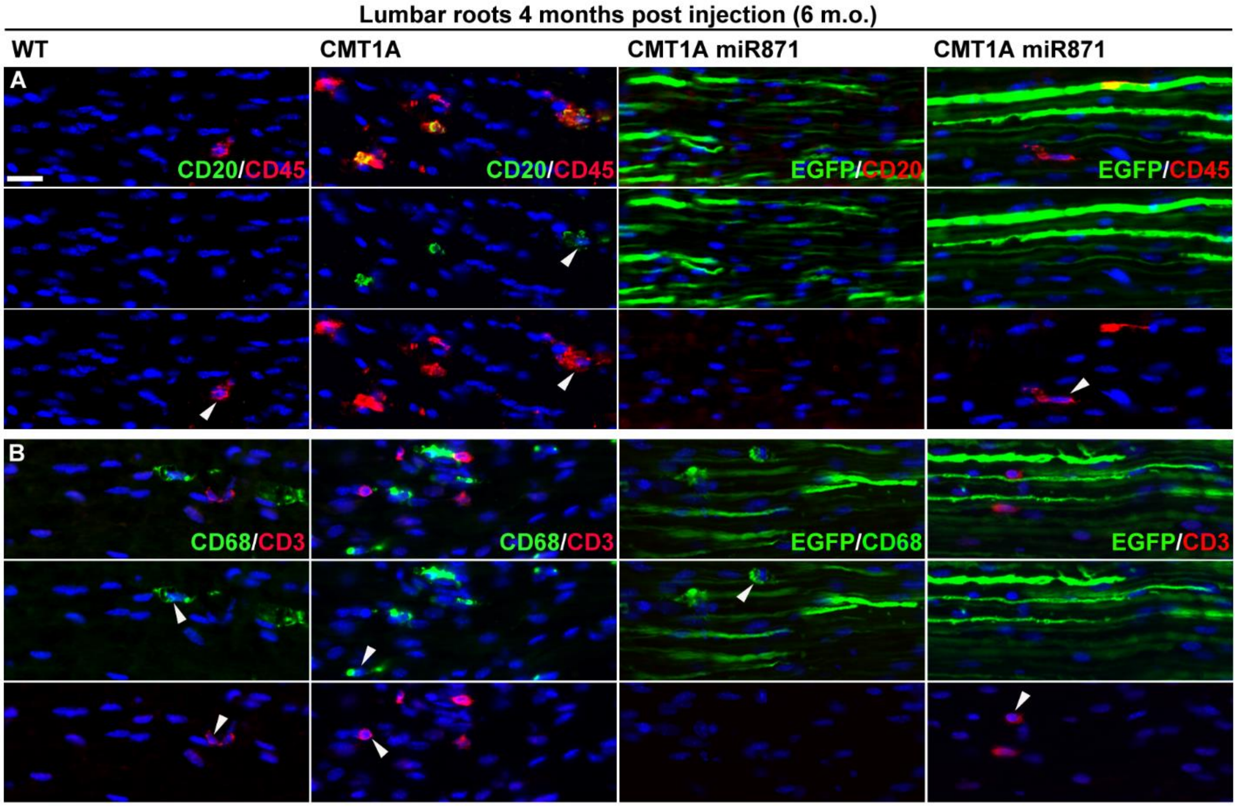

Fig. S16. Improvement of inflammatory cell infiltrates in lumbar spinal roots of early treated CMT1A mice. Representative images of lumbar spinal root sections of 6 m.o. CMT1A mice 4 months post injection with AAV9-miR871 (n=4) compared to age-matched non-injected WT (n=4) and CMT1A mice (n=4) **(A-B)**, as indicated. Tissues were immunostained against B-cell marker CD20 (green or red in tissues of injected animals as indicated), leukocyte marker CD45 (red), macrophage marker CD68 (green) and T-cell marker CD3 (red), and counterstained with DAPI (blue). In tissues of AAV9-miR871 injected mice EGFP autofluorescence is also seen. Arrowheads are pointing to indicative CD+ cells. Scale bar: 20 μm. Immunostaining images of 6-month-old non-injected CMT1A and CMT1A-miR871 mice are also shown in Figure 5.

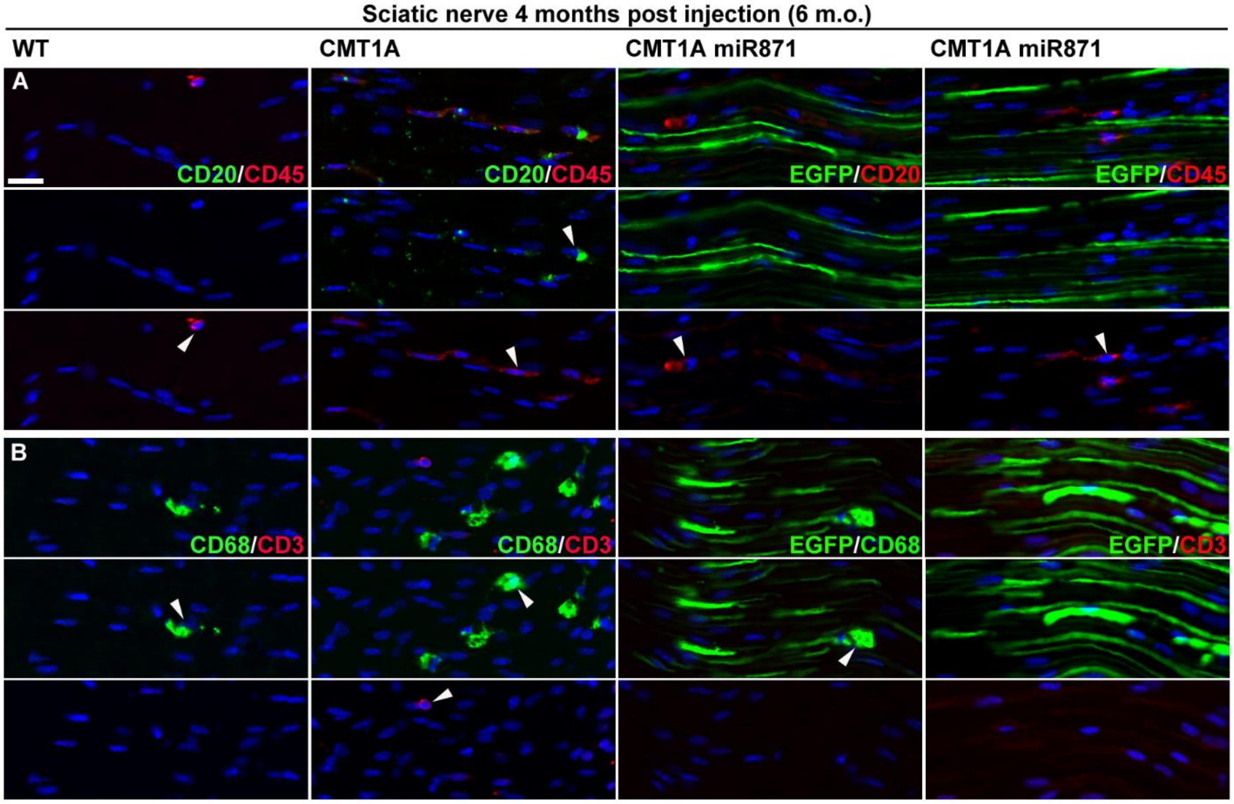

Fig. S17. Improvement of inflammatory cell infiltrates in sciatic nerves of early treated CMT1A mice. Representative images of sciatic nerve sections of 6 m.o. CMT1A mice 4 months post injection with AAV9-miR871 (n=4) compared to age-matched non-injected WT (n=4) and CMT1A mice (n=4) **(A-B)**, as indicated. Tissues were immunostained against B-cell marker CD20 (green or red in tissues of injected animals as indicated), leukocyte marker CD45 (red), macrophage marker CD68 (green) and T-cell marker CD3 (red), and counterstained with DAPI (blue). In tissues of AAV9-miR871 injected mice EGFP autofluorescence is also seen. Arrowheads are pointing to indicative CD+ cells. Scale bar: 20 μm.

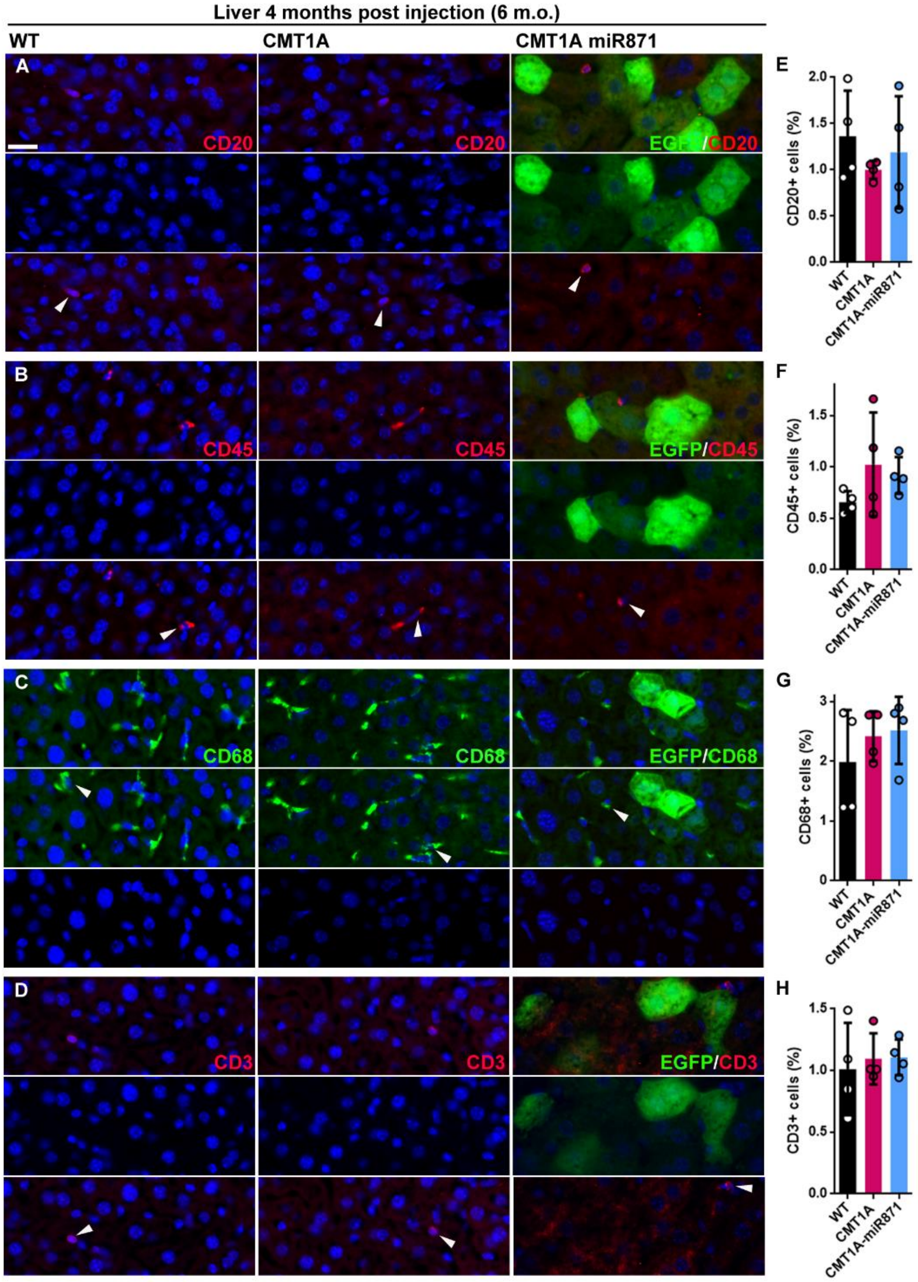

Fig. S18. Lack of any inflammatory response in the liver of AAV9-miR871 early treated CMT1A mice 4 months post injection (6 months of age). Representative images of immunostained liver sections from CMT1A mice injected with AAV9-miR871 (n=4) at 4 months post injection compared to age-matched non-injected WT (n=4) and CMT1A mice (n=4) **(A-D)**, as indicated. Quantification of the percentage of cells positive for the B-cell marker CD20 (red) **(E)**, leukocyte marker CD45 (red) **(F),** macrophage marker CD68 (green) (**G),** or T-cell marker CD3 (red) (**H**) was calculated in relation to the total cell number (Mean, SD). Cell nuclei were counterstained with DAPI (blue). In tissues of AAV9-miR871 injected mice EGFP autofluorescence in several hepatocytes is also seen. Arrowheads are pointing to indicative CD+ cells. Data

were compared using One-way ANOVA with Tukey's Multiple Comparison Test. Significance level of all comparisons, P<0.05. Scale bar: 20 μm.

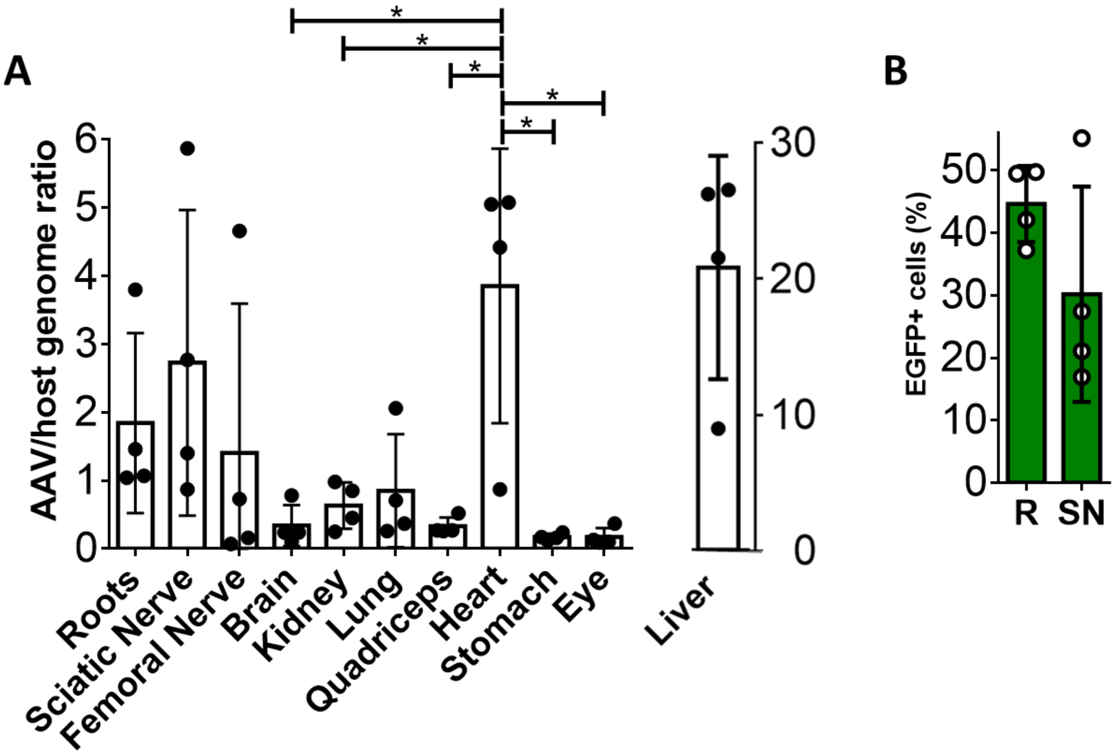

Fig. S19. Biodistribution analysis of late treated CMT1A mice. **(A)** Results of VGCN measurements in extracted DNA from PNS and non-PNS tissues of late treated CMT1A mice injected with AAV9-miR871 at 4 months post injection (mice 10 months old). Data (excluding the liver) were compared using One-way ANOVA with Tukey's Multiple Comparison Test. **(B)** Quantification of the percentage of EGFP positive cells in lumbar spinal roots and sciatic nerves of late treated CMT1A mice at 4 months post injection. Data were compared using unpaired t-test. Significance level of all comparisons, P<0.05 (Mean, SD).

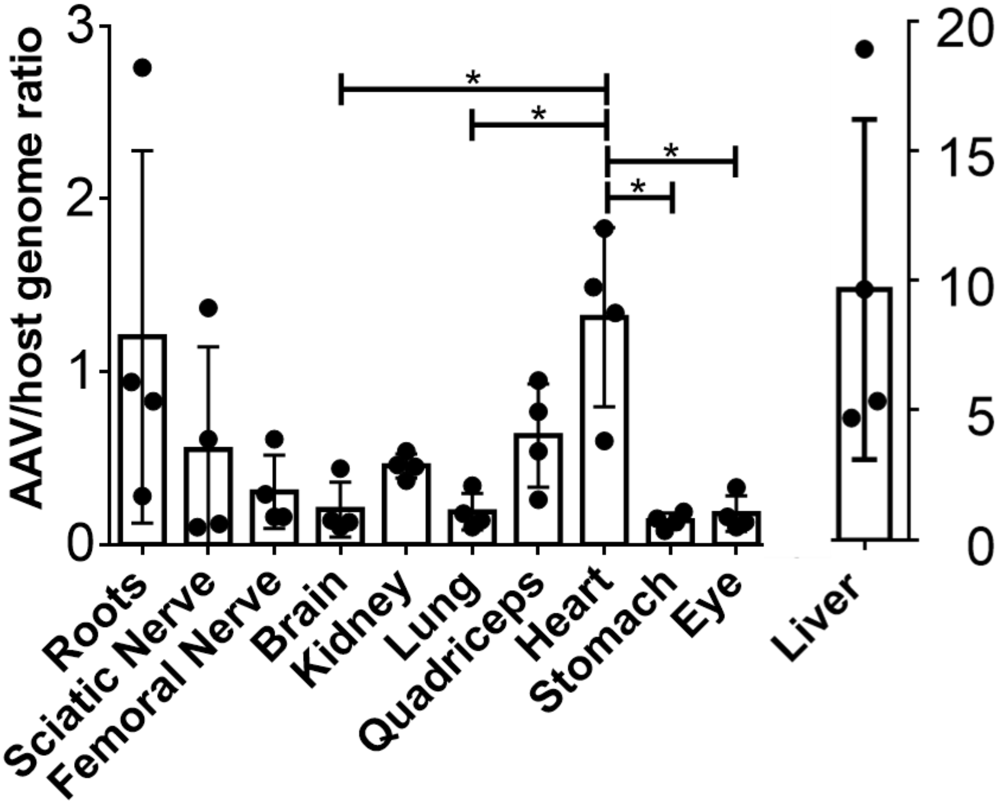

Fig. S20. Biodistribution analysis of extended early treated CMT1A mice. Results of VGCN measurements in extracted DNA from PNS and non-PNS tissues of extended early treated CMT1A mice injected with AAV9-miR871 at 2 months of age and examined 8 months post injection (10 months of age). Data (excluding the liver) were compared using One-way ANOVA with Tukey's Multiple Comparison Test. Significance level of all comparisons, P<0.05 (Mean, SD).

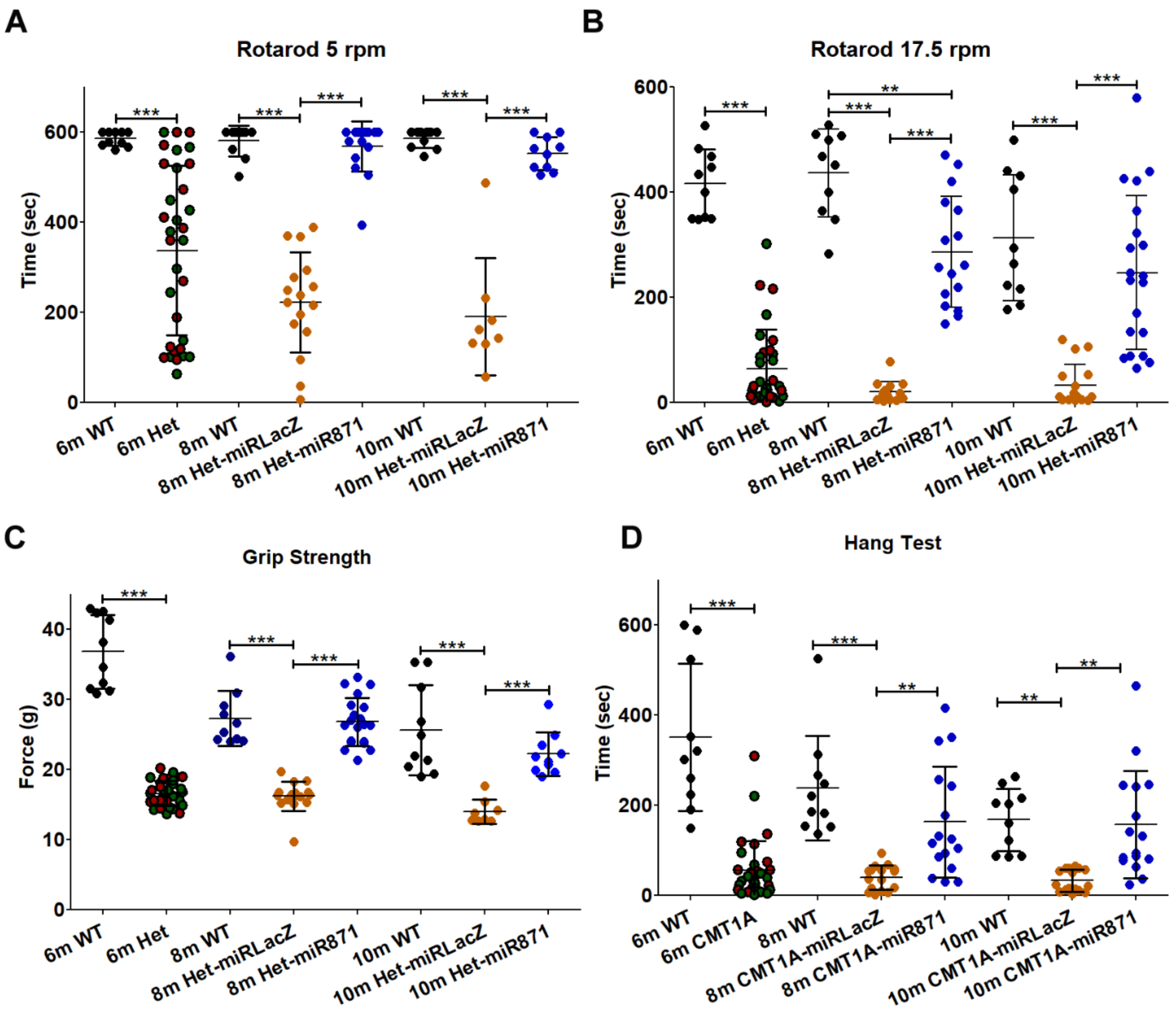

Fig. S21. Improved motor performance in late treated CMT1A mice. Results of behavioral analysis of CMT1A mice injected with AAV9-miR871 compared with age-matched WT mice and CMT1A mice injected with AAV9-miRLacZ controls with rotarod 5 rpm **(A)** and 17.5 rpm **(B)**, grip strength **(C)** and hang test **(D)** at 6 (before injections), 8, and 10 months of age (WT: n=10, CMT1A AAV9 –miR871 –miRLacZ n=16/group), as indicated. Values represent mean ± SD. Data were compared using One-way ANOVA with Tukey's Multiple Comparison Test. Significance level of all comparisons, P<0.05.

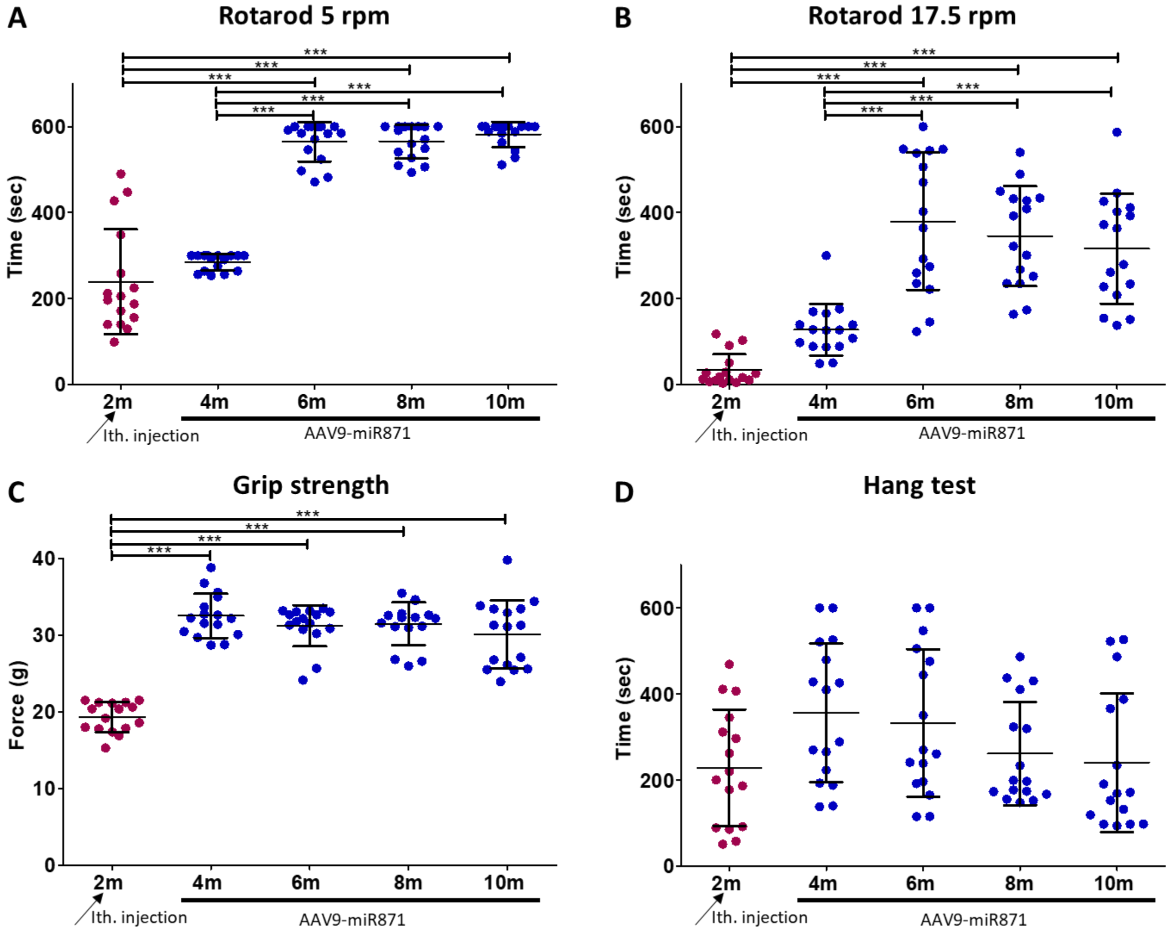

Fig. S22. Motor performance of extended early treated CMT1A mice. Results of behavioral analysis of CMT1A mice injected with AAV9-miR871 compared with age-matched WT mice and CMT1A mice injected with AAV9-miRLacZ controls with rotarod 5 rpm **(A)** and 17.5 rpm **(B)**, grip strength **(C)** and hang test **(D)** at 2 (before injections), 4, 6, 8, and 10 months of age (WT: n=10, CMT1A AAV9 –miR871 –miRLacZ n=16/group), as indicated. Values represent mean ± SD. Data were compared using One-way ANOVA with Tukey's Multiple Comparison Test. Significance level of all comparisons, P<0.05.

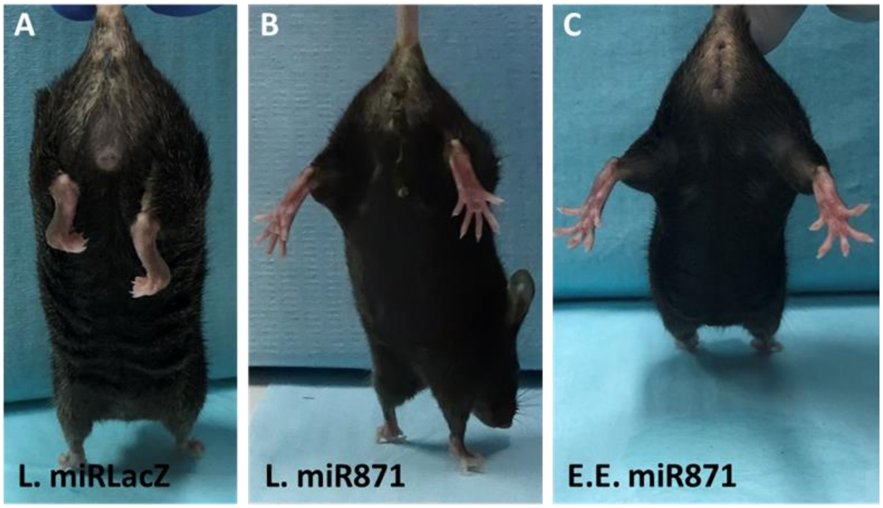

Fig. S23. Improvement of hindlimb clasping phenotype in late and extended early treated CMT1A mice. Representative images of CMT1A mice injected at 6 or 2 months of age with either AAV9-miRLacZ or AAV9-miR871, examined at 4 months post-injection (6 months old) or 8 months post-injection (10 months old), shows improvement of the hindlimb clasping phenotype in the treated animals.

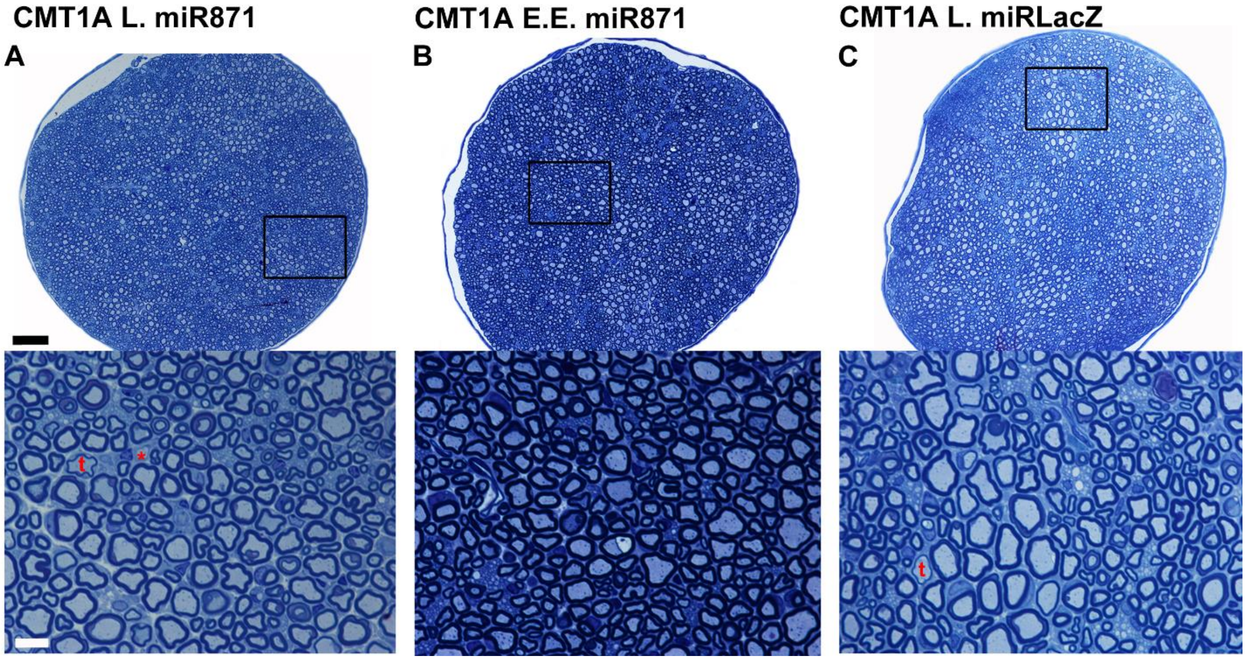

Fig. S24. Representative images of toluidine blue stained semithin section of sciatic nerves of late and extended early treated CMT1A mice. Low (upper panels) and higher (lower panels) magnification images of toluidine blue stained semithin section of sciatic nerve of late (L) and extended early (E.E.) treated CMT1A mice injected with either AAV9-miRLacZ or AAV9-miR871 examined at 4 or 8 months post-injection (10 months old). Thinly (t) and demyelinated (*) fibers are indicated. Scale bars: 40 μm, for magnified: 10 μm.

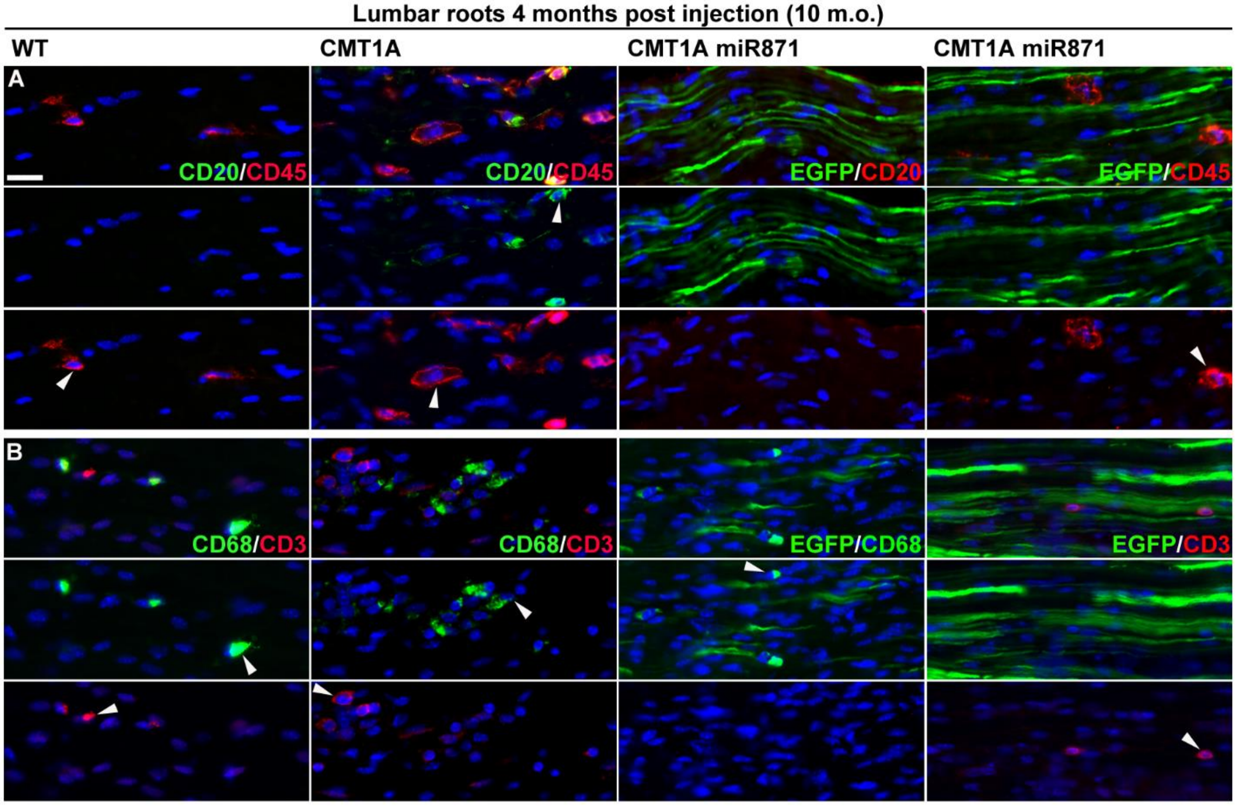

Fig. S25. Improvement of inflammatory cell infiltrates in lumbar spinal roots of late treated CMT1A mice. Representative images of lumbar spinal root sections of 10-month-oldCMT1A mice 4 months post injection with AAV9-miR871 (n=4) compared to age-matched non-injected WT (n=4) and CMT1A mice (n=4) **(A-B)**, as indicated. Tissues were immunostained against B-cell marker CD20 (green or red in tissues of injected animals as indicated), leukocyte marker CD45 (red), macrophage marker CD68 (green) and T-cell marker CD3 (red), and counterstained with DAPI (blue). Arrowheads are pointing to indicative CD+ cells. In tissues of AAV9-miR871 injected mice EGFP autofluorescence is also seen. Scale bar: 20 μm (Immunostaining images of 10-month-old non-injected CMT1A and CMT1A-miR871 mice are also used in Figure 8).

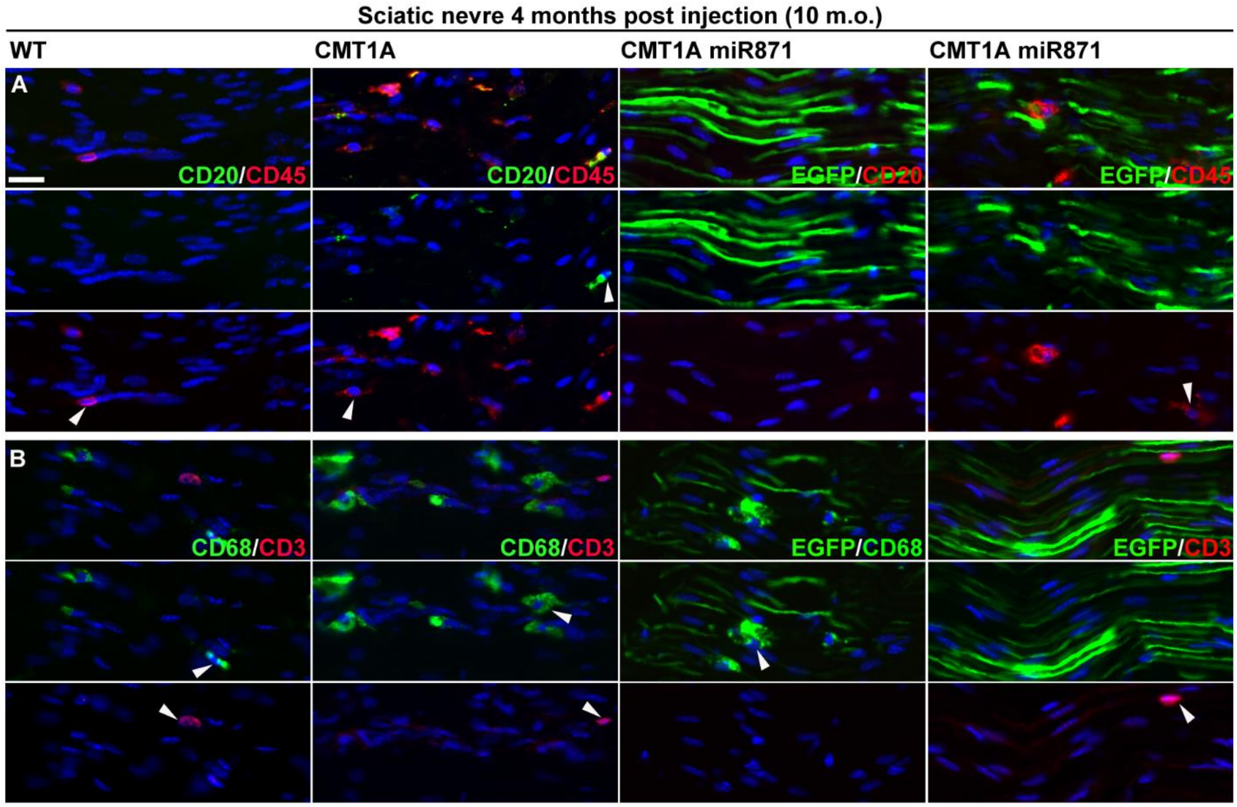

Fig. S26. Improvement of inflammatory cell infiltrates in sciatic nerves of late treated CMT1A mice. Representative images of sciatic nerve sections of 10-month-old CMT1A mice 4 months post injection with AAV9-miR871 (n=4) compared to age-matched non-injected WT (n=4) and CMT1A mice (n=4) **(A-B)**, as indicated. Tissues were immunostained against B-cell marker CD20 (green or red in tissues of injected animals as indicated), leukocyte marker CD45 (red), macrophage marker CD68 (green) and T-cell marker CD3 (red), and counterstained with DAPI (blue). Arrowheads are pointing to indicative CD+ cells. In tissues of AAV9-miR871 injected mice EGFP autofluorescence is also seen. Scale bar: 20 μm.

SUPPLEMENTARY DATA

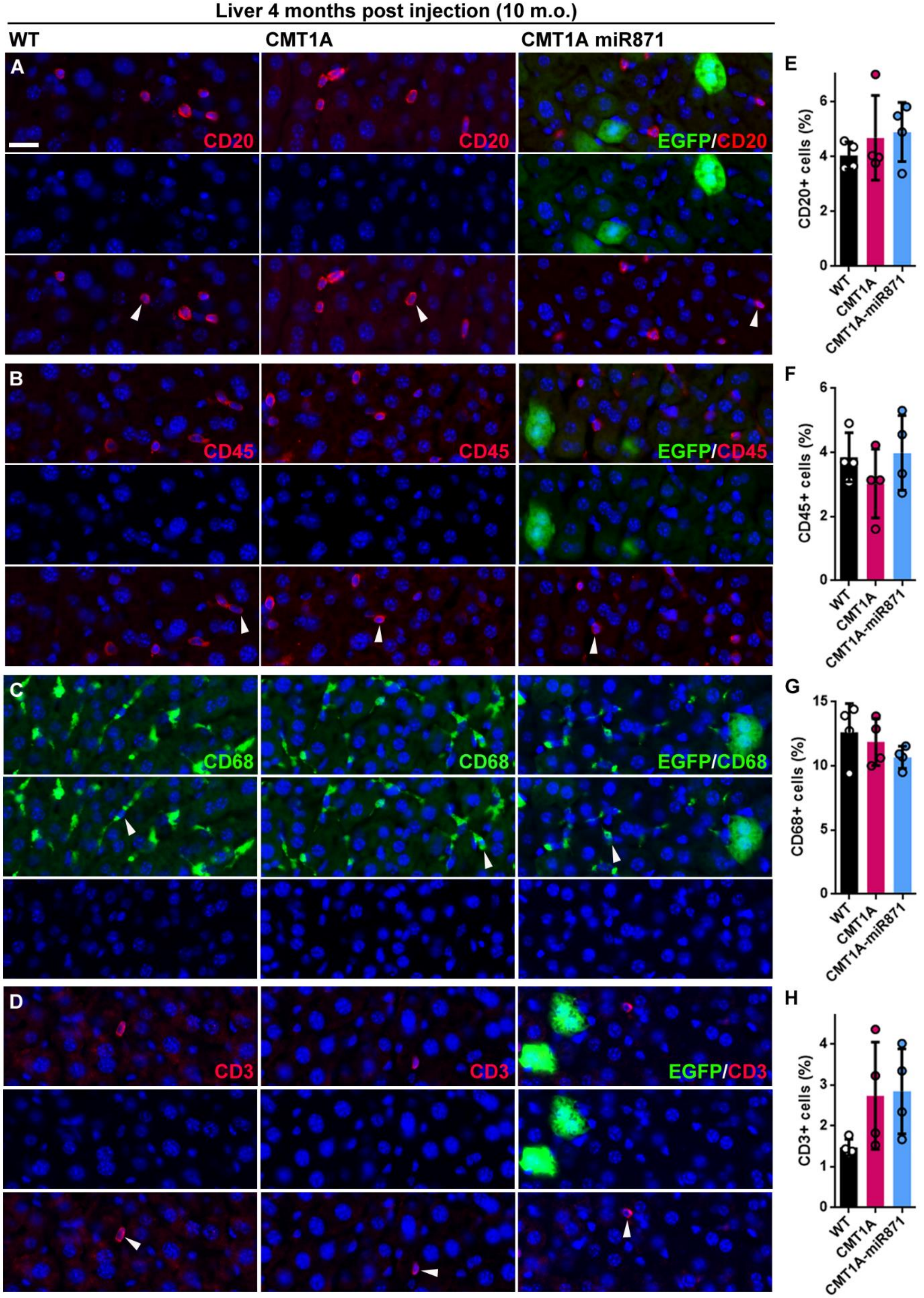

Fig. S27. Lack of any inflammatory response in the liver of AAV9-miR871 late treated CMT1A mice 4 months post injection (10 months old.). Representative images of immunostained liver sections from CMT1A mice injected with AAV9-miR871 (n=4) at 4 months post injection compared to age-matched non-injected WT (n=4) and CMT1A mice (n=4) **(A-D)**, as indicated. Quantification of the percentage of cells positive for the B-cell marker CD20 (red) (**E**), leukocyte marker CD45 (red) (**F**), macrophage marker CD68 (green) (**G**), or T-cell marker CD3 (red) **(H)** was calculated in relation to the total cell number (Mean, SD). Cell nuclei were counterstained with DAPI (blue). In tissues of AAV9-miR871 injected mice EGFP autofluorescence in hepatocytes is also seen. Arrowheads are pointing to indicative CD+ cells. Data were compared using One-way ANOVA with Tukey's Multiple Comparison Test. Significance level of all comparisons, $P<0.05$. Scale bar: 20 μm.

Effects of AAV9-miR871 on WT mice

To assess potential toxic effects of *Pmp22* over-silencing, we injected the AAV9-miR871 and -miRLacZ vectors into 2-months-old WT mice, expressing only normal levels of mu*Pmp22,* and evaluated effects 6 weeks later with western blot, and then 4 months post-injection with real-time PCR, western blot analysis, behavioral testing, NF-L testing, electrophysiological examination, and morphometric observation (**Fig. S28**). WT mice injected with AAV9-miR871 showed reduced of Pmp22 protein levels in spinal roots, sciatic and femoral nerves. In addition, MPZ protein levels were reduced in sciatic nerves at 6-weeks and 4 months post-injection, suggesting abnormal myelination (**Fig. S29**). *Pmp22* mRNA showed variable reduction in AAV9-miR871-treated WT mice 4 months after injection that reached significance only in spinal roots. A similar trend was observed for mu*Mpz* levels. mu*Gjb1* transcripts increased in AAV9-miR871-treated WT sciatic and femoral nerves (**Fig. S30 and table S4**). We assessed motor performance in all mice groups before injection and until the end of the observation period using rotarod at 5 and 17.5 rpm, grip strength, and hang test analyses (**Fig. S31**). AAV9-miR871-treated WT mice showed transiently impaired rotarod performance (at both speeds) at 2 months post-injection with recovery in function 2 months later (4 months post-injection). They also showed attenuated grip strength performance at all-time points and worsening in hang test performance at 4 months post injection. However, they did not present hindlimb clasping phenotype (**Fig. S32**) or MNCV deficits 4 months after vector injection (**Fig. S33**) but CMAP scores were significantly reduced (AAV9-miR871: 4.62±1.22, AAV9-miRLacZ: 7.03±0.72, WT: 6.89±1.76). Interestingly, NF-L plasma levels were not elevated (**Fig. S34**) and morphological analysis did not reveal any apparent morphological defects (**Fig. S35**) in AAV9-miR871 injected WT mice.

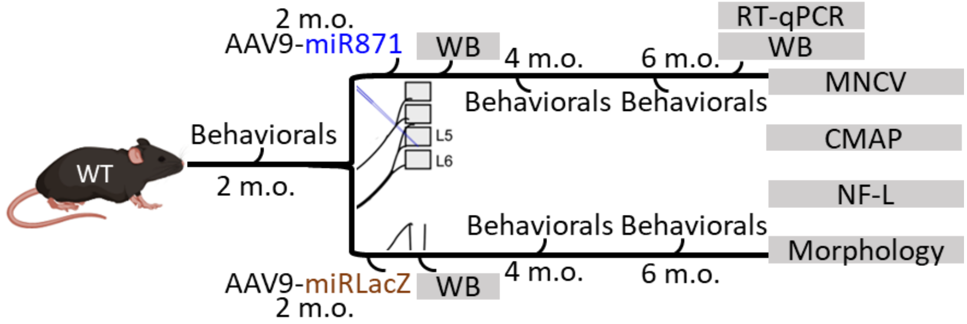

Fig. S28. Diagram showing the study design of vector injections into WT mice and the timing of functional, morphological and molecular analysis.

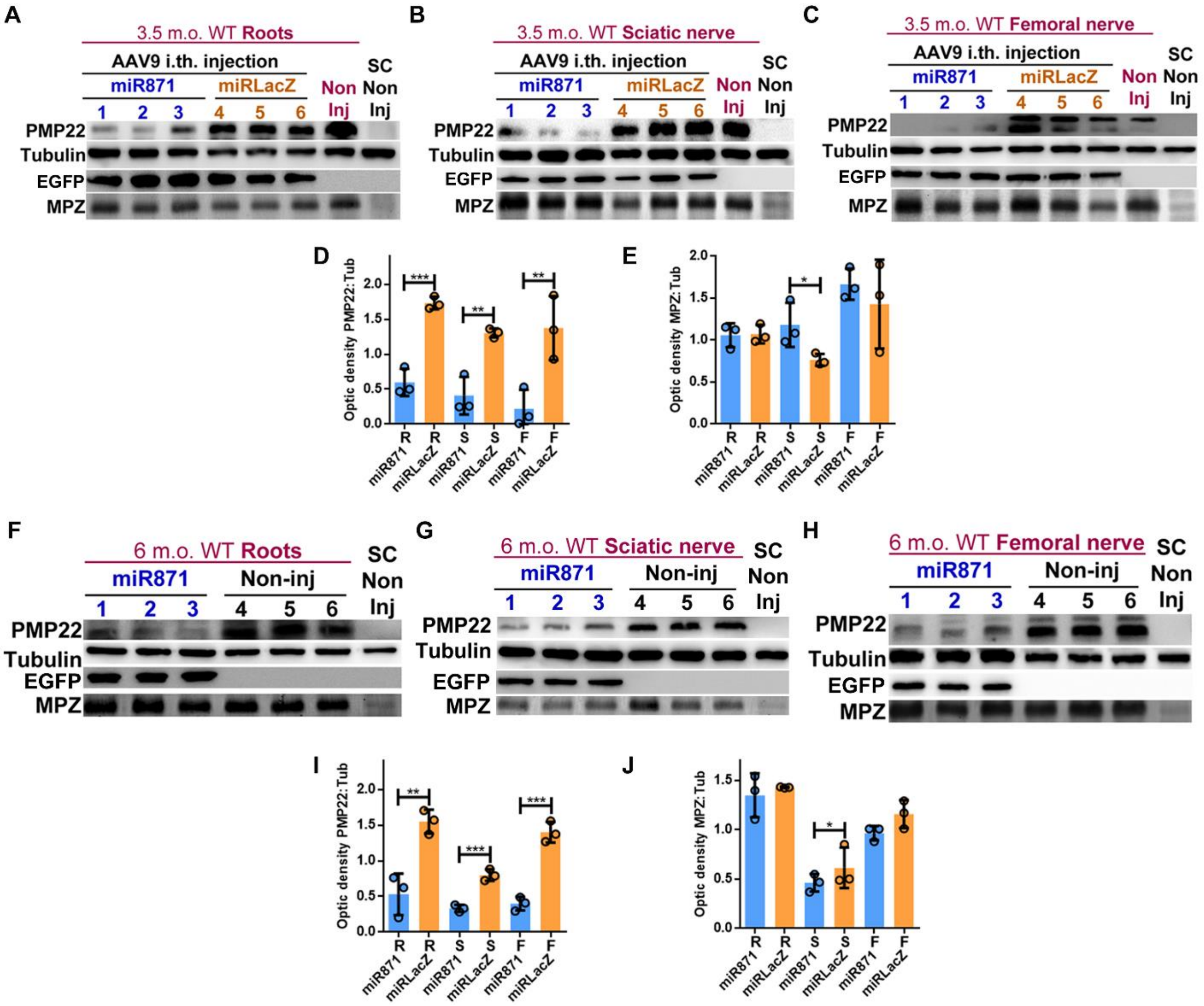

Fig. S29. Western blot analysis of WT mice injected at 2 months of age with AAV9-miR871 or -miRLacZ and analyzed at 6-weeks (3.5 months of age) or 4 months post injection (6 months of age) showing reduction of muPMP22. Representative western blot images showing the muPMP22,

muTubulin, EGFP and muMPZ protein levels and quantification in PNS tissue samples as indicated from WT mice injected with AAV9-miR871 roots, sciatic nerves and femoral nerves at 6-weeks **(A-E)** or 4-months **(F-J)** post injection. At 6-weeks post-injection, protein levels in PNS tissue samples from WT mice injected with AAV9-miR871 were compared to the analogous protein levels of WT mice injected with AAV9-miRLacZ. At 4-months post-injection, protein levels in PNS tissue samples from WT mice injected with AAV9-miR871 were compared to those of aged-matched non-injected WT mice. Quantification of muPMP22 and muMPZ **(D-E, I-J)** levels was performed according to optic density levels normalized to tubulin.

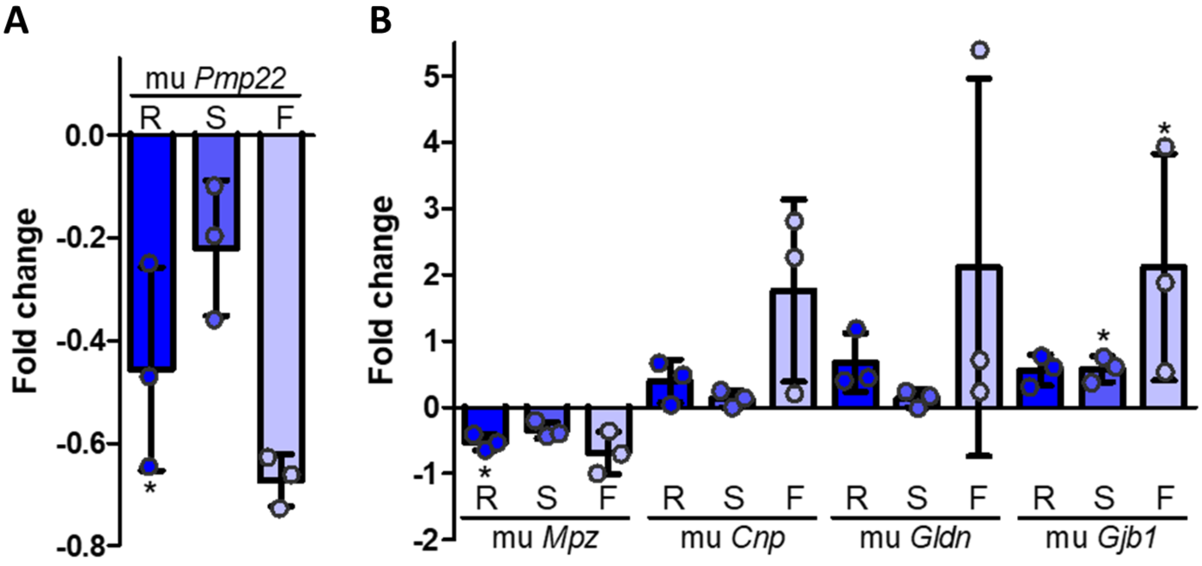

Fig. S30. Real-time PCR analysis of WT mice injected with AAV9-miR871 at 4-months post injection (6 months old). Quantitative real-time PCR analysis of mu*Pmp22* (**A**), as well as of mu*Mpz*, mu*Cnp*, mu*Gldn* and mu*Gjb1* (**B**) gene expression analysis in lumbar roots, sciatic and femoral nerves at 4 months post-injection (n=3). Fold relative mRNA expression levels of AAV9-miR871 injected WT mice were calculated compared to non-injected WT mice. All samples were normalized to endogenous control *Gapdh*.

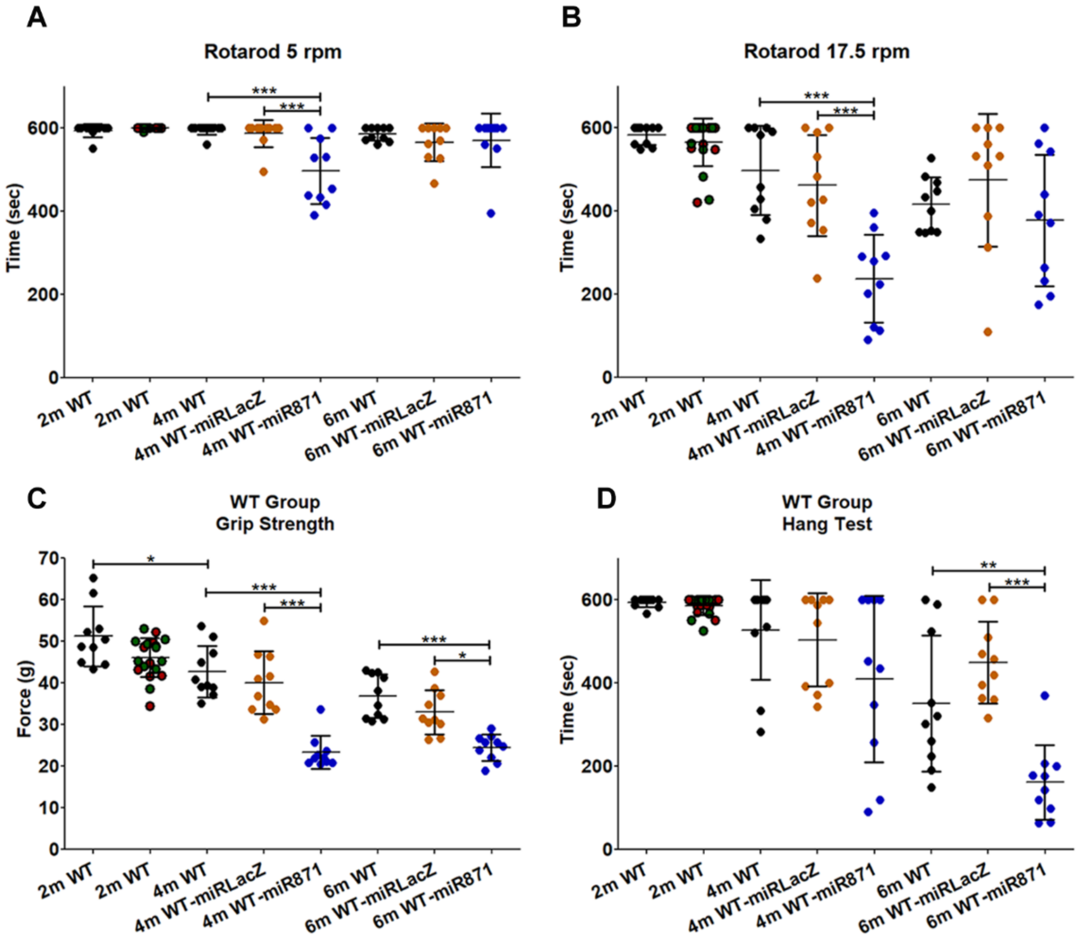

Fig. S31. Partially impaired motor performance in AAV9-miR871 injected WT mice. Results of behavioral analysis of WT mice injected with AAV9-miR871 compared with age-matched WT mice injected with AAV9-miRLacZ controls with rotarod at 5 rpm **(A)** and 17.5 rpm **(B)**, grip strength **(C)** and hang test **(D)** at 2 (before injections), 4, and 6 months of age (Non-injected WT: n=10, WT AAV9-miR871 or -miRLacZ: n=16/group) as indicated. Values represent mean ± SD. Data were compared using One-way ANOVA with Tukey's Multiple Comparison Test. Significance level of all comparisons, P<0.05.

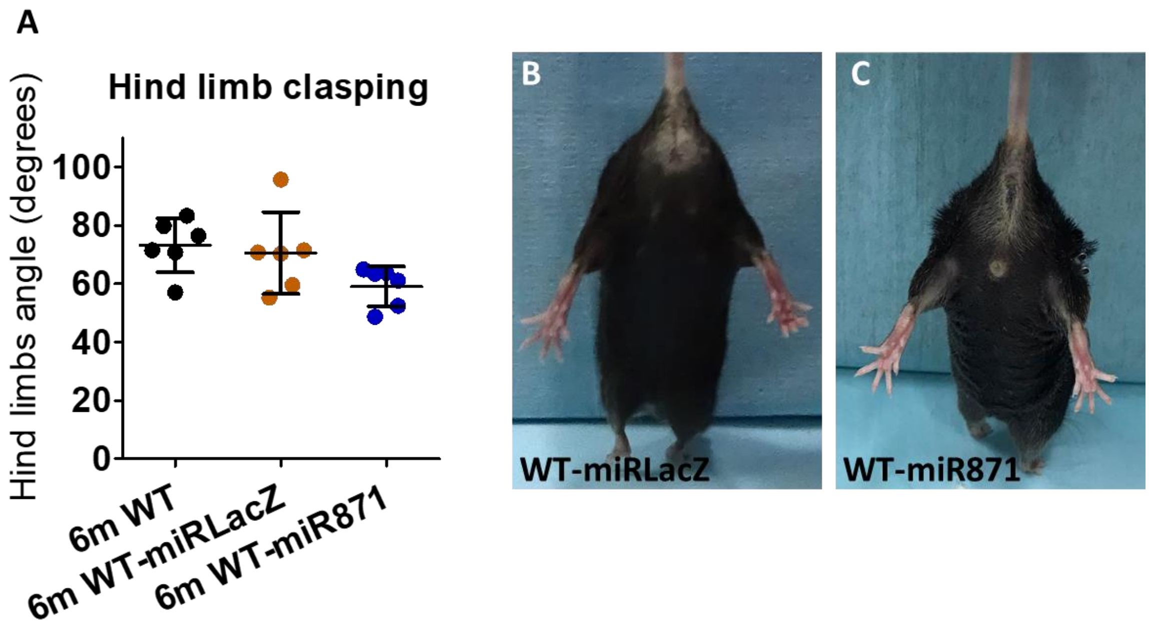

Fig. S32. Absence of hindlimb clasping phenotype in AAV9-miR871 injected WT mice. (A) Hindlimbs opening angle estimation and representative images **(B)** at the final time point comparing age-matched non-injected and injected WT mice with either AAV9-miRLacZ or AAV9-miR871, as indicated (n=6/group). Values represent mean ± SD. Data were compared using One-way ANOVA with Tukey's Multiple Comparison Test. Significance level of all comparisons, P<0.05.

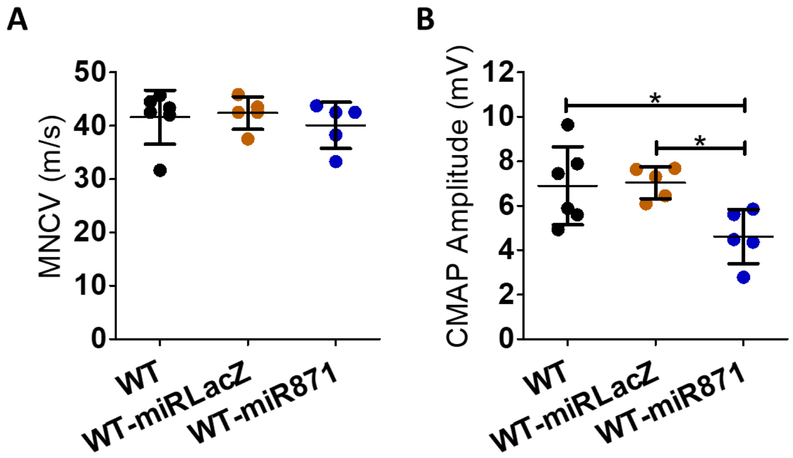

Fig. S33. Electrophysiological evaluation of WT mice injected with either AAV9-miR871 or AAV9-miRLacZ showing unchanged sciatic MNCV but reduced CMAP amplitudes. MNCV (**A**) and CMAP (**B**) analysis was performed in WT mice injected with either AAV9-miR871 or -miRLacZ at 4 months post-injection (6 months of age) (Non-injected WT: n=6, WT injected with AAV9-miR871 or -miRLacZ: n=5). Values represent mean ± SD. Data were compared using One-way ANOVA with Tukey's Multiple Comparison Test. Significance level of all comparisons, P<0.05.

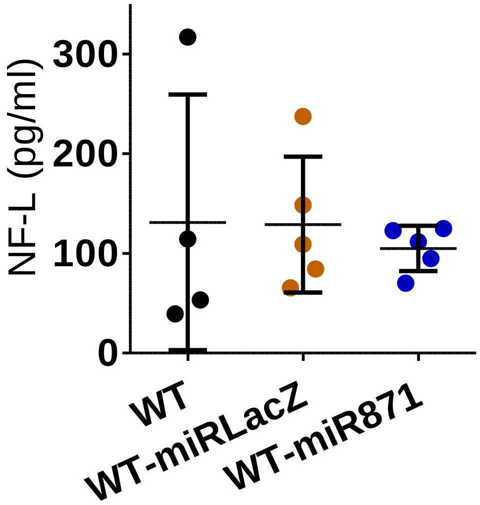

Fig. S34. Plasma NF-L analysis in of WT mice injected with either AAV9-miR871 or AAV9-miRLacZ showing no change after injections. Plasma NF-L analysis was performed in WT mice injected with either AAV9-miR871 or -miRLacZ at 4 months post-injection (6 months of age) (Non-injected WT: n=4, WT injected with AAV9-miR871 or -miRLacZ: n=5). Values represent mean ± SD. Data were compared using One-way ANOVA with Tukey's Multiple Comparison Test. Significance level of all comparisons, P<0.05.

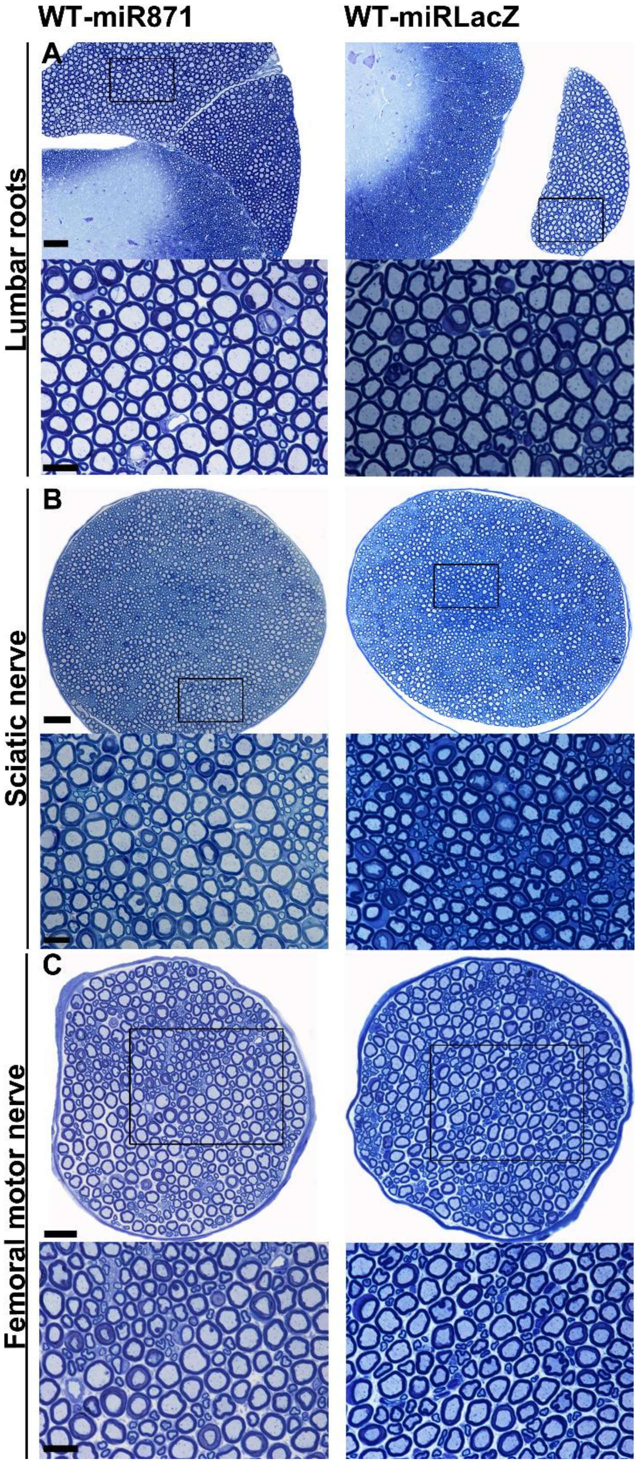

Fig. S35. Representative images of toluidine blue stained semithin sections of PNS tissues of WT mice injected with either AAV9-miR871 or AAV9-miRLacZ showing no apparent morphological defects. Toluidine blue stained semithin sections of lumbar roots **(A)**, sciatic **(B)** and femoral motor **(C)** nerves of WT mice injected with either AAV9-miRLacZ or AAV9-miR871 and examined 4 months post-injection (at 6 months of age). Scale bars: **(A)**: 50 μm, for magnified: 10 μm, **(B)**: 40 μm, for magnified: 10 μm, **(C)**: 20 μm, for magnified: 10 μm.

SUPPLEMENTARY DATA

CMT1A mice 6 w. post injection		Average fold change of:					
		hu *PMP22*	mu *Pmp22*	mu *Mpz*	mu *Cnp*	mu *Gldn*	mu *Gjb1*
Tissue	Roots	-0.30±0.12	-0.25±0.06	0.65±0.47	3.77±0.82	1.23±0.79	0.96±0.55
	Sciatic N.	-0.56±0.35	-0.58±0.32	2.16±0.49	5.62±2.34	2.28±0.49	2.88±0.40
	Femoral N.	-0.64±0.19	-0.61±0.25	2.34±1.59	2.14±1.52	1.98±1.30	4.46±2.91

Decrease	Increase	Non. Significant

Table S1: CMT1A mice mRNA transcripts fold change at 6 weeks post intrathecal injection of AAV9.miR871. Quantitative real-time PCR analysis of huPMP22 and mu*Pmp22* as well as of mu*Mpz*, mu*Cnp*, mu *Gldn* and mu*Gjb1* gene expression in lumbar roots, sciatic and femoral nerves at 6 weeks post-injection (n=3/group). Fold relative mRNA expression levels of AAV9-miR871 injected CMT1A mice were calculated compared to AAV9-miRLacZ injected CMT1A (Mean ± SD). All samples were normalized to endogenous control *Gapdh*.

CMT1A mice Early Treatment		Fold change of:					
		hu *PMP22*	mu *Pmp22*	mu *Mpz*	mu *Cnp*	mu *Gldn*	mu *Gjb1*
Tissue	Roots	-0.76±0.11	-0.86±0.03	2.52±1.78	1.19±0.05	1.44±0.62	2.39±1.30
	Sciatic N.	-0.41±0.19	-0.44±0.18	2.27±0.35	2.63±0.81	2.68±0.65	2.04±0.40
	Femoral N.	-0.89±0.06	-0.88±0.09	7.48±1.16	9.74±3.55	8.52±1.35	8.43±2.60

Decrease	Increase	Non. Significant

Table S2: Early treated CMT1A mice mRNA transcripts fold change at 4 months post intrathecal injection of AAV9.miR871. Quantitative real-time PCR analysis of huPMP22 and mu*Pmp22* as well as of mu*Mpz*, mu*Cnp*, mu *Gldn* and mu*Gjb1* gene expression in lumbar roots, sciatic and femoral nerves at 6 weeks post-injection (n=4/group). Fold relative mRNA expression levels of AAV9-miR871 injected CMT1A mice were calculated compared to AAV9-miRLacZ injected CMT1A (Mean ± SD). All samples were normalized to endogenous control *Gapdh*.

CMT1A mice Late Treatment		Fold change of:					
		hu *PMP22*	mu *Pmp22*	mu *Mpz*	mu *Cnp*	mu *Gldn*	mu *Gjb1*
Tissue	Roots	-0.49±0.11	-0.59±.21	1.04±1.02	1.82±0.87	1.23±0.67	1.17±0.69
	Sciatic N.	-0.60±0.32	-0.62±0.25	2.85±1..49	2.41±1.59	2.76±1.69	4.23±2.23
	Femoral N.	-0.72±0.33	-0.70±0.38	4.99±3.62	6.79±2.25	4.59±2.27	9.53±3.80

Decrease	Increase	Non. Significant

Table S3: Late treated CMT1A mice mRNA transcripts fold change at 4 months post intrathecal injection of AAV9.miR871. Quantitative real-time PCR analysis of huPMP22 and mu*Pmp22* as well as of mu*Mpz*, mu*Cnp*, mu *Gldn* and mu*Gjb1* gene expression in lumbar roots, sciatic and femoral nerves at 6 weeks post-injection (n=4/group). Fold relative mRNA expression levels of AAV9-miR871 injected CMT1A mice were calculated compared to AAV9-miRLacZ injected CMT1A (Mean ± SD). All samples were normalized to endogenous control *Gapdh*.

WT mice injection		Fold change of:				
		**mu *Pmp22***	**mu *Mpz***	**mu *Cnp***	**mu *Gldn***	**mu *Gjb1***
**Tissue**	**Roots**	-0.46±0.19	-0.53±0.12	0.39±0.32	0.68±0.44	0.56±0.23
	**Sciatic N.**	-0.22±0.13	-0.34±0.13	0.13±0.12	0.13±0.13	0.58±0.19
	**Femoral N.**	-0.67±0.05	-0.69±0.33	1.76±1.37	2.11±2.84	2.12±0.71

**Decrease**	**Increase**	**Non. Significant**

Table S4: WT mice mRNA transcripts fold change at 4 months post intrathecal injection of AAV9.miR871. Quantitative real-time PCR analysis of mu*Pmp22* as well as of mu*Mpz*, mu*Cnp*, mu *Gldn* and mu*Gjb1* gene expression in lumbar roots, sciatic and femoral nerves at 6 weeks post-injection (n=3/group). Fold relative mRNA expression levels of AAV9-miR871 injected CMT1A mice were calculated compared to AAV9-miRLacZ injected CMT1A (Mean ± SD). All samples were normalized to endogenous control *Gapdh*.

SUPPLEMENTAL REFERENCES

1. Boudreau, R.L., Garwick-Coppens, S. E., Liu, J., Wallace, L. M., Harper, S. Q. 2011. Rapid Cloning and Validation of MicroRNA Shuttle Vectors: A Practical Guide. In *In RNA Interference Technique*: Humana Springer Press. 19-37.
2. Wallace, L.M., et al. 2018. Pre-clinical Safety and Off-Target Studies to Support Translation of AAV-Mediated RNAi Therapy for FSHD. *Mol Ther Methods Clin Dev* 8:121-130.
3. Wallace, L.M., et al. 2011. RNA interference improves myopathic phenotypes in mice over-expressing FSHD region gene 1 (FRG1). *Mol Ther* 19:2048-2054.
4. Liu, J., et al. 2014. RNAi-mediated Gene Silencing of Mutant Myotilin Improves Myopathy in LGMD1A Mice. *Mol Ther Nucleic Acids* 3:e160.
5. McCarty, D.M., et al. 2001. Self-complementary recombinant adeno-associated virus (scAAV) vectors promote efficient transduction independently of DNA synthesis. *Gene Ther* 8:1248-1254.
6. McCarty, D.M., et al. 2003. Adeno-associated virus terminal repeat (TR) mutant generates self-complementary vectors to overcome the rate-limiting step to transduction in vivo. *Gene Ther* 10:2112-2118.
7. Huxley, C., et al. 1998. Correlation between varying levels of PMP22 expression and the degree of demyelination and reduction in nerve conduction velocity in transgenic mice. *Hum Mol Genet* 7:449-458.
8. Kagiava, A., et al. 2016. Intrathecal gene therapy rescues a model of demyelinating peripheral neuropathy. *Proc Natl Acad Sci U S A* 113:E2421-2429.
9. Kagiava, A., et al. 2018. Intrathecal gene therapy in mouse models expressing CMT1X mutations. *Hum Mol Genet* 27:1460-1473.
10. Kagiava, A., et al. 2018. Intrathecal Delivery of Viral Vectors for Gene Therapy. *Methods Mol Biol* 1791:277-285.
11. Schiza, N., et al. 2019. Gene replacement therapy in a model of Charcot-Marie-Tooth 4C neuropathy. *Brain* 142:1227-1241.
12. Kagiava, A., et al. 2019. Gene replacement therapy after neuropathy onset provides therapeutic benefit in a model of CMT1X. *Hum Mol Genet* 28:3528-3542.

13. Kagiava, A., et al. 2021. Efficacy of AAV serotypes to target Schwann cells after intrathecal and intravenous delivery. *Sci Rep* 11:23358.
14. Kagiava, A., et al. 2021. AAV9-mediated Schwann cell-targeted gene therapy rescues a model of demyelinating neuropathy. *Gene Ther* 28:659-675.
15. Rohrer, J.D., et al. 2016. Serum neurofilament light chain protein is a measure of disease intensity in frontotemporal dementia. *Neurology* 87:1329-1336.
16. Sandelius, A., et al. 2018. Plasma neurofilament light chain concentration in the inherited peripheral neuropathies. *Neurology* 90:e518-e524.
17. Jennings, M.J., et al. 2022. NCAM1 and GDF15 are biomarkers of Charcot-Marie-Tooth disease in patients and mice. *Brain*.
